# Dietary phenolic compounds as promising therapeutic agents for diabetes and its complications: A comprehensive review

**DOI:** 10.1002/fsn3.3983

**Published:** 2024-01-30

**Authors:** Dipa Aryal, Soniya Joshi, Nabin Kumar Thapa, Pratiksha Chaudhary, Sirjana Basaula, Usha Joshi, Damodar Bhandari, Hannah M. Rogers, Salyan Bhattarai, Khaga Raj Sharma, Bishnu P. Regmi, Niranjan Parajuli

**Affiliations:** ^1^ Biological Chemistry Lab, Central Department of Chemistry Tribhuvan University Kathmandu Nepal; ^2^ Department of Chemistry Florida Agricultural and Mechanical University Tallahassee Florida USA; ^3^ Paraza Pharma, Inc. Montreal Québec Canada

**Keywords:** Anti‐obesity, Enzyme Inhibition, Medicinal Plants, α‐Amylase, α‐Glucosidase

## Abstract

In the middle of an ever‐changing landscape of diabetes care, precision medicine, and lifestyle therapies are becoming increasingly important. Dietary polyphenols are like hidden allies found in our everyday meals. These biomolecules, found commonly in fruits, vegetables, and various plant‐based sources, hold revolutionary potential within their molecular structure in the way we approach diabetes and its intimidating consequences. There are currently numerous types of diabetes medications, but they are not appropriate for all patients due to limitations in dosages, side effects, drug resistance, a lack of efficacy, and ethnicity. Currently, there has been increased interest in practicing herbal remedies to manage diabetes and its related complications. This article aims to summarize the potential of dietary polyphenols as a foundation in the treatment of diabetes and its associated consequences. We found that most polyphenols inhibit enzymes linked to diabetes. This review outlines the potential benefits of selected molecules, including kaempferol, catechins, rosmarinic acid, apigenin, chlorogenic acid, and caffeic acid, in managing diabetes mellitus as these compounds have exhibited promising results in in vitro, in vivo, in silico, and some preclinical trials study. This encompassing exploration reveals the multifaceted impact of polyphenols not only in mitigating diabetes but also in addressing associated conditions like inflammation, obesity, and even cancer. Their mechanisms involve antioxidant functions, immune modulation, and proinflammatory enzyme regulation. Furthermore, these molecules exhibit anti‐tumor activities, influence cellular pathways, and activate AMPK pathways, offering a less toxic, cost‐effective, and sustainable approach to addressing diabetes and its complications.

## Graphical abstract

Polyphenols, such as kaempferol, catechins, apigenin, chlorogenic acid, rosmarinic acid, and caffeic acid, have shown their ability to serve as potential antidiabetic medicines. Also, the use of polyphenols might have a few side effects although relatively less than available medications.

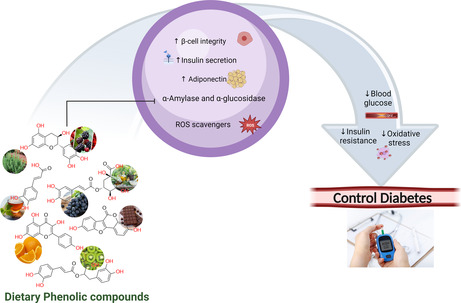

## 1 INTRODUCTION

Diabetes mellitus (DM), an intricate metabolic disorder with persistent hyperglycemia, is a global major public health concern. According to the World Health Organization (WHO), over the last 30 years, the occurrence of DM has significantly increased worldwide, and over 1.5 million deaths every year have been estimated (“Diabetes”, 2023). Diabetes is putting an enormous strain on healthcare systems and individuals globally as its incidence continues to rise unabated. Beyond elevated blood glucose levels, diabetes can lead to many consequences that impact almost every organ system, such as diabetic neuropathy, retinopathy, renal disease, and cardiovascular and macrovascular complications (Cole & Florez, 2020). The most prevalent form of diabetes is type 2 diabetes mellitus (T2DM), which covers almost 96% of total cases globally and is mainly caused by insulin resistance or insufficient insulin production (“Global Diabetes Cases to Soar from 529 Million to 1.3 Billion by 2050 | The Institute for Health Metrics and Evaluation”, 2023). Other types include type 1 diabetes mellitus (T1DM), which is the result of autoimmune death of insulin‐producing β‐cells (Lucier & Weinstock, 2023), and gestational diabetes (GD), which occurs during pregnancy due to hormonal change (McIntyre et al., 2019).

The current approach for managing diabetes primarily focuses on control through pharmacological therapies, lifestyle changes, and nutritional management. Although these approaches are beneficial to varying degrees, they frequently fall short of preventing or mitigating the complications associated with long‐term diabetes (Taylor et al., 2021). Although synthetic antidiabetic drugs are readily accessible, there is a crucial need to develop innovative and feasible antidiabetic medications due to an increase in resistance to commercial drugs and the side effects associated with their prolonged usage (Akhtar et al., 2020; Alam et al., 2022). Over the years, there has been a surge of interest in the possible use of dietary phenolic compounds, including flavonoids, coumarins, quinones, stilbenes, and curcuminoids, in diabetes management as they have been shown to increase insulin secretion and regulate blood sugar (Kooti et al., 2016). Most phenolic compounds are biologically active, plant secondary metabolites that are readily available in vegetables, fruits, whole grains, and other plant‐based sources, and they have piqued the interest of researchers due to their anti‐inflammatory, antioxidant, and metabolic regulating effects (Šamec et al., 2021). Flavonoids, including quercetin, kaempferol, baicalein, and naringenin, extracted from the bark of *Ficus racemosa* have been found to reduce glucose levels in blood from 300 to 185 mg/dL when administrated orally (100 mg/kg) for 1 week, in compared to the untreated experimental rats (Keshari et al., 2016). Recent studies show that dietary phenolic compounds may hold the key to addressing not just glycemic management but also the complex web of molecular processes that result in problems connected to diabetes. This review aims to thoroughly explore the current state of knowledge on the efficacy of various dietary phenolic compounds, which can help to prevent and treat diabetes.

## 2 BIOSYNTHESIS OF POLYPHENOLS IN PLANTS

**FIGURE 1:**
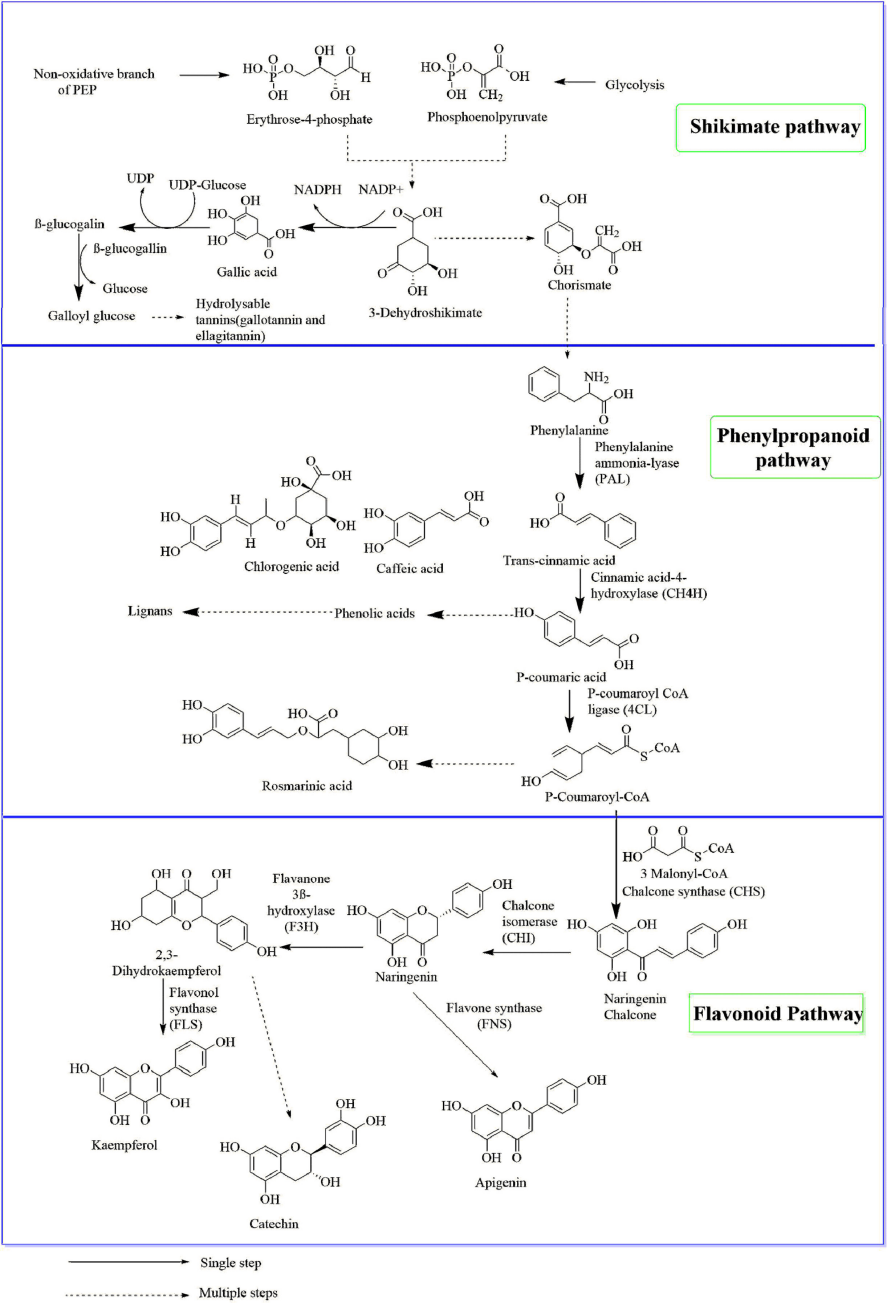
A schematic representation of three biosynthetic pathways of polyphenolic compounds. A solid arrow indicates a single‐step reaction, while a broken arrow indicates multi‐step reactions.

Phenolic compounds are biosynthesized through several pathways, including the three major routes: the shikimate, phenylpropanoid, and flavonoid pathways (Figure 1) (Patil & Masand, 2018). It is known that the shikimate pathway produces carbon frameworks for aromatic amino acid synthesis as well as various secondary metabolites further along the pathway (Bontpart et al., 2016). The first step in the process includes the chemical combination between phosphoenolpyruvate (PEP) and erythrose 4‐phosphate (E‐4P), which is catalyzed by 3‐deoxy‐d‐arabino‐heptulosonate‐7‐phosphate synthase (DAHPS; EC 2.5.1.54), to generate 3‐deoxy‐d‐arabino‐heptulosonate‐7‐phosphate (DAHP). Thus, this DAHP is converted into shikimates by the action of three enzymes, including DAHP synthase, 3‐dehydroquinate synthase, and shikimate dehydrogenase (Santos‐Sánchez et al., 2019). Phosphorylation of shikimic acid with the aid of shikimate kinase produces shikimic acid 3‐phosphate which on condensation with PEP, catalyzed by enol pyruvyl shikimate 3‐phosphate synthase, gives 5‐enol pyruvyl shikimate‐3‐phosphate (EPSP). Finally, EPSP is transformed into chorismate in the presence of chorismate synthase and completes the shikimate pathway (Bontpart et al., 2016).

The origin of phenylalanine and the gateway to the production of phenylpropanoids is the shikimate pathway and the phenylpropanoid pathway is the initial stage for the biosynthesis of several essential compounds, including flavonoids, coumarins, lignans, and other phenolic compounds (Fraser & Chapple, 2011). Phenylalanine and tyrosine are aromatic amino acids derived from chorismic acid of the shikimate pathway and are the key precursors for the phenylpropanoid pathway (Kawaguchi et al., 2017). The fundamental phenylpropanoid pathway consists of three enzyme‐catalyzed steps (Fraser & Chapple, 2011). At first, trans‐cinnamic acid is produced from phenylalanine by the action of phenylalanine ammonia‐lyase (PAL). Trans‐cinnamic acid is then converted into 4‐coumaric acid by cinnamic acid 4‐hydroxylase (CH4H). The next step involves the conversion of 4‐coumaric acid to 4‐coumaroyl‐CoA facilitated by p‐coumaroyl‐CoA ligase (Emiliani et al., 2009).

Chalcone synthase (CHS), which is a key enzyme in flavonoid biosynthesis, catalyzes a combination of 4‐coumaroyl‐CoA with three molecules of malonyl‐CoA to produce naringenin chalcone (Austin & Noel, 2003). Naringenin chalcone is then converted to various flavonoids, such as flavones, flavanones, and anthocyanins through the action of a specific enzyme (Roy et al., 2022). For example, flavone synthase (FNS) is responsible for converting naringenin to apigenin, while Fe^2+^/2‐oxoglutarate‐dependent dioxygenase mediates the direct transformation of eriodictyol and naringenin into their corresponding flavones (Li et al., 2020). The three pathways for the biosynthesis of polyphenols are shown in Figure 1.

## 3 Roles of phenolic compounds for inhibition of enzymes linked to diabetes

Inhibitors of α‐amylase (EC 3.2.1.1) and α‐glucosidase (EC 3.2.1.1) have emerged as a useful treatment for reducing postprandial hyperglycemia. These inhibitors efficiently limit carbohydrate digestion in the small intestine, resulting in a lower postprandial blood glucose excursion (Kazeem et al., 2013). Many researches have revealed that different phenolic compounds have shown potential to inhibit α‐amylase and α‐glucosidase enzymes which are shown in Table 1. We cataloged 71 polyphenols which are listed in Table S1, summarizing their dietary and plant source, mechanism of action, and IC_50_ values. The half‐maximal inhibitory concentration (IC_50_) is the concentration of an inhibitor molecule required to inhibit 50% of enzyme activity, the lower the IC_50_, the more potent the inhibitor. The chemical structure of the phenolic compounds reviewed in this article is shown in Figure S1.

**TABLE 1:** List of polyphenols from a dietary supplement that helps to inhibit diabetes‐related enzymes.

Compound	Dietary and plant sources	Mechanism of action	Enzyme(s) used	IC_50_ value	References
Kaempferol	Vegetables like (*Cucumis sativus*) cucumber, tomato, onion, broccoli, green beans, and citrus fruits	Antidiabetic action by suppressing the enzymatic activity of enzymes α‐glucosidase and α‐amylase	ɑ‐Glucosidase, ɑ‐amylase	51.24 μg/mL 29.37 μg/mL	Ibitoye et al. (2018), Lee et al. (2015)
Catechin	Strawberry (*Arbutus unedo* L.), green tea (*Camellia sinesis*), cocoa, and red wine	Antioxidant activity alleviates thirst, promotes mental relaxation, acts as a diuretic, and be employed in the treatment of cough, fatigue, and mild insomnia, anti‐inflammatory, detoxifying, and expectorant‐like activities	ɑ‐Glucosidase	87.55 ± 2.23 μg/mL	Islam et al. (2022), Silveira et al. (2019)
Apigenin	Celery, chamomile tea, parsley, celeriac	Antidiabetic, antioxidant, anti‐inflammatory activity by suppressing the enzymatic activity of enzymes α‐glucosidase and α‐amylase, blood pressure reduction, stimulate the metabolism of glucose, enhance secretion of insulin from the pancreas, ability to overcome dyslipidemia	ɑ‐Glucosidase	2.838 ± 0.014 μg/mL	El Barky (2020), Zeng et al. (2016)
Rosmarinic acid	Rosemary (*Rosmarinus officinalis*), and, Sage, Basil, Perilla, Mint	Lowers PEPCK expression and elevates GLUT4 expression, lowers hyperglycemia, and improves insulin sensitivity, antioxidant activity, ɑ‐glucosidase inhibition	ɑ‐Glucosidase	48.46 ± 0.52 μg/mL	Inui et al. (2016)
Chlorogenic acid	Apples, pears, carrots, tomatoes, sweet potatoes, eggplant, coffee, tea, blueberries, and sunflower seeds	It shows hypoglycemic and hypolipidemic effects, antibacterial, antioxidant, and anti‐inflammatory, improves insulin function, and shows antidiabetic properties	α‐Amylase	1410 ± 400 μg/mL	Aleixandre et al. (2022), Yan et al. (2020)
Caffeic acid	*Olea europea* (Olives), *Coffea arabica*, *Coffea canephora* (coffee beans), *Artocarpus heterophyllus*, *Daucus carota* (carrot), and propolis (bee glue)	Antidiabetic, antioxidant, anticancer activity	α‐Amylase, α‐glucosidase, aldose reductase	26.90 ± 0.05 μg/mL, 8.00 ± 0.40 μg/mL, 3.10 ± 0.33 μg/mL	Espíndola et al. (2019), Maradesha et al. (2022)
Quercetin	*Polygonum aviculare* L. leaves and blueberries, apples, broccoli, beans	Antidiabetic, anti‐inflammatory, and antioxidant activity, and insulin resistance, the fundamental mechanism of action was associated with a reduction in the endoplasmic reticulum stress, oxidative stress, and the loss of pancreatic β‐cell	DPP4, *α*‐glucosidase	1.150 μg/mL 15.17 ± 3.25 μg/mL	Islam et al. (2022), Silveira et al. (2019)
Genistein	Soybeans (*Glycine max*) red clover (*Trifolium pratense*), other legumes, and Kudzu root (*Pueraria radix*)	Decrease in hepatic glucose production by maintaining insulin‐positive β‐cells and modifying the hepatic glucose	Tyrosine kinase	2.1619 μg/mL	Choi et al. (2008), Silveira et al. (2019)
Taxifolin	*Rhizoma Smilacis glabrae*, and red onion, milk thistle, acai palm, pigmented rice, orange, grapefruit	The antihyperglycemic impacts are critical in the management of postprandial hyperglycemia	α‐Amylase, α‐glucosidase, pancreatic lipase	647 μg/mL, 38 μg/mL, 993 μg/mL	Su et al. (2020)
Curcumin	Root of *Curcuma longa* and *Berberis aristata*, *Lagerstroemia speciose*	Antioxidant, anti‐inflammatory, and anticancer activity, blood glucose and lipid levels are reduced, liver and kidney function improved, and glucose and lipid homeostasis improved.	ɑ‐Amylase	18.905 μg/mL	Den Hartogh et al. (2020), Silveira et al. (2019)

### 3.1 Inhibition of α‐amylase

**FIGURE 2:**
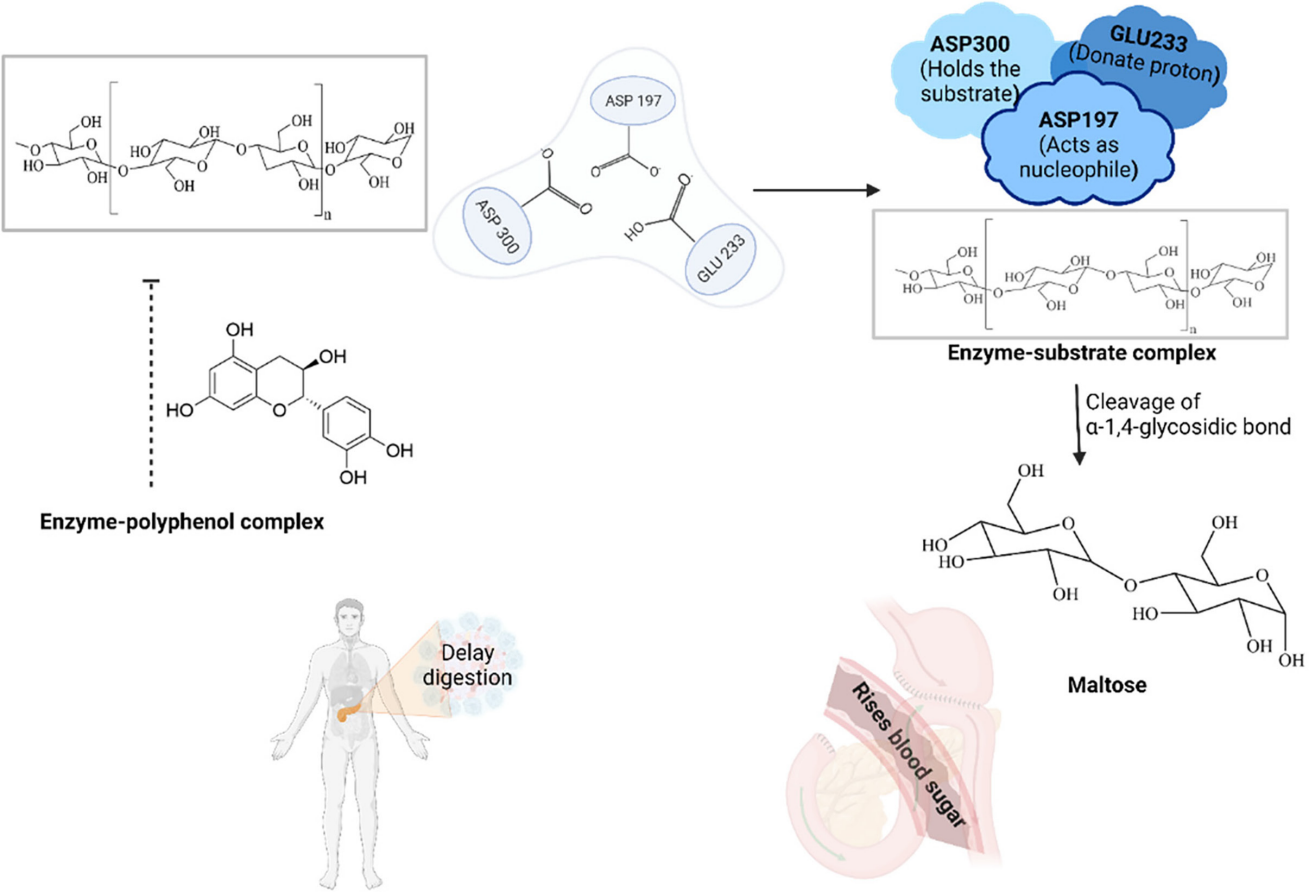
Phenolic compounds on starch digestion by hydrolysis of 1,4‐glycosidic bond.

Alpha‐amylase is a crucial digestive enzyme that hydrolyzes 1,4‐glycosidic bonds in starch and other polysaccharides to yield glucose, maltose, and other maltooligosaccharides (Dey et al., 2002; Terra & Ferreira, 2005). Inhibition of this enzyme can minimize the subsequent rise in plasma glucose, making it a viable approach in the management of post‐meal blood sugar levels (Tundis et al., 2010). Some naturally occurring phenolic compounds exert their inhibitory effects on α‐amylase through a variety of mechanisms like competitive, uncompetitive, and mixed‐type inhibition (Sun, Wang, et al., 2020). Dietary polyphenols such as anthocyanins (cyanidin 3‐glucoside) have shown the ability to boost insulin‐mediated glucose absorption and they activated AMPK (activated protein kinase) in the skeletal muscle and liver of type 2 diabetic mice, which leads to reduced blood sugar levels and enhancing insulin sensitivity (Sasaki et al., 2007). Phenolic compounds substantially influence the α‐amylase, but their mechanism of action has not been studied yet in detail (Sun, Wang, et al., 2020). Two mechanisms exist by which phenolic compounds interact with α‐amylase, the first one is the hydrophobic interactions between the tryptophan residue in α‐amylase and the aromatic rings of polyphenols and the other is the hydrogen bonding between the hydroxyl groups of phenolic compounds and the amino acid residue of the enzyme's active site (Sun et al., 2019). X‐ray crystallography study of porcine pancreatic α‐amylase identified three key amino acid residues, namely, Asp197, Glu233, and Asp300 situated in the active site, as well as aromatic residues like Trp58, Trp59, and Tyr62 aligned with the entrance of the enzyme's active site (Sun, Wang, et al., 2020), in which Asp197 acts as a nucleophile, Glu233 as a proton donor, and Asp300, holds the substrate. The inhibitors bind to the same catalytic triad to inhibit the enzyme activity or slow down the hydrolysis of carbohydrates (Bompard‐Gilles et al., 1996) as shown in Figure 2. The electron delocalization between aromatic rings and C=C (or C=O), for example, in flavonols and flavones enhanced hydrophobic (π–π) interactions with α‐amylase (Lo Piparo et al., 2008). This strengthens the bond with α‐amylase and leads to a decrease in its catalytic activity.

**FIGURE 3:**
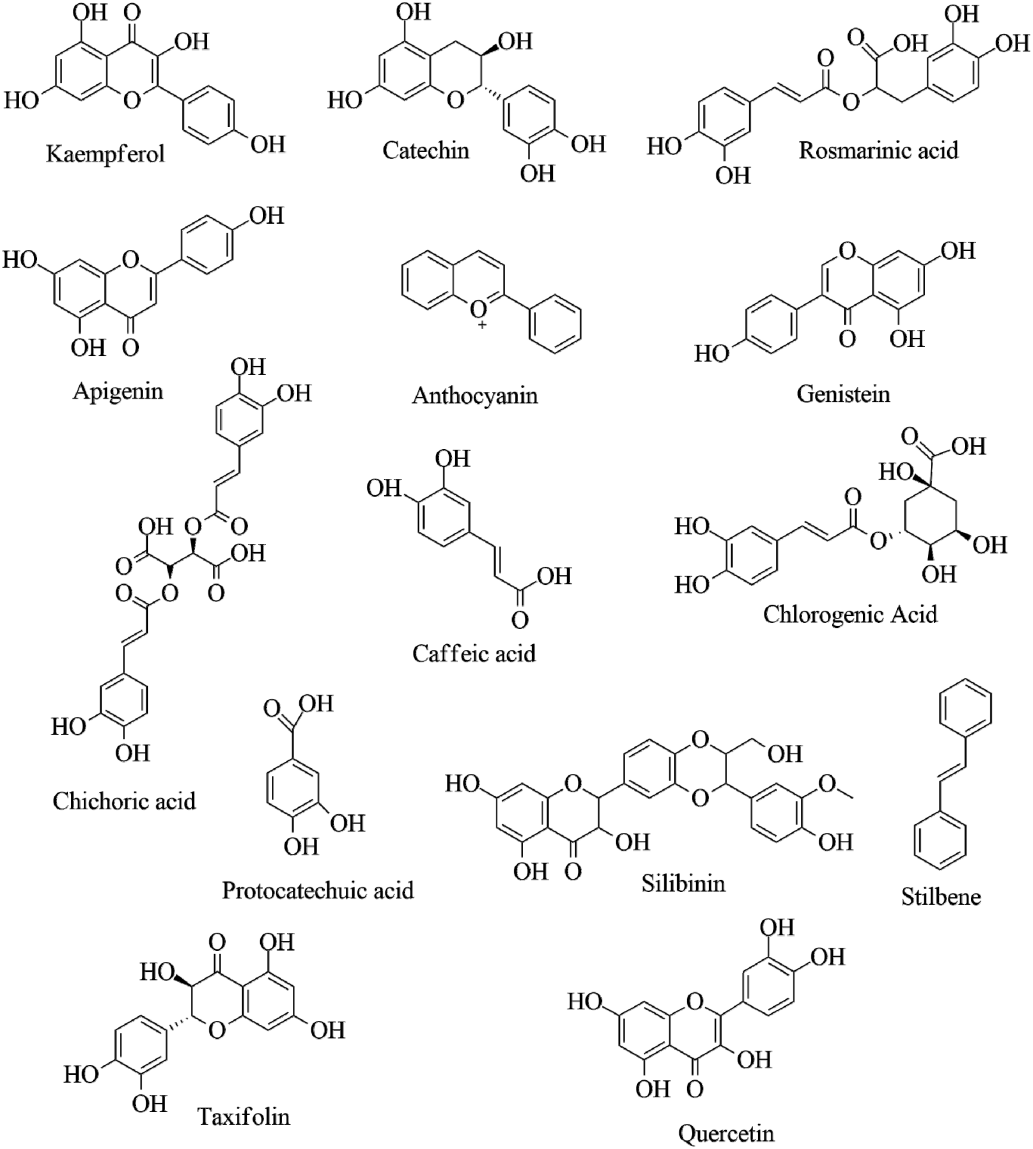
Chemical structures of phenolic compounds exhibiting inhibition α‐amylase and α‐glucosidase.

Proença et al. (2019) found that the OH group in the flavone at 5‐ as well as 7‐positions in the A‐ring, 3′‐ and even 4′‐positions in the ring B, and Cl‐ in the 3‐position in the C ring as the most effective flavonoid examined for the inhibition of α‐amylase. Particularly for flavonoids like quercetin, as shown in Figure 3, the presence of OH at the 5‐, 6‐, and 7‐positions of ring A as well as the 4′ position of ring B can increase the inhibitory action because ‐OH is essential for the formation of hydrogen bonds with the active site of the enzyme (Lo Piparo et al., 2008; Miao et al., 2014). Yilmazer‐Musa et al. discovered in their investigation that the polyphenolic‐rich grape seed extracts strongly inhibited α‐amylase with the same potency as acarbose (a clinically approved medication, that acts as an anti‐α‐amylase agent) (Yilmazer‐Musa et al., 2012). Rehman et al. (2019) have demonstrated that taxifolin exhibits a substantial inhibitory action on α‐amylase, surpassing the inhibitory effects of acarbose in their in vivo and in silico studies. An intriguing investigation was conducted to determine the explanation for the variance in the inhibitory action of tannic acid against α‐amylase which concluded that the substrate concentration (natural starch, artificial polymer of anhydroglucose) and the physiochemical characteristics of the substrates are important parameters influencing the inhibitory activity of a polyphenol (Zhang et al., 2022).

### 3.2 Inhibition of α‐glucosidase

Alpha‐glucosidase catalyzes the hydrolysis of saccharides into glucose by breaking the α‐glycosidic linkages within the substrates depending on their specificity—disaccharides and oligosaccharides (Truscheit et al., 1981). Trp516 and Asp518 are the primary catalytic sites of α‐glucosidase where the substrate binds and the hydrolysis of carbohydrates takes place (Hermans et al., 1991).

Alpha‐glucosidase inhibitors (AGIs), which are oral antidiabetic medications, prevent the digestive enzymes of the upper gastrointestinal tract from breaking apart complex carbohydrates into glucose. Consequently, postprandial glucose is lowered, glucose absorption is delayed, and glycemic control is improved (Hedrington & Davis, 2019). In recent developments concerning DM treatment, there is a growing interest in AGIs derived from various plant‐based bioactive compounds as they are gaining interest for their ability to suppress α‐glucosidase activity. This inhibition leads to a decrease in the breakdown of dietary oligosaccharides and reduces the release of α‐glucose, which delays carbohydrate digestion and glucose absorption in the small intestine (Hossain et al., 2020). Acarbose is a competitive inhibitor of α‐glucosidase with the inhibition dependent on substrate concentration, while the phenolic extracts of various plants show non‐competitive inhibition which is independent of substrate concentration (Zhang et al., 2015). The relationship between the structure and activity of phenolic compounds with α‐glucosidase activity demonstrates that hydroxylation of flavonoids can be beneficial for increasing their inhibitory impact on the enzyme, whilst methoxylation, glycosylation, and reducing the C_2_ = C_3_ double bond may diminish the inhibitory activity (Li et al., 2023). Several naturally occurring and synthetic phenolic compounds; coumarins, chromones, and their derivatives, including luteolin, naringenin, anthocyanins, and baicalein, have been targeted as powerful AGIs (Al‐Salahi et al., 2018). Researchers have found that the functional groups (hydroxyl and benzene rings) of tea polyphenols interact with amino acid residues at the active site of α‐glucosidase through the formation of hydrogen bonds and hydrophobic stacking occurring either in parallel or vertical conjugation (Sun et al., 2021). Studies carried out by Su et al. indicated that taxifolin exhibited a dose‐dependent inhibition of α‐glucosidase activity, with an IC_50_ value of 0.038 mg/mL in contrast to an IC_50_ value of 0.917 mg/mL for the commonly used positive control, acarbose as shown in Table 1. This shows that taxifolin is more effective than acarbose in inhibiting α‐glucosidase (Su et al., 2020).

Consequently, dietary phenolic compounds have emerged as promising candidates for inhibiting diabetes enzymes, such as α‐amylase and α‐glucosidase. However, more preclinical and clinical research is required to determine their safety, effectiveness, and long‐term impacts. Table 1 is a summary of major polyphenols that inhibit diabetes‐related enzymes.

## 4 PROMISING DIETARY PHENOLIC COMPOUNDS

### 4.1 Kaempferol

Kaempferol, a naturally occurring flavanol, is present in several plants such as apples, broccoli, onions, tomatoes, green beans, citrus fruits, grapes, and *Ginkgo biloba* (Devi et al., 2015). Numerous studies in animals have demonstrated how kaempferol can regulate critical genes within cell signaling pathways linked with inflammation, metastasis, apoptosis, and angiogenesis (Chen & Chen, 2013). Furthermore, there have been multiple investigations indicating that kaempferol has pharmacological functions, including antidiabetic, anti‐inflammatory, antioxidant, antimicrobial, neuroprotective, and antiosteoporotic, along with antiallergic activities (Lee et al., 2015). The in vitro study showed that kaempferol effectively inhibited α‐glucosidase and α‐amylase with an IC_50_ value of 2.33 and 52.95 μg/mL, respectively (Sheng et al., 2018). In diabetes‐induced mice via streptozotocin, kaempferol administration enhanced hexokinase function in the red skeletal muscle and liver, thereby increasing its metabolism of blood sugar and it also diminished hepatic glucose generation by significantly reducing the elevated activity of pyruvate carboxylase, which catalyzes an initial committed phase in hepatic lipogenesis (Alkhalidy et al., 2018). Also, the abnormal activity of membrane‐bound ATPases was restored (Al‐Numair et al., 2015). Kaempferol made no difference in insulin secretion, yet it exhibited antidiabetic effects by improving insulin sensitivity and suppressing hepatic gluconeogenesis by preventing pyruvate carboxylase and glucose‐6‐phosphatase activity (Alkhalidy et al., 2018). It is an excellent antioxidant prevents oxidative damage in pancreatic beta cells induced by glucotoxicity and promotes autophagy that protects β‐cells from lipotoxic harm by maintaining lipid homeostasis (Yang et al., 2022). Moreover, it counteracts inflammation and oxidative stress in the heart and retinal pigment epithelium cells and alleviates cardiomyopathy and retinopathy which are correlated with diabetes (Feng et al., 2017). In accordance with pharmacophore studies, drug‐likeness, and in silico screening of phytochemicals of *Phyllanthus emblica* revealed that kaempferol is one of the probable antidiabetic candidates (Sharma et al., 2020). Dogan and Anuk (2019) found that the leaves of *Platanus orientalis* containing kaempferol and derivatives, essential fatty acids, and benzaldehyde enhanced weight loss, hepatoprotection, nephroprotection, and blood parameters in rats subjected to ethanol‐induced oxidative stress (Dogan & Anuk, 2019). While more investigations are required to validate these results and establish the ideal dose and time frame for kaempferol intake for diabetes management it can be an excellent candidate to mitigate diabetes and its complications.

### 4.2 Catechins

Catechins are polyphenols that are abundantly found in tea, cocoa, as well as berries and have been shown to regulate processes in the pancreas and gastrointestinal tract, thereby affecting glucose homeostasis (Cremonini et al., 2019; Sharma et al., 2020). They increase the rate of mitochondrial oxidative phosphorylation followed by ATP production, as well as biogenesis of the mitochondria and β‐cell apoptosis mediated by mitochondria‐associated enzymes, to improve glucose‐stimulated insulin secretion (Wen et al., 2022). According to recent studies, catechins extracted from the fruits of *Elaeagnus umbellata* lowered fasting blood sugar levels in diabetic mice, inhibited key carbohydrate‐digesting enzymes, and showed antioxidant properties with high inhibitory capacity (α‐amylase; 83 ± 1.5%, α‐glucosidase; 85 ± 1.1%) (Nazir et al., 2020). The adverse metabolic effects of streptozotocin‐treated rats were substantially and dose‐dependently reversed by intraperitoneal injection of catechins, thereby decreasing serum glucose levels and improving lipid profiles (Samarghandian et al., 2017). In a clinical trial, individuals were allowed to consume oolong tea and green tea enriched with catechins for 12 weeks, and results showed significant positive effects, including a decrease in body weight, lipid peroxidation, fat, and an improvement in lipid profile, oxidative indices, and antioxidant enzymes (Venkatakrishnan et al., 2018). In another clinical trial, individuals with T2DM who were not receiving insulin treatment were given 582.8 mg of catechin‐rich drinks daily for 12 weeks. At week 12, there was an increase in insulin and adiponectin. This demonstrates that catechins may be able to prevent diabetes and obesity (Nagao et al., 2009). In silico study conducted in *Spondias mombin* displayed catechins to be the most chemically inert molecules among other compounds in terms of HOMO/LUMO calculation, demonstrated exceptional drug‐like pharmacokinetics, and exhibited strong affinity with glycogen synthase kinase 3β (GSK3β), indicating them as potential drug candidates (Ajiboye et al., 2023). All these studies indicate that catechins can be potent phytochemicals to work against diabetes mellitus.

### 4.3 Apigenin

Parsley, celery, grapefruit, and chamomile plants contain a polyphenolic flavone called apigenin, which is less toxic and non‐mutagenic than other flavones (Balez et al., 2016). In T2DM rats, apigenin could substantially ameliorate increased resistance to insulin, impaired glucose and metabolism of lipids, diabetic vasculopathy, and endothelial function (Ren et al., 2016). This effect could be partially achieved by reducing oxidative stress and inflammation linked to T2DM. Nitric oxide, which apigenin can supply to endothelial cells, effectively prevents or mitigates the damaging effects due to enhancement in blood glucose levels (Wang, Cheng, et al., 2014). Additionally, apigenin can control free radicals associated with diabetes and protect against pancreatic damage (Yusni et al., 2018). It can also regulate AMP‐activated protein kinase pathways, which control glucose absorption, carbohydrate ingestion, and raise glucose levels in skeletal muscle cells (Ebrahimi et al., 2017). Apigenin was found to be an immunotherapeutic drug, which inhibits both the enzymes *α*‐glucosidase and *α*‐amylase, by comparing findings of an in silico study utilizing docking assessments followed by enzymatic assays demonstrating in vitro inhibitory efficiency (Tolmie et al., 2021). During the in silico study of apigenin with glucose transporter 4 (GLUT‐4) receptor, an interesting observation was made. A noticeable contact with GLU270, a residue located within the binding cavity of GLUT‐4, was consistently detected (Annapurna et al., 2013). It showed an IC_50_ value of 2.838 ± 0.014 μg/mL against enzyme α‐glucosidase (Zeng et al., 2016). The blood–brain barrier (BBB) test, the AMES test (presence of mutagenic chemicals), and hepatotoxicity, for testing of drugs and toxicity, all showed no positive results for apigenin derivatives; as a consequence, they might be employed in potential treatments for type 2 diabetes (Ahmed et al., 2022). According to these investigations, apigenin may have the ability to treat diabetes.

### 4.4 Rosmarinic acid

Rosmarinic acid, a typical ester that is formed from caffeic acid and (R)‐(+)‐3‐(3,4‐dihydroxyphenyl)lactic acid, can build up in significant proportions in a range of plant species (Amoah et al., 2016). This polyphenol belongs to the plants of the Labiatae family, including perilla, sage, rosemary, and, sweet basil (Petersen, 2003). It has been demonstrated that this compound enhances insulin sensitivity by reducing glucose levels through upregulation of GLUT4 and downregulation of phosphoenolpyruvate carboxykinase (PEPCK) (Inui et al., 2016). The uptake of glucose in muscles can be boosted by treating L6 muscle cells with rosmarinic acid through the activation of AMPK (Vlavcheski et al., 2017). The in vitro α‐glucosidase and antioxidant assays carried out using rosmarinic acid and its derivatives demonstrated great potential to be developed as an antidiabetic agent as it showed a low IC_50_ value compared to that of standard drug acarbose (Cardullo et al., 2019). In recent studies, rosmarinic acid isolated from *Plectranthus amboinicus* leaves showed a significant reduction in blood sugar and glycated hemoglobin, raised the plasma glucose and hemoglobin content, and rise in the glycogen level in the liver and muscles as it reactivated damaged glycogen phosphorylase and glycogen synthase (metabolic enzymes) when injected into diabetic rodents for 30 days period (Ramalingam et al., 2020). Enzymatic assays were used to compare the in vitro inhibition efficacy to the inhibitory power of the enzyme determined by in silico docking analysis. Rosmarinic acid showed the lowest hERG (the human ether‐a‐go‐go‐related gene) toxicity when docked onto the active site of both α‐amylase and α‐glucosidase enzymes, where it interacted with amino acids via a negative value of binding energy, which indicates a spontaneous interaction with amino acids (Tolmie et al., 2021). Hence, we can conclude that rosmarinic acid has the potential to be used as an antidiabetic agent.

### 4.5 Chlorogenic acid

Chlorogenic acid (CGA), a phenolic compound typically present in apples, pears, carrots, tomatoes, coffees, teas, and sunflower seeds, serves as an intermediary compound in lignin production. It exhibits hypoglycemic actions, enhances insulin efficiency, and lessens insulin resistance (Yan et al., 2020). The liver's glucose‐6‐phosphatase is competitively inhibited by CGA, and this slows down the hydrolysis of hepatic glycogen. In liver tissues, CGA caused the levels of glutathione, superoxide dismutase (SOD), and catalase to rise, while malondialdehyde levels to drop, and thus, showed its potential to work by suppressing oxidative stress (Shi et al., 2016). CGA lowers the absorption of glucose in the stomach by inhibiting G‐6‐phosphate translocase and sodium‐glucose cotransporter (Meng et al., 2013). The presence of major compounds, such as quinic acid, cyranoside, CGA, and cosmosiin in the *Achillea arabica* flower extract, lyophilized in ethanol showed significant antioxidant effects in streptozotocin‐induced diabetic rats, reducing blood glucose, serum glucose, HbA1c, liver, and kidney damage biomarkers (Hanalp et al., 2023). A research article by Kato et al. has concluded that elderly people who report experiencing subjective memory loss may benefit from taking CGAs for 6 months if their executive, memory, and attentional abilities are impaired (Kato et al., 2018). Farrell et al. studied the absorption and metabolism of chlorogenic acid in gastric epithelium monolayers, finding relevant evidence of methylation of gastric mucosa cells which in turn suggested a potential mechanism for its gastrointestinal metabolism. This finding highlights its antidiabetic potential by regulating glucose absorption, suppressing glycogenolysis, and maintaining insulin sensitivity (Farrell et al., 2011). CGA can greatly reduce the development of insulin resistance in mice by up‐regulating GLUT‐4 transcript and down‐regulating G‐6‐Pase mRNA expression (Xie, Fei, et al., 2021). According to Yan et al., around 30% of dietary CGA is metabolized in the small intestine, showing that CGA can enter the large intestine and exhibit direct effects on microorganisms. The authors investigated the changes in the microbiota composition of diabetic mice induced by CGA, mediating the positive effects of CGA on metabolic disorders (Yan et al., 2022). The thermodynamics of the CGA's spontaneous binding to subdomain IIA of HSA are further revealed by the results of docking and modeling and the results of the study point to the possibility that chlorogenic acid might benefit in the prevention of diabetes‐related issues and its progression (Siddiqui et al., 2023). All these studies suggest that CGA plays a crucial role in glucose metabolism; however, the mechanism is not well known.

### 4.6 Caffeic acid

Caffeic acid is a primary hydroxycinnamic acid and a polyphenol that is commonly found in the human diet (Verma & Hansch, 2004). Caffeic acid shows remarkable physiological actions, including, antiviral, cardioprotective, antioxidant, and antibacterial activities (Verma & Hansch, 2004). Also, caffeic acid has exhibited pharmacological antioxidant activity and streptozotocin‐induced antidiabetic action in rats (Okutan et al., 2005). Caffeic acid controls GLUT‐4 activity in adipocytes and beta‐cells, as well as glucokinase activity in hepatocytes (Espíndola et al., 2019). It also inhibits phosphoenolpyruvate carboxykinase and glucose‐6 phosphatase, reduces glycosylated hemoglobin, and increases glucokinase activity, all of which contribute to controlled DM (Oršolić et al., 2021). Caffeic acid also demonstrated active potential as an intestine *α*‐amylase and *α*‐glucosidase inhibitor throughout the in vitro experiment (Adisakwattana et al., 2009). In the study of STZ‐induced diabetic rats, oral treatment of caffeic acid at a dose of 40 mg/kg lowered fasting blood glucose, cholesterol, and triglycerides and substantially mitigated kidney damage. The diabetic kidney's histological parameters were also improved by caffeic acid (Salem et al., 2019). In molecular docking studies, caffeic acid demonstrated significant inhibitory potential against both *α*‐glucosidase and *α*‐amylase enzymes (Shanak et al., 2021). The auto dock binding energy values were observed at −5.45 kcal/mol and −5.25 kcal/mol, and RMSD values of 10.935 Å and 68.360 Å for *α*‐glucosidase and *α*‐amylase, respectively, indicating a strong and potent inhibition against both target enzymes. Analysis of these studies indicates that caffeic acid is a potent compound to mitigate diabetes complications.

## 5 ACTIVITIES OF POLYPHENOLS FOR THE MITIGATION OF DIABETES MELLITUS

### 5.1 Antioxidant activity

**FIGURE 4:**
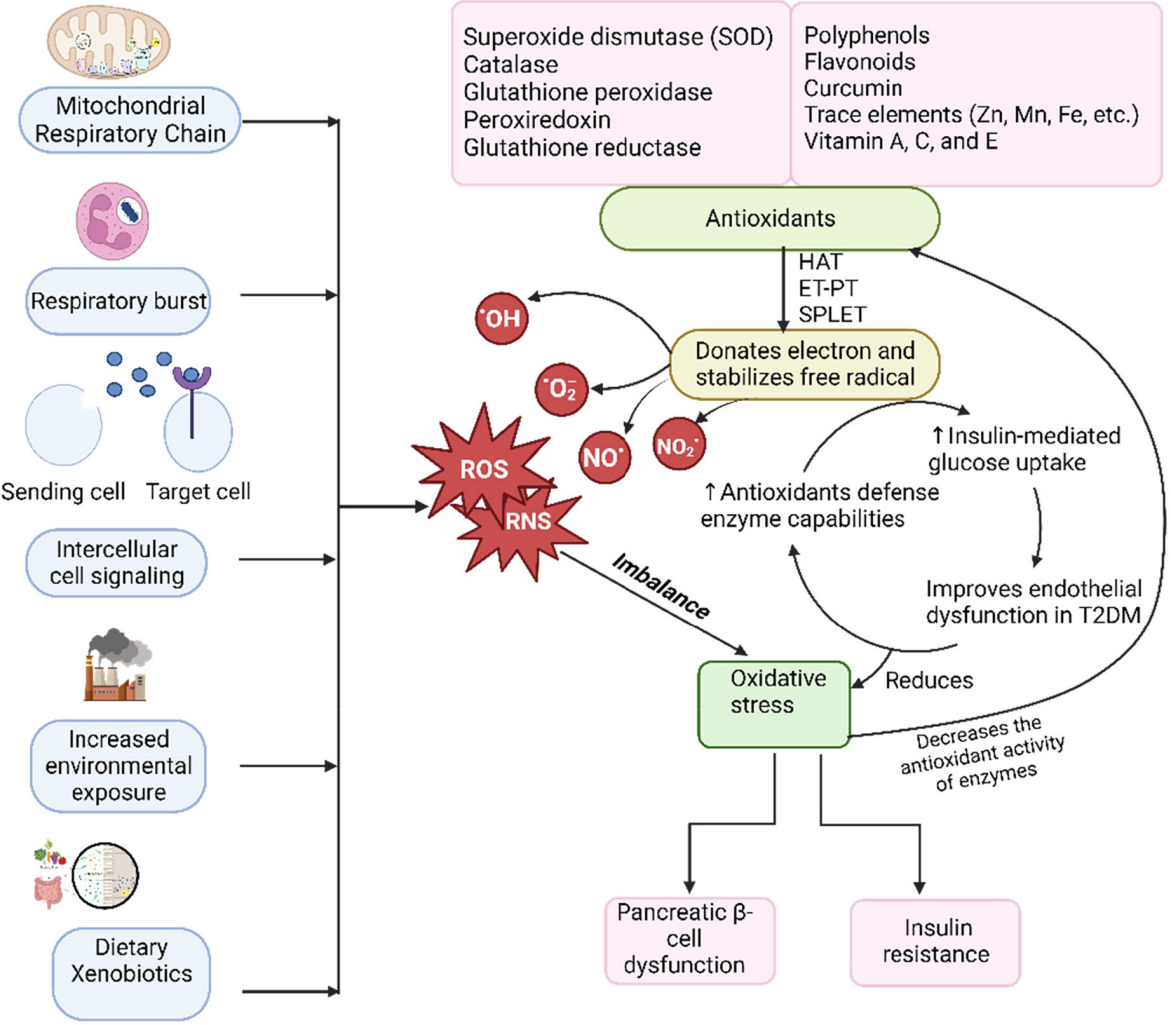
Mechanism of action of antioxidants from phytochemicals on diabetes mellitus. The excess production of reactive free radicals through different processes induces oxidative stress, which disrupts the pancreatic β‐cells and develops insulin resistance. Antioxidants stabilize free radicals by donating electrons, increasing glucose uptake, and recovering endothelial dysfunction, thereby reducing oxidative stress.

Antioxidants can slow down or stop the oxidation of substrate in an organism, thereby reducing the damaging effects of oxidative stress (Nikinmaa, 2014). Numerous studies show that oxidative stress significantly contributes to the development of diabetes including dysfunction of pancreatic β‐cell, insulin resistance, and the elevation in the risk of complications (Asmat et al., 2016; Nowotny et al., 2015) as illustrated in Figure 4. Natural antioxidants like phenolic substances are scavengers of reactive oxygen species (ROS) and remove ROS through three different mechanisms: transfer of a hydrogen atom (HAT), one electron transfer coupled with proton loss (ET‐PT), and subsequent proton loss coupled with an electron transfer (ET‐PT) (Wang et al., 2020). Superoxide dismutase (SOD), catalase, and glutathione peroxidase are the enzymes that scavenge free radicals or molecules; glutathione, thioredoxin, and peroxiredoxins are the antioxidants that are not enzymatic, which inhibit the radical formation and protects biomolecules from redox imbalance. A significant fraction of phenolic chemicals, which are powerful antioxidants capable of influencing numerous aspects of illnesses like lipid peroxidation in diabetes, is typically present in plant extracts (Bello et al., 2021). As a result, antioxidants derived from phenolic compounds have gained significant attention. Research is still being done to discover new natural sources of potent antioxidant compounds for minimizing side effects, which can aid in the prevention of oxidative damage in the body (Dudonné et al., 2009). In the study by Naz et al., resveratrol, a naturally occurring phenolic compound, was found to reduce oxidative stress, decrease islet fibrosis and destruction, restore islet architecture, improve islet structure and function, and attenuate other worsening changes in db/db mice, an animal model for type II diabetes with decreased ‐cell mass (Naz et al., 2023). In addition, curcumin also can lessen oxidative stress and the production of ROS, two factors that are essential for the treatment of inflammatory disorders like diabetes that are brought on by oxidative stress and inflammation (Liu et al., 2018). Due to its chemical composition and potent antioxidant capabilities, curcumin naturally scavenges free radicals. Protocatechuic acid, pyrogallol, caffeic acid, gallic acid, and propyl gallate are phenolic compounds that contain multiple hydroxyl groups. These phenolic compounds outperformed monophenols in terms of their ability to scavenge free radicals because the number and placement of these hydroxyl and methoxy groups in the phenolic rings affected their ability to donate hydrogen (Mathew et al., 2015). The working mechanism of antioxidants derived from phytochemicals is shown in Figure 4.

### 5.2 Anti‐obesity

**FIGURE 5:**
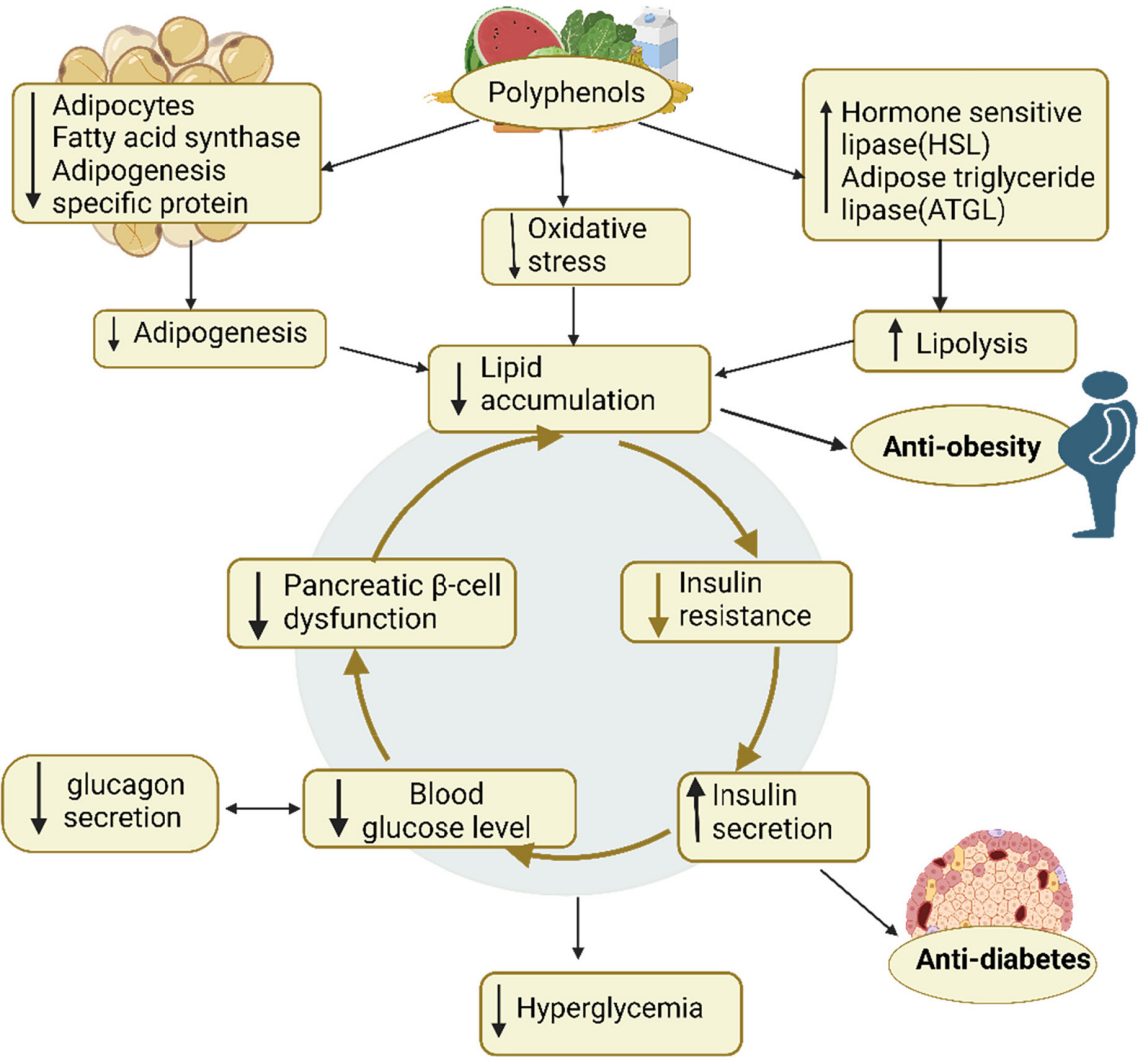
Mechanism of action of polyphenols on diabetes and obesity. Polyphenols reduce oxidative stress by decreasing adipocyte levels and increasing hormone‐sensitive lipase, thereby decreasing adipogenesis and increasing lipolysis resulting in the low accumulation of lipids reflecting its anti‐obesity ability. Low lipid accumulation results in less glucagon secretion and low blood glucose levels, showing its antidiabetic ability.

The word “diabesity” has been used to refer to the coexistence of T2DM and obesity, recognizing that an excess of body fat is the primary contributor to diabetes (Astrup & Finer, 2000). A preventable lifestyle of energy imbalance, where the number of calories ingested exceeds the amount of energy expended, has led to an alarming global epidemic of obesity (Pedersen, 2009). The deposition of a larger amount of fat than lean mass is referred to as excess adiposity, which is mainly caused by to imbalance in energy (Patwardhan & Puranik, 2012). A sharp rise in obesity triggers a corresponding rise in the prevalence of diabetes, and since weight gain or reduction is strongly correlated with either a reduction or an improvement in insulin sensitivity, respectively; research indicates that the link between body fat and glucose resistance is most certainly a cause and effect connection (Bak et al., 1992). Dietary phenolic compounds may be used as anti‐obesity medicines because they may block preadipocyte formation, increase lipolysis, and cause the death of existing adipocytes, which would reduce the bulk of adipose tissue (Mohamed et al., 2014). They can reduce the risk of T2DM by lowering obesity, which is a form of low‐grade chronic inflammation that contributes to insulin resistance (Chen et al., 2022). Phenolic compound anthocyanins demonstrate anti‐inflammatory properties that can treat chronic hyperglycemia (Sun et al., 2014). Anti‐obesity properties of dietary phenolic compounds may be mediated by a variety of methods, including inhibition of enzymes, suppression of neuro‐hormones that regulate appetite and satiety, and promotion of mitochondrial biogenesis (Aloo et al., 2023). Phenolic compounds possess the capacity to activate AMPK and peroxisome proliferator‐activate receptor α (PPARα), leading to the inhibition of acetyl‐CoA carboxylase (ACC) and fatty acid synthase, both of which are enzymes responsible for the production of long‐chain fatty acids (Singh et al., 2020; Wang et al., 2022). Consequently, this leads to a decrease in the synthesis of fatty acids and the oxidation of fatty acids increases. Peng et al. reported that *Solanum nigrum L*. water extract (SWE) rich in polyphenols decreased levels of blood cholesterol, triacylglyceride, and low‐density lipoprotein (LDL)‐cholesterol in mice given high‐fat supplements (Alappat & Awad, 2010; Peng et al., 2020). There was evidence to support the idea that regularly consuming catechins might help to prevent weight gain or the emergence of chronic disorders such as T2DM or metabolic syndrome (Sun, Zhao, et al., 2020). Serna et al. discovered that consuming 500 mg of phenolic extract of blended *Lippia citriodora* and *Hibiscus sabdariffa* daily for 60 days in overweight persons resulted in a substantial decrease in appetite feeling as well as an improvement in lipid profile (Serna et al., 2022). The role of polyphenols, demonstrating their ability to control diabetes and obesity through various processes, is summarized in Figure 5.

### 5.3 Anti‐inflammatory activity

**FIGURE 6:**
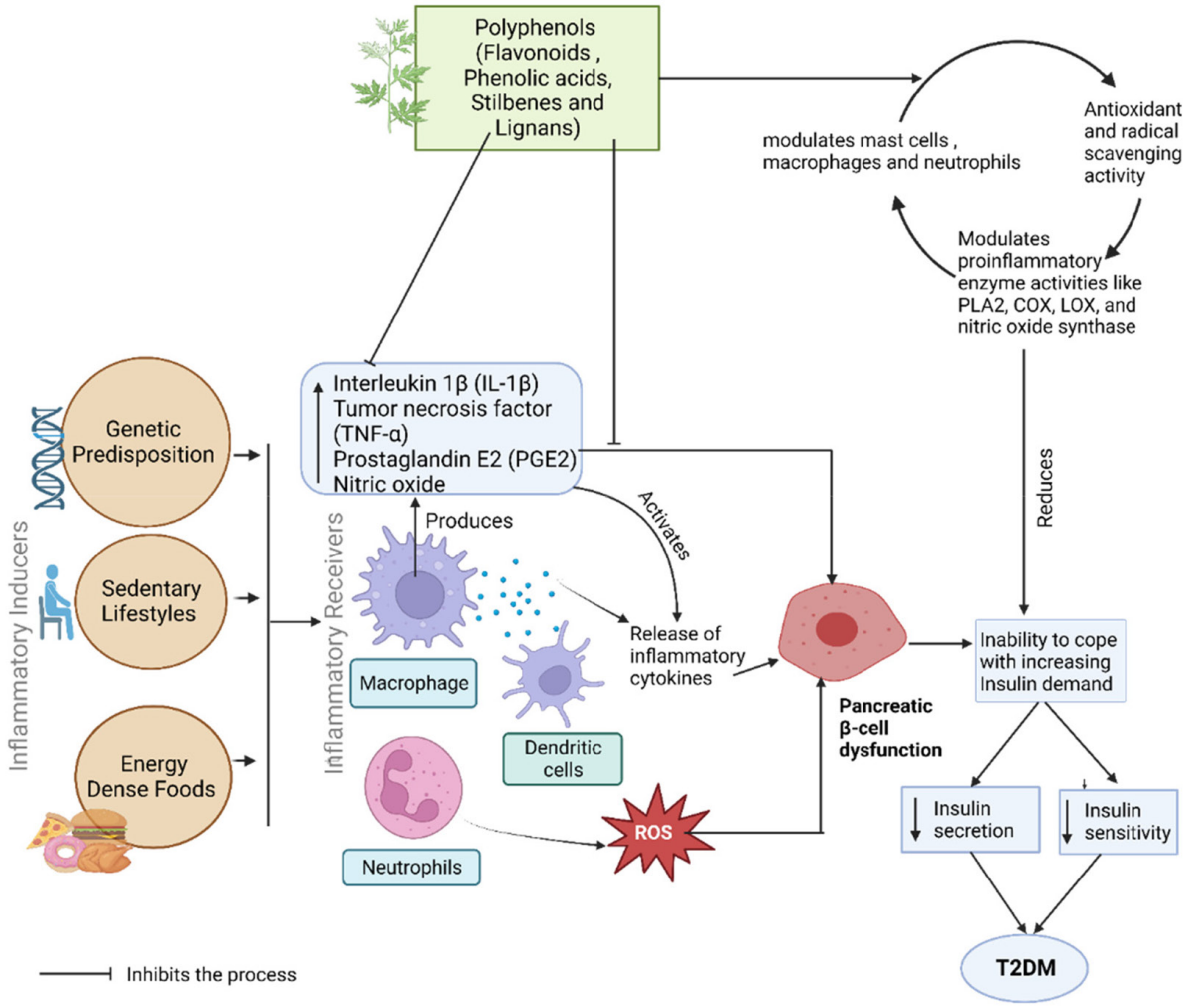
Anti‐inflammatory actions of phytochemicals derived from natural products on diabetes. Phytochemicals like flavonoids, stilbenes, and alkaloids inhibit the inflammatory cytokines produced by macrophage and dendritic cells through different mechanisms, including the modulation of mast cells, macrophages, neutrophils, and proinflammatory enzyme activities like nitric acid synthase, COX, PLA2, and LOX The release of cytokines increases insulin demand leading to T2DM.

Inflammation, a pathophysiological phenomenon controlled by the activation of various immune cells, includes several mediators of inflammation, including interleukin‐1β (IL‐1β), IL‐6, tumor necrosis factor (TNF‐α), prostaglandin E2, and nitric oxide (NO) (Lu & Yen, 2015). Impaired insulin sensitivity and associated metabolic abnormalities, including T2DM, are linked to chronic inflammation. Numerous risk factors, both unalterable (such as genetic propensity) and alterable (like sedentary lifestyles and energy‐dense foods), are being proposed to explain the processes that lead to the onset of inflammation (Ellulu & Samouda, 2022). Individuals with T2DM have elevated levels of sialic acid, acute‐phase proteins like C‐reactive protein (CRP), serum amyloid A, plasminogen activator inhibitor, fibrinogen, and haptoglobin, along with chemokines, as well as cytokines like IL‐1, IL‐6, and CRP (Donath & Shoelson, 2011). Phytochemicals with anti‐inflammatory characteristics that lessen chronic inflammation can be effective treatment options for several inflammatory illnesses (Shin et al., 2020). Phenolic compounds, such as non‐flavonoids polyphenols (emodin, resveratrol, epigallocatechin gallate, curcumin, ellagic acid, baicalin, hesperetin, naringenin, chrysin), alkaloids (berberine), and flavonoids polyphenols (kaempferol, genistein, apigenin, eriodictyol, quercetin) are used for the treatment of inflammation related to DM (Kong et al., 2021). As shown in Figure 6, several mechanisms have been proposed to explain how phenolic compounds have anti‐inflammatory properties, and these mechanisms include (1) antioxidant and radical‐scavenging functions; (2) modulation of immune cells (lymphocytes, mast cells, neutrophils, and macrophages) that regulate the cellular processes; and (3) regulation of the functions of the proinflammatory enzymes. As a result, all these mechanisms lead to an increase in the production of anti‐inflammatory mediators, reduce the production of pro‐inflammatory cytokines, and suppress the inflammatory reaction (Bellik et al., 2012). Avenanthramides, a phenolic substance isolated from oats, showed anti‐inflammatory activities by reducing the breakdown of inhibitor and activated kappa B‐cells nuclear factor light chain enhancer (NF‐𝜅B), which is associated with the reduction in the phosphorylation in the p65 component of NF‐𝜅B. Additionally, it is demonstrated as a critical suppressor of TNF‐α and IL‐8 production (Sur et al., 2008). By lowering serum levels of interferon (IFN) and helper T (Th) 1 cell percentage, resveratrol treats glomerulonephritis in systemic lupus erythematosus (SLE) mice caused by pristine (Wang, Luo, et al., 2014). Apigenin is found to reduce the enzyme cyclooxygenase 2 (COX‐2) (Hassan et al., 2022) and therefore might be a useful remedy for inflammatory conditions in a mouse model that is prone to lupus. The inhibition of enzyme activity in CD4 + T cells, dendritic cells, B cells, and macrophages leads to the suppression of IFN‐γ and IL‐1 and IL‐17 mediated pro‐inflammatory cell hyperactivation. Astilbin, a different flavonoid, slows the progression of the inflammation in a mouse model of SLE by lowering serum levels of cytokines that are pro‐inflammatory including, IL‐1, TNF‐α, IL‐17A, IFN‐γ, IL‐6 along with the fraction of T and B cells that are activated (Shin et al., 2020). The role of the anti‐inflammatory action of phytochemicals in T2DM is demonstrated in Figure 6.

### 5.4 Anticancer activity

**FIGURE 7:**
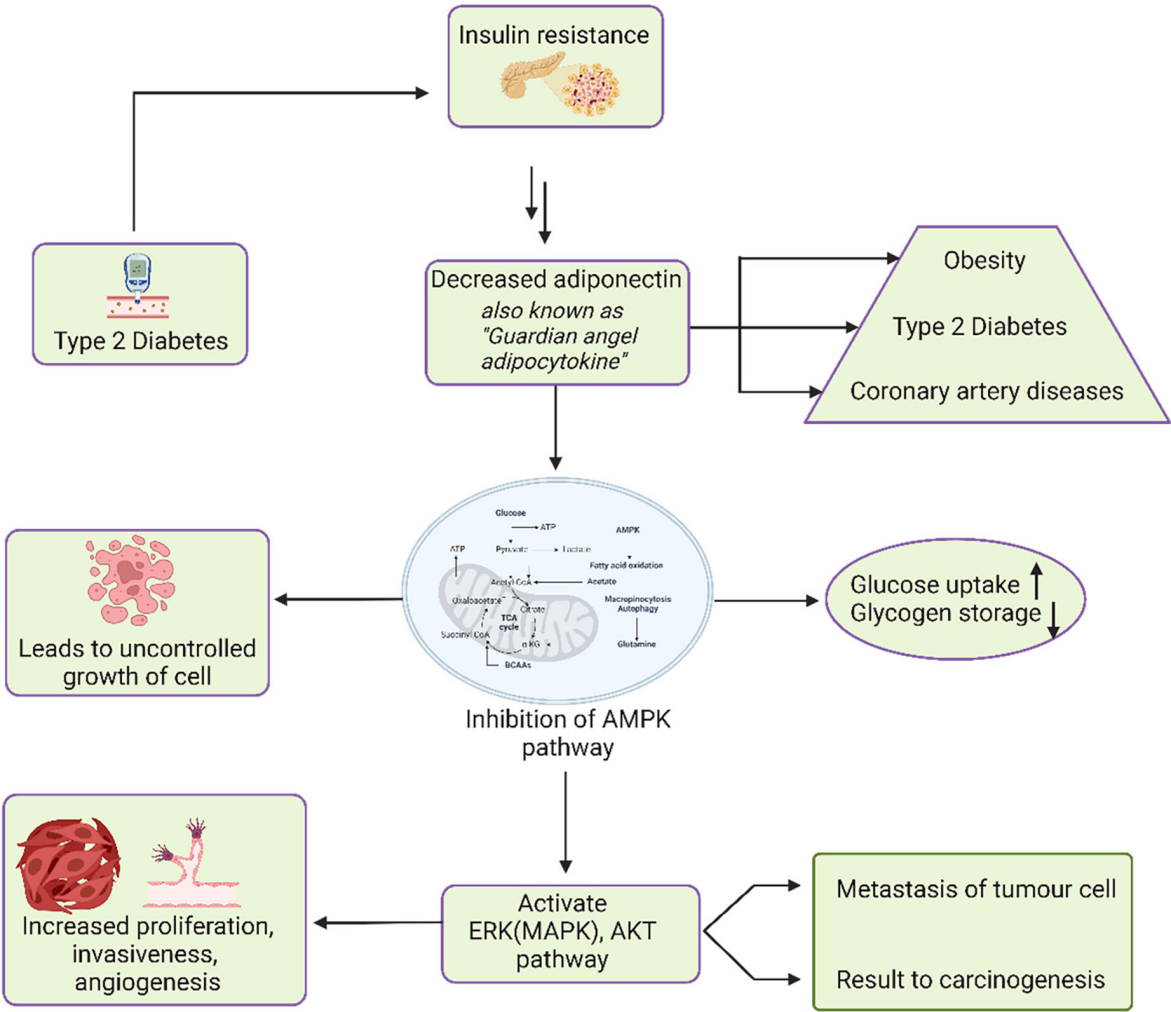
Mechanism of diabetes‐related anti‐cancer efficacy. T2DM contributes to the emergence of insulin resistance, thereby decreasing the adiponectin hormone, connecting with obesity and coronary artery disease, and inhibits the AMPK pathway. The inhibition of the AMPK pathway increases glucose uptake, which causes cells to expand out of control and also activates the ERK (MAPK) and AKT pathways, which in turn results in the metastasis of tumor cells leading to carcinogenesis.

A low‐grade chronic inflammatory condition is produced by hyperglycemia, hyperinsulinemia, and particularly central obesity and these factors show the correlation of DM to the onset and spread of cancer, which may also affect how well an anticancer treatment works (Calvo et al., 2010). Understanding the pathophysiologic relationships (Figure 6) between insulin, glycemia, obesity, and inflammation, and how they relate to cancer may help target DM treatment more successfully (Macciò & Madeddu, 2011). There is a known connection between T2DM and hepatocellular cancer as two of the key target organs for the metabolism of insulin are the liver and pancreas (Sun & Kashyap, 2011). In general, thyroid carcinoma is shown to be prevalent in patients with medication for type 2 diabetes (Dong et al., 2022). However, certain T2DM medications like metformin and rosiglitazone may decrease the prevalence of thyroid cancer (Seo et al., 2017). Insulin resistance, elevated insulin levels, and activation of the insulin receptor are associated with T2DM. Insulin resistance induces the phosphorylation of intracellular proteins, activating the extracellular signaling‐regulated kinase (ERK) chain, a type of mitogen‐activated protein kinase (MAPK) pathway, that promotes mitogenesis thus increases breast cancer risks (Ahmadieh & Azar, 2013). Diabetes is also linked to lower plasma levels of adiponectin, which blocks adenosine monophosphate‐activated protein kinase (AMPK) and activates the Akt (protein kinases B) and ERK paths, raising the risk of cancer (Sun & Kashyap, 2011). AMPK signaling reversibly modulates excessive MAPK signaling in cancer cells by phosphorylating its critical components, namely rapidly accelerated fibrosarcoma (RAF)/kinase suppress of RAS (KSR) family kinases (Yuan et al., 2020). In overweight patients with insulin resistance, elevated levels of circulation TNF‐α could promote carcinogenesis. TNF‐α has the potential to stimulate the growth of tumors by stimulating signaling pathways like MAPK and the antiapoptotic NFαB pathways (Arcidiacono et al., 2012). Women with T2DM who are resistant to insulin may have a drop in estrogen levels, which slightly increases their risk of malignancy in their breasts and may extend to additional organs with high levels of estrogen receptors, such as the endometrium and ovaries (Eketunde, 2020). Metformin, a drug that has been used to reduce blood sugar levels in individuals with T2DM by activating the AMPK pathway, has also been investigated in cancer treatment (Tebbe et al., 2014). The AMPK pathway inhibits abnormal cell growth, cell division, and angiogenesis by the mTOR signaling pathway and COX‐2 linked to the development of tumors. The consequences of T2DM and its correlation with cancer are demonstrated in Figure 7.

Several pieces of evidence showed that dietary polyphenols consist of anti‐tumor activities by targeting molecules involved in cell proliferation, survival, metastasis, as well as in angiogenesis (Bishayee & Sethi, 2016). Furthermore, they are also known to have immunomodulatory properties, potentially useful in immunotherapeutic approaches for treating cancer (Focaccetti et al., 2019). Among flavonoids, quercetin, and genistein have been shown to affect several molecular pathways involved in cell proliferation, and progression, including cell cycle, apoptosis, and oncogene expression, and consist of therapeutic effects in preclinical model colorectal cancer (Afrin et al., 2020). Furthermore, flavonoids and polysaccharides from okra flowers have been shown to possess the ability to attenuate induced colitis‐associated cancer through the regulation of NFκB/IL‐6/Stat3, JAK2/Stat3, MAPKs, PI3K/AKT, and Wnt/β‐catenin signal transductions in mice model (Deng et al., 2023). Phytochemicals like berberine, wogonin, resveratrol, magnolol, and curcumin have been found to suppress cancer growth by activating the AMPK pathway (Li et al., 2015). In comprehensive research on tea, polyphenols, like catechins were shown to slow the development and spread of tumors in cell culture (Davalli et al., 2012).

## 6 PHENOLIC COMPOUNDS FOR DIABETES COMPLICATIONS

The antihyperglycemic impacts of phytoconstituents are mostly brought about by their capacity to stimulate insulin secretion, limit intestinal glucose uptake, and promote the activity of metabolites in insulin‐dependent activities (Patel et al., 2012). To treat and prevent diabetes as well as its complications, researchers are prioritizing alternative therapeutic approaches, such as phytotherapeutics, with high antioxidant plant compounds and medicinal multi‐compound drugs that act on multiple targets for managing diabetes complications (Vennos et al., 2019). Numerous naturally occurring anti‐inflammatory polyphenols, including, stilbenes, flavonoids, and lignans can be further explored as innovative therapeutics for diabetes and its consequences (Kong et al., 2021).

Over half of people with diabetes experience diabetic peripheral neuropathy (DPN), which is connected with loss of sensation in the feet prompting trouble to walk and shortcoming in the foot muscles (Iqbal et al., 2018). Oxidative stress and inflammation are the key players of neuronal degeneration, leading to the insufficiency of neurochemical growth factors, neurovascular dysfunction, demyelination, and autoimmune damage of neurons (Schmidt, 2002; Vincent et al., 2002). As flavonoids are known to possess anti‐inflammatory properties, they were reported to act against DNP. Catechin was shown to reduce neuronal damage by increasing antioxidant enzymes and lymphocyte infiltration in nerve tissues (Addepalli & Suryavanshi, 2018). Similarly, deguelin is found to activate the NRF2 pathway and ameliorate diabetic neuropathy by regulating oxidative stress and neuroinflammation (Chen et al., 2018). It also partially restored the conduction velocities of neurons in diabetic rats by enhancing the activity of (Na + ‐K+) ATPase.

DM can result in a potentially blinding condition called diabetic retinopathy. The diabetic retina was found to have higher levels of pro‐inflammatory proteins such as TNF‐α, IL‐1 β, vascular endothelial growth factor (VEGF), and protein kinase C‐beta (PKC‐β) (Tang et al., 2023). Studies have demonstrated that the anti‐inflammatory, antioxidant, and anti‐angiogenic properties of *Moringa oleifera* are helpful against diabetic retinopathy (Kumar Gupta et al., 2013). As the extract reduced the overexpression of NF‐κB and vascular cell adhesion molecule (VCAM‐1) in the retina, it may be used to manage diabetic retinopathy by inhibiting angiogenic mediators (Kaur et al., 2023). Kaempferol at a concentration of 60 μmol/L has been found to enhance cell survival in retinal ganglion cells (RGCs) damaged by hyperglycemia by upregulating the expression of vascular inhibitors protein 1 and phosphorylating extracellular signal‐regulated kinase (Zhao et al., 2020).

A natural polyphenol called curcumin dramatically reduced retinal edema and performed better than insulin at preventing photoreceptor apoptosis (Xie, Chen, et al., 2021). Additionally, when combined with insulin, curcumin reduced the nuclear erythroid 2‐related compensatory factor 2 (Nrf2) pathway stimulation by lowering free radicals directly, and, in the early diabetes stage, it maintained Nrf2 pathway homeostasis better than insulin alone (Xie, Chen, et al., 2021). Hence, we suggest that phytotherapy can be a good approach to treatment and mitigating such diabetes complications.

It is a fact that DM increases the risk of cardiovascular morbidity and mortality. Macrovascular complications of diabetes include cardiovascular events, a disease of the coronary and peripheral arteries. It is associated with atherosclerotic plaque in the vessels that supply blood to the different organs, including the heart and brain. In later stages, macrovascular disease involves arterial blockage with an increased risk of myocardial infarction and stroke, leading to death (Barrett et al., 2017). Apigenin has been shown to protect T1DM in experimental models from cardiovascular damage by regulating oxidative and inflammatory mechanisms and preventing cardiomyocyte hypertrophy and fibrosis (Liu et al., 2017). Likewise, liquiritigenin, by inactivating the NF‐κB signaling pathway showed anti‐fibrotic and anti‐inflammatory properties and attenuated cardiac failure in the mice model of T2DM, Overall, it has been demonstrated that cardioprotective effects of medicinal herbs reduce damage to several types of cardiovascular system cells, including cardiomyocytes, macrophages, and monocytes, vascular smooth muscle cells, and endothelial cells. Antioxidant, anti‐inflammatory, anticoagulant, hypolipidemic, hypotensive, and diuretic actions can all have a ripple effect on the cardiovascular process (Ciumărnean et al., 2020). Diabetes kidney disease, commonly referred to as diabetic nephropathy (DN) is marked by diabetes‐related glomerular lesions, pathological levels of urine albumin excretion, and a decrease in the rate of glomerular filtration in diabetics (Lim, 2014). DN patients face a mortality risk that is almost 30 times higher than that of other diabetics who do not have DN (Sagoo & Gnudi, 2020). Quercetin, a flavonoid abundant in onions, has been linked to lowering blood pressure and visceral (belly) fat, along with increasing adipocyte death. Whereas genistein (Figure S1), a flavonoid abundant in soy products, is linked to a rise in high‐density lipoprotein (HDL) and glucose tolerance as well as a decrease in body fat mass (Kim & Park, 2020).

## 7 DISCUSSION

Throughout history and into the present, polyphenols have played a significant role in providing therapy for various diseases, including diabetes, in both developed and developing countries. Polyphenols are normally seen to be safe, when applied prescribed, as nutritional supplements, food additives, or medications (Mensah et al., 2019). Yet, there have been cases where both humans and animals have had negative side effects related to the use of herbal remedies (Mensah et al., 2019; Salleh et al., 2021), Nevertheless, there is increasingly more research demonstrating the effectiveness of using these phytochemicals in the therapeutic oversight of diabetes mellitus, although there are often no such reports about the toxic potential of phytochemicals in the aspect of DM depending upon intestinal absorption, toxicity, and metabolism in the liver (Singh et al., 2022). Although there are many readily available commercially antidiabetic medications, their positive effects are limited by their negative effects. Nutraceuticals and phytomedicines, in contrast, have a lower incidence of negative impact and can be excellent substitutes for conventional medications in the treatment of diabetes and its ramifications (Alam et al., 2022). According to the literature findings, polyphenols, such as kaempferol, catechins, apigenin, chlorogenic acid, and caffeic acid, are useful for treating diabetes and typically minimize the likelihood of getting diabetes by protecting the pancreatic islet beta cells, inviting its growth, and decreasing stress caused by oxidative damage and beta cell programmed cell death, along with lowering the intake of glucose in the intestine, hindering the functioning of digestive biocatalyst, modifying gene expression, and other mechanisms (Shahwan et al., 2022). Comprehensive analyses of the putative antidiabetic properties of polyphenols have been carried out in cell‐based investigations, research on humans, clinical trials, and animal models (Salahuddin et al., 2021). Alternative therapies such as polyphenols that can control diabetes more effectively and safely are being supported as a result of the limitation of the present oral antidiabetic medications' in terms of efficacy or safety, as well as the diseases rising as a global epidemic. *Galega officinalis* was needed to produce metformin, a less harmful biguanide, and powerful oral glucose‐lowering drug, which is used to treat diabetes (Fabricant & Farnsworth, 2001). In an experiment, the insulin secretagogues glibenclamide employed as the control drug, kaempferol promotes insulin secretion in a way similar to that of insulin secretagogues glibenclamide. It was discovered that kaempferol raised insulin levels in plasma and lowered blood sugar concentration in streptozotocin‐induced hyperglycemic rats (Al‐Numair et al., 2015). Additionally, several recent studies have demonstrated that kaempferol is a potential medication candidate and has good antidiabetes capabilities (Alkhalidy et al., 2018). Similarly, the other five phytochemicals from our concluded potent compounds also show similar type of properties. Existing research on phytochemicals has highlighted the immense potential in curing diabetes, but the research of actually incorporating phytochemicals as a medication after considering all the parameters including the toxicity profile is still a limitation, which is creating a barrier to utilizing the phytochemicals to their full extent. Furthermore, few clinical studies on the effects of polyphenols on diabetic patients are carried out and could provide important insights into how polyphenols affect the human body but such studies are still in early stages. When 10 mg of resveratrol, a non‐flavonoid compound, was given to diagnosed T2DM individuals who are not receiving insulin treatments, the results showed a decrease in the markers of oxidative stress, an increase in the glucose level in tissue, and the insulin signaling markers. However, no change was seen in the blood glucose, serum insulin, amylin, and lipid levels (Hausenblas et al., 2015). Hence, this type of study could highlight the potential of polyphenols, and the working mechanism of polyphenols in the human body. Therefore, further research should be focused on to fully understand the precise mechanism of action of particular polyphenols; more focus should be placed on the bioactive molecules that are produced from different medicinal plants since they may be able to treat diabetes. There are many sophisticated techniques, like microwave‐assisted extraction, ultrasonic‐assisted extraction, mass spectrometry for isolation, and analysis of phytochemicals from different medicinal plants. Also, the research on preclinical trials highlighting the effects of polyphenols on diabetic patients should be carried out.

## 8 CONCLUSION

Phytochemicals, particularly polyphenols, are one kind of diverse collection of molecules that are extremely important to humans as they have immense biological activities along with significant pharmaceutical characteristics. The key beneficial aspects of polyphenols are mainly due to their antioxidant and anti‐inflammatory properties, thereby reducing oxidative stress and acting as free radical scavengers. In addition to this, specifically dealing with diabetes, the relationship of the structure of the ring of phenols plays a critical role as it inhibits the activity of main enzymes, α‐amylase and α‐glucosidase by binding to their active sites through H‐bonding and resulting in the delay of hydrolysis of carbohydrates, consequently, lowering the blood glucose levels. The potential of polyphenols has been extensively studied through in vitro analysis, animal models, and a few preclinical trials as explained in this work and the findings demonstrated that dietary polyphenols can prevent or lessen the symptoms of diabetes and other vital diseases, including inflammation, obesity, and cancer. Based on the results of this review, natural polyphenols may be utilized to treat diabetes both preventively as well as curatively, and they may aid in the discovery of novel antidiabetic agents. Furthermore, this review has also portrayed the connection of diabetes with other vital diseases, including inflammation, obesity, and cancer. Polyphenols, namely, kaempferol, catechins, apigenin, chlorogenic acid, rosmarinic acid, and caffeic acid have shown their ability to serve as potential antidiabetic medicines and can be used as an alternative to the available antidiabetic medications as available antidiabetic medications show major side effects. Also, the use of polyphenols might have a few side effects although relatively less than available medications. Since preclinical studies can demonstrate the actual potential of polyphenols and the working mechanism of polyphenols in the human body, the preclinical studies are in their initial stage. Therefore, further research is required to elucidate the mechanism of action, improve dose and formulation, and assess long‐term safety and efficacy in clinical trials.

## AUTHOR CONTRIBUTIONS

**Dipa Aryal:** Formal analysis (equal); methodology (equal); writing – original draft (equal). **Soniya Joshi:** Formal analysis (equal); methodology (equal); writing – original draft (equal). **Nabin Kumar Thapa:** Methodology (equal); writing – original draft (equal). **Pratiksha Chaudhary:** Investigation (equal); validation (equal); writing – original draft (equal). **Sirjana Basaula:** Investigation (equal); resources (equal); writing – original draft (equal). **Usha Joshi:** Investigation (equal); methodology (equal); resources (equal); writing – original draft (equal). **Damodar Bhandari:** Investigation (equal); methodology (equal); writing – original draft (equal). **Hannah M. Rogers:** Resources (equal); visualization (equal). **Salyan Bhattarai:** Writing – review and editing (equal). **Khaga Raj Sharma:** Writing – review and editing (equal). **Bishnu P. Regmi:** Validation (equal); visualization (equal); writing – review and editing (equal). **Niranjan Parajuli:** Conceptualization (lead); formal analysis (equal); project administration (lead); supervision (lead); writing – review and editing (equal).

## FUNDING INFORMATION

This project was partly supported by the University Grants Commission, Nepal (Award No. CRIG‐78/79‐S&T‐01).

## CONFLICT OF INTEREST STATEMENT

There is no conflict of interest between authors.

## Supporting information


Appendix S1

## Data Availability

All necessary data are included in the manuscript and supplementary materials.
