# Supplementary material for: Dietary phenolic compounds as promising therapeutic agents for diabetes and its complications: A comprehensive review
Source: Food Sci Nutr. 2024 Jan 30;12(5):3025–45. doi: 10.1002/fsn3.3983 (PMC11077226; doi:10.1002/fsn3.3983)
Supplement: Supplementary file 1 — Appendix S1 [file FSN3-12-3025-s001.docx]

**Dietary Phenolic Compounds as Promising Therapeutic Agents for Diabetes and Its Complications: A Comprehensive Review**

Dipa Aryal^1,†^, Soniya Joshi^1,†^, Nabin Kumar Thapa^1^, Pratiksha Chaudhary^1^, Sirjana Basaula^1^, Usha Joshi^1^ , Damodar Bhandari^1^, Hannah M. Rogers^2^, Salyan Bhattarai^3^, Khaga Raj Sharma^1^, Bishnu P. Regmi^2^, and Niranjan Parajuli^1,*^

^1^Biological Chemistry Lab, Central Department of Chemistry, Tribhuvan University, Kritipur, 44618, Kathmandu, Nepal

^2^Department of Chemistry, Florida Agricultural and Mechanical University, Tallahassee, Florida 32307, USA

^3^Paraza Pharma, Inc., 2525 Avenue Marie-Curie, Montreal, QC H4S 2E1, Canada

**Table S1.** Plant-derived polyphenols with their antidiabetic properties and current research

| Classification | Compounds | Dietary and Plant sources | Food sources | Mechanism of action | Enzyme used | IC_50_ value | References |
| --- | --- | --- | --- | --- | --- | --- | --- |
| Flavonoids | Catechins | Arbutus unedo L. (root) (Strawberry), Green tea ( *Camellia sinesis*), cocoa, red wine | Strawberry root (Arbutus unedo L.,  Green teas *(Camellia sinesis)*, cocoa, red wine | Antioxidant activity, quench thirst, calm the mind, function as a diuretic, and be used to treat coughing, weariness, and light sleep, anti-inflammatory, detoxifying, and expectorant-like activities | ɑ-glucosidase | 87.55 ± 2.23 μg/mL | [(F. Islam et al., 2022; Mrabti et al., 2018; Silveira et al., 2019)](https://www.zotero.org/google-docs/?VRscN5) |
|  | Quercetin | *Polygonum aviculare* L. leaves Blueberries, apples, broccoli, beans, tea | Polygonum aviculare L. leaves Blueberries, apples, broccoli, beans, tea | Antioxidant activity, anti-inflammatory, and antidiabetic improve hyperglycemia and insulin resistance, underlying mode of action was linked to a decrease in the endoplasmic reticulum (ER) stress, oxidative stress, and β-cell death in the pancreas | dipeptidyl peptidase-IV (DPP-IV), *α*-glucosidase | 1.1507μg/mL(DPP-IV), 15.17 ± 3.25 μg/mL(*α*-glucosidase) | [(Cai et al., 2020; F. Islam et al., 2022; Silveira et al., 2019; Singh et al., 2020)](https://www.zotero.org/google-docs/?kmHpMS) |
|  | Betulin  (Terpenoid) | *Ruellia tuberosa,* Bark of birch tree | *Ruellia tuberosa,* Bark of birch tree | Reduced lipid levels in serum and tissues, improved insulin sensitivity, and alleviated diet-induced obesity. Additionally, betulin increased the stability and decreased the size of atherosclerotic plaques. | *α*- glucosidase | 23.2426 ± 1.4609μg/mL | [(Tang et al., 2011; B. Zhang, Xing, et al., 2017)](https://www.zotero.org/google-docs/?ZM7vQE) |
|  | Erucin | *Eruca vesicaria, Eruca sativa* Mill seeds, Seeds of salad rocket | *Eruca vesicaria, Eruca sativa* Mill seeds, Seeds of salad rocket | It Improves glycemic control. It acts as a painkiller, Anticancer, Antiinflammatory, Antidiabetic, Antibiotic, Antioxidant and also shows neuroprotective effects. | ɑ-amylase, ɑ-glucosidase | 0.80±0.06µg/mL | [(Lucarini et al., 2019)](https://www.zotero.org/google-docs/?pIMLhj) |
|  | Kaempferol | *Cucumis sativus* fruit ,Apples, onion, broccoli, tomato, green beans, citrus fruits, grapes, and ginkgo biloba | *Cucumis sativus fruit,* Apples, onion, broccoli, tomato, green beans, citrus fruits, grapes, and *ginkgo biloba* | Anti-diabetic activity by inhibiting the activity of a-amylase and a-glucosidase enzymes | ɑ-amylase, ɑ-glucosidase | 51.24µg/mL,  and 29.37µg/mL | [(Ibitoye et al., 2018; Lee et al., 2015)](https://www.zotero.org/google-docs/?TTNQxs) |
|  | Capsaicin  (Alkaloids) | *Capsicum chinense* fruits | *Capsicum chinense* fruits | Decrease in plasma glucose levels increases insulin levels, antioxidant activity, Enhancement of energy expenditure, increase in glucose absorption, Decrease of energy intake, and hunger | ɑ-amylase  α-glucosidase | 159 µg/mL,  117 µg/mL | [(Aryaeian et al., 2017; Leporini et al., 2019)](https://www.zotero.org/google-docs/?MHoePZ)  [(Aryaeian et al., 2017; Leporini et al., 2019)](https://www.zotero.org/google-docs/?TrmmyY)  [(Magaña-Barajas et al., 2021)](https://www.zotero.org/google-docs/?hSnA0Y) |
|  | Cinnamon acrolein | the inner bark of Cinnamomum tree(Cinnamon) | the inner bark of Cinnamomum tree(Cinnamon) | decreases fasting blood glucose levels, slows the rate of stomach emptying after eating, decreases the rise in blood sugar level | intestinal glucosidase and pancreatic amylase | 85µg/mL | [(Aryaeian et al., 2017; Sahib, 2016)](https://www.zotero.org/google-docs/?aGFYSG) |
|  | Baicalin | roots of *Scutellaria baicalensis* Georgi and the fruits of *Oroxylum indicum* | roots of *Scutellaria baicalensis* and the fruits of *Oroxylum indicum* | Antidiabetic effect, control hyperglycaemia and hyperlipidaemia in diabetic, antioxidant activity | α-glucosidase | 25.9± 0.412 µg/mL | [(Dewanjee et al., 2020; Lin et al., 2017; B. Zhang, Sang, et al., 2017)](https://www.zotero.org/google-docs/?79xGH7)  [(Fu et al., 2014)](https://www.zotero.org/google-docs/?5s35Vl) |
|  | Anthocyanin | Berberis integerrima fruits, Blueberries, Red cabbage, Cranberries | Berberis integerrima , Blueberries, Red cabbage, Cranberries | Increase the uptake and utilization of glucose by tissue, Improve insulin secretion, Lower the glucose level in the blood | α-amylase  α-glucosidase | 1140±o.3 µg/mL, 710± 85 µg/mL | [(Oliveira et al., 2020; Różańska & Regulska-Ilow, 2018)](https://www.zotero.org/google-docs/?BU5BKI) |
|  | Hesperetin | Lemon, Sweet orange | Lemon, Sweet orange | Strong antioxidant activity, Inhibit the formation of advanced glycatin end products. | α-glycosidase | 0.362736μg/mL | [(Revathy, 2017; Umeno et al., 2016)](https://www.zotero.org/google-docs/?V1P1C8) |
|  | Vitexin (apigenin-8-C-glucoside) | *Pennisetum millet* (L.) (Pearl millet), Mung bean, hawthorn, pigeon pea, chaste berry tea, mosses, Pearl millet,  *Phyllostachys edulis* Leaf | Phyllostachys edulis Leaf , Pennisetum millet (L.), Mung bean, hawthorn, pigeon pea, chaste berry tea, mosses, *Phyllostachys edulis* | formation of a stable complex between ɑ-glucosidase and vitexin resulting in the looser conformation of ɑ-glucosidase conformation and reduction of surface hydrophobicity ɑ-glucosidase inhibitor, ɑ-amylase inhibitor, adjustment of postprandial hyperglycemia | α-glucosidase | 22.829664μg/mL± 0.713427μg/mL | [(He et al., 2016; Ni et al., 2020; J.-P. Yang et al., 2014)](https://www.zotero.org/google-docs/?AH6fVB) |
|  | Taxifolin | *Rhizoma Smilacis Glabrae*, Red onion, Milk thistle, Acal palm, Pigmented rice, Orange, Grapefruit | *Rhizoma Smilacis Glabra,* Red onion, Milk thistle, Acal palm, Pigmented rice, Orange, Grapefruit | Antihyperglycemic effect, plays an important role in managing postprandial hyperglycemia. | α-glucosidase, α-amylase, pancreatic lipase | 38 µg/mL(α-glucosidase),  647µg/mL(α-amylase), 993µg/mL(pancreatic lipase  ) | [(Su et al., 2020)](https://www.zotero.org/google-docs/?nenVJz) |
|  | Chrysin | Oroxylum, Chamomille and Passiflora genus, Honey, Propolis, *Oroxylum indicum*, Passion flowers | Oroxylum, Chamomille and Passiflora genus,Honey, Propolis | Decrease the secretion of pro-inflammatory cytokines, Antidiabetic, Anti-inflammatory, and Antioxidant properties. | α -glycosidase | 0.20704 μg/mL | [(Ramírez-Espinosa et al., 2017; Taslimi et al., 2017)](https://www.zotero.org/google-docs/?8w3Mlt) |
|  | Daidzein | soybeans (Glycine max ) , other legumes, and Kudzu root (*Pueraria radix*) | soybeans (Glycine max), other legumes, and Kudzu root (*Pueraria radix*) | Increased insulin/ glucagon ratio, inhibits the rise in fasting blood sugar, increase in AMPK ( Adenosine monophosphate-activated protein kinase) phosphorylation, and subsequent GLUT4 (Glucose transporter 4) translocation to muscle cell plasma membrane, ɑ-glucosidase inhibition, ɑ-amylase inhibition | α-glucosidase, α-amylase | 12.10752μg/mL ± 0.50848μg/mL  76.526μg/mL± 7.805168 μg/mL | [(Alshehri et al., 2021; Cheong et al., 2014; Park et al., 2013)](https://www.zotero.org/google-docs/?PCnrF9) |
|  | Genistein | Soybeans (Glycine max ) red clover (Trifolium pratense), other legumes, and Kudzu root (*Pueraria radix*) | Soybeans (Glycine max) and red clover (Trifolium pratense) sother legumes, and Kudzu root (*Pueraria radix*) | Decrease in hepatic glucose production by maintaining insulin-positive 𝛃-cells and alters hepatic glucose metabolic enzyme profile | Tyrosine kinase | 2.16192μg/mL | [(Choi et al., 2008; Silveira et al., 2019)](https://www.zotero.org/google-docs/?ZXJUok)  [(Sharifi-Rad et al., 2021)](https://www.zotero.org/google-docs/?1mMSeA) [(Rimoldi et al., 2007)](https://www.zotero.org/google-docs/?IOqNAP) |
|  | Eriocitrin | Lemon  Peel, flavedo and juice vesicle of *Citrus lemon* | Lemon | Reduction of oxidative stress and inflammation, protects pancreatic islet cells, regeneration of β-cells, avoids the formation of advanced glycated end products, dipeptidyl peptidase (IV) inhibition | Dipeptidyl peptidase (IV) | 6.180 ± 0.054 μg /mL | [(Fan et al., 2013; Ferreira et al., 2021)](https://www.zotero.org/google-docs/?15tpPU) |
|  | Rosmarinic acid | Rosemary (*Rosmarinus officinalis*), Sage, Basil, Perilla, and Mint | Rosemary, Sage, Basil, Perilla, and Mint | lowers PECPK expression and elevates GLUT4 expression, lower hyperglycemia, and improves insulin sensitivity, anti-oxidant activity, ɑ-glucosidase inhibition | ɑ-glucosidase | 48.46 ± 0.52μg /mL | [(Inui et al., 2016)](https://www.zotero.org/google-docs/?dv8bDG) |
|  | Phloretin | Apple tree leaves (*Malus domestica. L* )and the Manchurian apricots*Fragaria x ananassa* | Apple tree leaves and the Manchurian apricots | Antioxidant, antidiabetic, anti-inflammatory, and antitumor activities | ɑ-glucosidase | 0.03126 µg/mL | [(Han et al., 2017; Tuli et al., 2022)](https://www.zotero.org/google-docs/?JpnYSx) |
|  | Myricetin | berries, fruits, (*Myrica rubra*) vegetables, honey, red wine, tea, and other daily foods | berries, fruits, vegetables, honey, red wine, tea, and other daily foods | Activation of GLP-1, Inhibition of α-Amy and α-Gls, Inhibition of CDK5, and . Inhibition of hIAPP aggregation | α-amylase, α-glucosidase,  DPP-4 activity | 662μg /mL, 3μg /mL, 1.528 ± 0.115 μg/mL | [(Meng et al., 2016; Song et al., 2021)](https://www.zotero.org/google-docs/?Hbk5ee) |
|  | Charantin | Bitter gourd  (*Momordica charantia*) | Bitter gourd | Responsible for hypoglycemic properties, Improve fasting glucose level | α-amylase | 16.17µg/mL | [(Ahmad et al., 2016; Kulkarni et al., 2021; B. Xu et al., 2022)](https://www.zotero.org/google-docs/?VlXNGw) |
|  | Isorhamnetin | Pears, Olive oil, tomato sauce, wine, Red onion | Pears, Olive oil, tomato sauce, wine, Red onion | attenuate diabetes complications, Maintain glucose levels | Rosiglitazone | 0.038 µg/mL | [(Kalai et al., 2022)](https://www.zotero.org/google-docs/?nWWvqf)  [(Y. Zhang et al., 2016)](https://www.zotero.org/google-docs/?27TDOA) |
|  | Rutin | Buckwheat, Citrus fruit, Unpeeled apples, Amaranth leaves | Buckwheat, Citrus fruit, Unpeeled apples, Amaranth leaves | Antioxidant, Anti-diabetic, Increases the flexibility in blood vessels, Increases insulin level | α-glucosidase, α-amylase | 7.86 µg/mL(α-glucosidase), 22.00 µg/mL(α-amylase) | [(Habtemariam & Lentini, 2015)](https://www.zotero.org/google-docs/?Jf0jFH) [(Maradesha, Patil, Phanindra, et al., 2022)](https://www.zotero.org/google-docs/?ZMc2MH) |
|  | Delphinidin | (*Melilotus officinalis* ) and Cranberries, Concord grapes, Pomegranates, bilberries | Cranberries, Concord grapes, Pomegranates, bilberries | Reduce the expression of cell adhesion molecules, avoid thrombosis, and act as an antioxidant, reduce glucose absorption, and increase insulin secretion | Aldose reductase, α-Glycosidase, α-Amylase | 8.095µg/mL(Aldose reductase), 0.0909µg/mL(α-Glycosidase), 0.204µg/mL(α-Amylase) | [(Lai et al., 2019)](https://www.zotero.org/google-docs/?i3tZ8v) [(Demir et al., 2019)](https://www.zotero.org/google-docs/?WlI1Nl) |
|  | Isoquercetin | Mango, Leaves of *Annona squamosa* ( Custard apple), and *Rheum nobile* | Mango, Leaves of *Annona squamosa* ( Custard apple), *Rheum nobile* | Improve the function of pancreatic islets, Regulate the blood glucose level | α-amylase | 9.65 ± 0.43µg/mL | [(Pleşca-Manea et al., 2002)](https://www.zotero.org/google-docs/?phjWii) [(Paun et al., 2020)](https://www.zotero.org/google-docs/?iygXCr) |
|  | Tangeretin | Oranges (*Citrus reticulata*), citrus peel of tangerine, and mandarins | Orange, citrus peel of tangerine, and mandarins | Reduction in blood glucose, total cholesterol, and body weight and regulates adipocytokines, leptin IL-6, and adiponectin | PPL-mediated 4-MU oleate hydrolysis | 5.470 ± 0.307𝜇g/mL | [(Cirmi et al., 2016)](https://www.zotero.org/google-docs/?niFetj) [(Kim et al., 2012)](https://www.zotero.org/google-docs/?Mzi4rJ) |
|  | Fisetin | Apples, grapes, persimmon cucumber, onion, and strawberries | Apples, grapes, persimmon cucumber, onion, and strawberries | Show antidiabetic, anti-inflammatory, and neurotrophic effects | ɑ-glucosidase | 9.38 ± 0.35𝜇g/mL | [(Arai et al., 2000)](https://www.zotero.org/google-docs/?r2AvHV) [(Kim et al., 2012)](https://www.zotero.org/google-docs/?9KvtC0) |
|  | Luteolin | Peppers, parsley, spices, broccoli, celery, onion leaves, carrots, cabbage, apple skin, pumpkins, and red leaf lettuce | Peppers, parsley, spices, broccoli, celery, onion leaves, carrots, cabbage, apple skin, pumpkins, and red leaf lettuce | Strengthen the immune system, relieve inflammation, cure combat cancer, Antioxidant activity, Facilitate the secretion of insulin, Hypoglycemic ability | dipeptidyl peptidase-IV (DPP-IV) | 0.0343 ± 0.0028 *μ*g/mL | [(Sangeetha, 2019)](https://www.zotero.org/google-docs/?drlQI1)  [(Silveira et al., 2019)](https://www.zotero.org/google-docs/?0hPGQH)  [(F. Islam et al., 2022)](https://www.zotero.org/google-docs/?tiM3QY)  [(Fan et al., 2013)](https://www.zotero.org/google-docs/?j9E3Dx) |
|  | Apigenin | Celery, Chamomile tea, parsley, Celeriac | Celery, Chamomile tea, parsley, Celeriac | Antiinflammatory, Antioxidant, Antibacterial, Antiviral activities, Blood pressure reduction, Stimulate the metabolism of glucose, Enhance secretion of insulin from the pancreas, Ability to overcome dyslipidemia | ɑ-glucosidase | 2.838 ± 0.014  *μ*g/mL | [(*The Potential Role of Apigenin in Diabetes Mellitus \| Auctores*, n.d.)](https://www.zotero.org/google-docs/?wFyTPW)  [(Zeng et al., 2016)](https://www.zotero.org/google-docs/?btc2TX) |
|  | Eriodictyol | Citrus fruits, Vegetables, Chinese herbs (*Artemisiae argyi, Aspalathus linearis*) | Citrus fruits, Vegetables, Chinese herbs | Antioxidant, Antiinflammatory, Antidiabetic, Antiobesity, Hepatoprotective properties | Pancreatic lipase, ɑ-glucosidase | 38.62 ± 5.59 µg/mL  16.57 ± 3.79 µg/mL | [(A. Islam et al., 2020)](https://www.zotero.org/google-docs/?6GjSyW)  [(Habtemariam, 2012)](https://www.zotero.org/google-docs/?Oz1fLY) |
|  | Naringenin | Citrus fruits like grapefruit (*Citrus paradisi*), pomelo (*Citrus grandis*), sour orange (*Citrus aurantium*) Cocoa, cherries | Grapefruit, Oranges, Tomatoes, citrus fruit, Cocoa, cherries | Antioxidant, Antiinflammatory, Reduces oxidative damage to DNA, Use to treat high blood pressure, Used for cholesterol control, Increases cell viability, defe nds trophoblasts and endothelial cells against a hazardous environment of excess glucose | ɑ-glucosidase | 104.54 µg/mL | [(Da Pozzo et al., 2017)](https://www.zotero.org/google-docs/?6MJ4Yk)  [(Zhao et al., 2020)](https://www.zotero.org/google-docs/?IhXSx1)  [(Priscilla et al., 2014)](https://www.zotero.org/google-docs/?9CPLJ0) |
|  | Coumestrol | Soybean (*Glycine max*), Brussels sprout (*Brassica oleracea*), spinach (*Spinacia oleracea*) | Flax seeds, Soybeans, Nuts, sunflower seeds, Sesame seeds, | Beneficial in combating symptoms and condition caused by estrogen deficiency, Antiobesity, Antidiabetic, Improve glucose control and insulin resistance | ɑ-glucosidase | 6.0 ± 0.6µM | [(Bhathena & Velasquez, 2002)](https://www.zotero.org/google-docs/?ME2HxP) [(Yuk et al., 2011)](https://www.zotero.org/google-docs/?jkFHCj) |
|  | Glycitein | Soybean (*Glycine max*), soy products like tofu, soy milk, soy sauce, sprouts, natto, edamame | Soy products like tofu, soy milk, soy sauce, sprouts, natto, edamame | Boost insulin's capacity to remove glucose from the blood, helps to prevent or manage diabetes, Helps to improve insomnia, elevate mood, Enhance cognition and memory | Xanthine oxidase | 12 ± 0.86 µg/mL | [(Minocha et al., 2022)](https://www.zotero.org/google-docs/?44K5DV) |
|  | Delphinidin 3-rutinoside | Blackcurrant berries (*Ribes nigrum L*.), & eggplants (*Solanum melongena*) | Blackcurrant berries, & eggplants | Antidiabetic activity by stimulating GLP-1 secretion in GLUTag cells through increased Ca^2+^-CaMKII pathway activation. |  |  | [(Kato et al., 2015; Miladinovic et al., 2023; Todaro et al., 2009)](https://www.zotero.org/google-docs/?7Kdbbq) |
|  | Delphinidin-3-glucoside | Blue corn (*Zea mays L*.), black bean (*Phaseolus vulgaris L*.), blueberry wine lees | Blue corn, black bean, blueberry wine lees | shows potential for binding with 11𝛃-HS1 ( 11β-hydroxy steroid dehydrogenase-1), increases insulin sensitivity, GFAT inhibition, protects pancreatic 𝛃-cell | ɑ-glucosidase | 364.32 ± 15.19 µM | [(Damián-Medina et al., 2020; Promyos et al., 2020; Wu et al., 2017)](https://www.zotero.org/google-docs/?cQ8Hzi) |
|  | Rhoifolin | Bitter orange, bergamot, grapefruit, lemon, Lupinus, lablab beans, tomatoes, artichoke, bananas, and grapes | Bitter orange, bergamot, grapefruit, lemon, Lupinus, lablab beans, tomatoes, artichoke, bananas, and grapes | Antidiabetic activity by Enhancing adiponectin secretion, tyrosine phosphorylation of insulin receptor-β and GLUT4 translocation |  | 22.6 µg/ mL | [(Eldahshan, 2013; “[No Title Found],” n.d.)](https://www.zotero.org/google-docs/?qgacF1) |
|  | Epigallocatechin gallate | Green tea (*Camellia sinensis*) Cranberries, strawberries, blackberries, cherries, pears, pistachios | Cranberries, strawberries, blackberries, cherries, pears, green tea, pistachios | antioxidant, anti-inflammatory, anti collagenase, anti-fibrosis, Osteogenesis promotion, dipeptidyl peptidase (IV) inhibition | dipeptidyl peptidase (IV) | 10.21± 0.75  µM | [(Chu et al., 2017; Fan et al., 2013; Harnly et al., 2006)](https://www.zotero.org/google-docs/?tI0yqv) |
|  | Cyanidin-3-O-glucoside | Black rice, couch grass, Mulberry, blueberry, bilberry, grape | Black rice, couch grass, Mulberry, blueberry, bilberry, grape | Decreases blood glucose and serum insulin, Inhibition of ɑ-glucosidase and dipeptidyl peptidase 4, antioxidant, anti-inflammatory | ɑ-glucosidase | 479.8 µM (238µg/mL) | [(Cásedas et al., 2019)](https://www.zotero.org/google-docs/?cRcQTo)  [(*Cyanidin-3-Glucoside from Black Rice Ameliorates Diabetic Nephropathy via Reducing Blood Glucose, Suppressing Oxidative Stress and Inflammation, and Regulating Transforming Growth Factor Β1/Smad Expression \| Journal of Agricultural and Food Chemistry*, n.d.)](https://www.zotero.org/google-docs/?hpnRbG) |
|  | Morin hydrate | white mulberry, (*Morus alba L*.), almond (*Prunus dulcis*), sweet chestnut (*Castanea sativa*) almond, sweet chestnut | white mulberry, almond, sweet chestnut | Antidiabetic activity by inhibition of IAPP (Islet Amyloid Polypeptide) amyloid formation and disaggregating IAPP amyloid fibers. | N-methylpurine DNA glycosylase | 2.6 µM | [(Noor et al., 2012)](https://www.zotero.org/google-docs/?nQFVB1)  [(Venu Gopal, 2013)](https://www.zotero.org/google-docs/?8JXe0x)  [(Dixon et al., 2015)](https://www.zotero.org/google-docs/?8VNK2q) |
|  | Silymarin | Seed of milk thistle, (*Silybum marianum*)  *Lycium chinense, Saururus chinesis* | Seed of milk thistle, *Lycium chinense, Saururus chinesis* | Antioxidant, Antiinflammatory, Protect various tissue and organ against various chemical injury, Enhance blood sugar regulation and stop the progression of the problem, restore antioxidant enzymes, decreases pancreatic TNF-ɑ(tumor necrosis factor-ɑ), decrease inflammation, oxidative stress, and serum amylase, showed radical scavenging activity | ɑ-amylase | 113.5 µg/mL(DPPH assay) | [(Voroneanu et al., 2016)](https://www.zotero.org/google-docs/?beYIRn)  [(Choo et al., 2010)](https://www.zotero.org/google-docs/?J2tQcp)  [(Stolf et al., 2018)](https://www.zotero.org/google-docs/?JoLDH7) |
|  | Biochanin A | *Trifolium pratense, Cicer arietinum, Lupinus termis*, and other legume plants | *Trifolium pratense, Cicer arietinum, Lupinus termis*, and other legume plants | Reduces glucose tolerance, decreased the formation of Glycohemoglobin, decreases insulin resistance, and improves insulin sensitivity, lipid profile, and ɑ-glucosidase inhibition | ɑ-glucosidase | 5.685µg/mL | [(Oza & Kulkarni, 2018)](https://www.zotero.org/google-docs/?KKKcnS)  [(Jiang et al., 2015)](https://www.zotero.org/google-docs/?aEP2IJ) |
|  | Arbutin | *Pyrus biossieriana Buhse*, Mulberry( *Morus alba)* | *Pyrus biossieriana Buhse*, *Morus alba* (Mulberry) | Increases expression and activity of superoxide dismutase, Catalase, glutathione peroxide, inhibits the rise in blood glucose, increased plasma insulin levels,ɑ-amylase and, ɑ-glucosidase inhibition, hypoglycemic activities, protects HK-2 cells, tyrosinase inhibitor | Tyrosinase | 3.55µg/mL | [(H. Li et al., 2021)](https://www.zotero.org/google-docs/?0Leumf)  [(Lv et al., 2019)](https://www.zotero.org/google-docs/?zYQxmi)  [(Yousefi et al., 2013)](https://www.zotero.org/google-docs/?rDDASP)  [(Jeon & Choi, 2019)](https://www.zotero.org/google-docs/?9wQBe0) |
|  | Rhamnetin | Cloves (*Syzygium aromaticum*), Spireae, *Acrytophyllum thymifolium* | Cloves, Spireae, *Acrytophyllum thymifolium* | Anticancer, Antioxidant, Antiinflammatory, Antiviral, Antibacterial activity | ɑ-amylase | 23.36µg/mL | [(Olennikov & Chirikova, 2018)](https://www.zotero.org/google-docs/?MPjyoR)  [(Milella et al., 2016)](https://www.zotero.org/google-docs/?R0LQS6) |
|  | Plumieride | *Plumeria inodora,* bark of *Plumeria bicolor, Plumeria alba, Allamanda blanchetii*  *Himatanthus drasticus* | *Plumeria inodora,* Bark of *Plumeria bicolor, Plumeria alba, Allamanda blanchetii* | Antioxidant, Antiinflammatory, Anti diabetic, Reduces the amount of colonic lipid hydroperoxide | *α*-amylase | 33.87 μg/mL | [(Rahman, 2014)](https://www.zotero.org/google-docs/?dgzWqB)  [(Rahman, 2014)](https://www.zotero.org/google-docs/?KleHde)  [(Boeing et al., 2018)](https://www.zotero.org/google-docs/?2RIDLL)  [(Morais et al., 2020)](https://www.zotero.org/google-docs/?XlPItA) |
|  | Scutellarin | *Scutellaria barbata*,  *S. lateriflora* | *Scutellaria barbata*,  *S. lateriflora* | Antidiabetic activity, antioxidant activity, reduce hyperglycemia | α-Glucosidase,  α-Amylase | 313.25 ± 7.28µg/mL,  369.52 ± 8.43µg/mL | [(K. Li et al., 2018)](https://www.zotero.org/google-docs/?xAltXB) |
|  | Proanthocyanidin | *Hordeum vulgare* (Barley), *Zea mays*(maize), Frageria x ananassa(strawberries), *Theobroma cacao* (cocoa), *Prunus amygdalus* (almond), *Cinnamomum zeylanicum* (cinnamon), *Arachis hypogaea*(peanuts) and *Camellia sinensis*(tea). | Barley, hops, maize, apples, grapes, strawberries, cocoa, almonds, cinnamon, peanuts, and tea | Antioxidant, Antiinflammatory, Antifungal, Anticancer, Protection against renal insufficiency, Maintains the glucose level, Antidiabetic | α-Glucosidase | (0.23 ± 0.01) μg m/L | [(Nie & Stürzenbaum, 2019)](https://www.zotero.org/google-docs/?beOSCC)  [(Han et al., 2018)](https://www.zotero.org/google-docs/?9iSfz5)  [(Yokozawa et al., 2012)](https://www.zotero.org/google-docs/?MJYGi5) |
|  | Wedelolactone | *Wedelia calendulacea* | *Wedelia calendulacea* | Inhibition of α-Glucosidase and  α-Amylase, showed antihyperlipidemic effect, anti-oxidant, downregulation of oxidative stress | α-glucosidase, rat-intestinal sucrase, rat-intestinal maltase | 14.643µg/mL,  6.9762µg/mL,  6.3477µg/mL | [(V. Kumar et al., 2018)](https://www.zotero.org/google-docs/?UErP3G)  [(*Chemistry & Biology Interface \| January-February 2012, Volume 2, No.1*, n.d.)](https://www.zotero.org/google-docs/?0cd8yI) |
|  | Wogonin | Root of *Scutellaria baicalensis* | *Scutellaria baicalensis* | Improves glucose tolerance, lowers serum insulin and cholesterol levels, reduction of lipid droplets and glycogen buildup in the liver, improves PPARα and adinopectin expression through AMPK activation | α-Glucosidase | 22.18 ±0.841µg/mL | [(J.-R. Yang et al., 2015)](https://www.zotero.org/google-docs/?7di4F9) |
| Non-flavonoids | Resveratrol | *Rubus idaeus, Arachis hypagaes, Morus alba, Morus nigre, Vitis vinifera* | Grapes, berries, red wine, grains, fruits, vegetables, dry legumes, and plant-derived beverages such as tea, coffee, and wine | Reduce hyperglycemia and improvements in insulin sensitivity in prediabetic patients, Antifungal activity, Neuroprotective effect, Antioxidant activity, Antiobesity, antidiabetes, Anticancer, Anti-inflammatory, Antioxidative, and Cardiovascular activities | ɑ-amylase, α-Glucosidase, rat lens AR,  rat kidney AR, | 3.62µg/mL, 17.54µg/mL, 4.99 μg/ml (against rat lens AR) and 5.49 μg/ml (rat kidney AR) | [(Silveira et al., 2019)](https://www.zotero.org/google-docs/?fVtTwe)  [(Lin et al., 2017)](https://www.zotero.org/google-docs/?bj75TQ)  [(Shen et al., 2017)](https://www.zotero.org/google-docs/?qxfocg)  [(Ciddi & Dodda, 2014)](https://www.zotero.org/google-docs/?1v9PeV) |
|  | Peniocerol | *Peniocereus greggii*( Queen of the night) | *Peniocereus greggii*( Queen of the night) | Hypoglycemic and antihyperglycemic action for treating diabetes, used for the treatment of breast pain, and the common cold. |  | 9.958±1.5705µg/mL | [(Muñoz-Gómez et al., 2022)](https://www.zotero.org/google-docs/?un3J2C)  [(Salazar et al., 2011)](https://www.zotero.org/google-docs/?4s7KbP) |
|  | Viperidone | Whole grains, seeds, nuts, beans | Whole grains, seeds, nuts, beans | Improve blood sugar control, Insulin sensitizer |  |  | [(Muñoz-Gómez et al., 2022)](https://www.zotero.org/google-docs/?QkfgMv) |
|  |  |  |  |  |  |  |  |
|  | Curcumin | Root of *Curcuma Longa*, *Berberis aristata, Lagerstroemia speciose* | Root of *Curcuma Longa* and Indian curry spice | Antioxidant, anti-inflammatory and anticancer activity, reduce serum glucose and lipid levels, improve liver and kidney function, improve glucose, and lipid homeostasis | ɑ-amylase | 18.9052µg/mL | [(Aryaeian et al., 2017)](https://www.zotero.org/google-docs/?EFpjhd)  [(Den Hartogh et al., 2020)](https://www.zotero.org/google-docs/?BX4Pxp)  [(Uğur et al., 2022)](https://www.zotero.org/google-docs/?CR0Vgx) |
|  | Mangiferin | mango tree (*Mangifera indica*), *Iris unguicularis, Anemarrhena asphodeloides* | mango tree (*Mangifera indica*), *Iris unguicularis, Anemarrhena asphodeloides* | Improves oral glucose tolerance, reduces fasting plasma glucose level, inhibits the activity of a-amylase, and a-glycosidase | α- amylase,  α- glucosidase | 63.57 μg/ml ( against α- amylase)  36.84 μg/ml (against α- glucosidase) | [(Alam, Sarker, et al., 2022)](https://www.zotero.org/google-docs/?ueK7Kk)  [(Sekar et al., 2019)](https://www.zotero.org/google-docs/?8VnIvJ) |
|  | Ferulic acid | *Solanum lycopersium (*tomato),*Raphanus sativus* ( radish), *Brassica oleracea var. italic* (broccoli),  *Persea americana (*avocado), *Musa acuminata (*banana), *Solanum melongena* (eggplant), *Spinacia oleracea (*spinach). | commelinid plants (rice, wheat, oats, and pineapple), grasses, grains, vegetables, flowers, fruits, leaves, beans, seeds of coffee, artichoke, peanut, and nuts | exhibits a wide range of biological properties including metal chelation, modulation of enzyme activity, activation of transcriptional factors, gene expression, and signal transduction. It is also anti-inflammatory, anti-microbial, anti-allergic, hepatoprotective, anticarcinogenic, antithrombotic, and increases sperm viability | α- amylase,  α- glucosidase | 622 μg/ml  866 μg/ml | [(N. Kumar & Pruthi, 2014)](https://www.zotero.org/google-docs/?RujKjX)  [(Soobrattee et al., 2005)](https://www.zotero.org/google-docs/?nikCSA)  [(Sri Balasubashini et al., 2003)](https://www.zotero.org/google-docs/?cif3H1)  [(Zheng et al., 2020)](https://www.zotero.org/google-docs/?GmmQU5) |
|  | Anthraquinone | extracts of *Rheum rhabarbarum* (rhubarb) *Rhamnus cathartic (*buckthorn), and other herbal products such as roots, bark, or dried leaves of*Senna, cascara* and *Frangula alnus.* | extracts of rhubarb, aloe, or buckthorn, and other herbal products such as roots, bark, or dried leaves of senna, cascara, frangula | antioxidant activity, antidiabetic, antiviral, antibacterial, antimicrobial, antiosteoporotic, immunosuppressive, neuroprotective, hepatoprotective, anticancer, anti-inflammatory, antiatherosclerotic, antiallergic, antiulcerogenic, cathartic, diuretic, laxative activities, DNA binding, and vasorelaxant activities | α-glucosidase | 28.94 μg/ml | [(Martorell et al., 2021)](https://www.zotero.org/google-docs/?ucqeQK) [(Zarren et al., 2021)](https://www.zotero.org/google-docs/?zIQk2H) |
|  | Coumarin | *Dipteryx odorata* (Tonka beans), Cinnamon, peppermint, melilotus (sweet clover), honey. *Vaccinium myrtillus* (bilberry) | Tonka beans, peanuts corns, nuts, peppermint, melilot | Marked antihyperlipidemic effect against DM, Shows lipid-lowering effect, Reduces nephrotoxicity | α-Glucosidase | 1.10 ± 0.01µM to  36.46 ± 0.70µM | [(Pari et al., 2014)](https://www.zotero.org/google-docs/?xLgpRX)  [(Taha et al., 2018)](https://www.zotero.org/google-docs/?4bYltf) |
|  | Caffeic acid | *Olea europea* (Olives), *Coffea arabica, Coffea canephora* (coffee beans), *Artocarpus heterophyllus*,, *Daucus carota* (carrot), and propolis (bee glue). | Olives, coffee beans, fruits, potatoes, carrots, and propolis. | Antioxidant activity, anti-inflammatory activity, cardioprotective activity, antidiabetic activity, and anticancer activity. | α-amylase, α-Glucosidase, Aldose reductase | 26.90 ± 0.05µg/mL, 8.00 ± 0.40µg/mL,3.10 ± 0.33µg | [(Espíndola et al., 2019)](https://www.zotero.org/google-docs/?AZYvu4)  [(Maradesha, Patil, Al-Mutairi, et al., 2022)](https://www.zotero.org/google-docs/?t0nrlL) |
|  | Ellagic acid | , *Quercus mongolica, Crataegus azarolus* (leaves of maire)*, Vaccinium oxycoccos (*cranberry), *Vitis rotundifolia* (Muscadine), Flower of Pomegranate | Strawberries, Raspberries, Walnuts, and Berries | Stimulate insulin production, Reduce glucose to improve diabetic complications, and Show Anticancer and Antioxidant activity. | α-amylase, α-Glucosidase | 3.7743µg/mL, 2.18µg/mL | [(Amor et al., 2020)](https://www.zotero.org/google-docs/?BvrvTg)  [(Mehta et al., 2017)](https://www.zotero.org/google-docs/?jWLDm0) [(You et al., 2012)](https://www.zotero.org/google-docs/?G8BsCn) |
|  | Punicalagin | *Punica granatum linn (*Pomegranate) | Pomegranate | Reduce oxidative stress, Induce metal chelation activity, Decreases fasting blood glucose level | α-glucosidase | 285.21 ± 1.9 g/mL | [(Banihani et al., 2013)](https://www.zotero.org/google-docs/?PORbfJ) [(Alam, Dhar, et al., 2022)](https://www.zotero.org/google-docs/?V80Igw) |
|  | Gallic acid | , *Hibiscus sabdarifia* , *Anacardium humile, Terminalia chebula, Sanguisorba officinalis* great burnet), *Syzygium cumini* (Witch hazel) | Gallnuts, tea leaves, oak bark, Witch hazel | Immunity regulator against infections, Proper secretion of insulin, Antioxidant activity | α- amylase,  α- glucosidase | 20.12  μg/ml,  22.29μg/ml | [(Y. Xu et al., 2021)](https://www.zotero.org/google-docs/?J784ac) [(Alegbe et al., 2019)](https://www.zotero.org/google-docs/?oFxXSA) |
|  | Stilbenes | *Arachis hypogaea (*peanut*)*,  Red Sorghum  *Peniocereus greggi* | Blueberries, dry fruits, whole grain products | Improve insulin resistance, Hinder glucose uptake in tissue | α-glucosidase | 13.57 and 14.39μM for trimer and tetramer  2.445 µg/mL,  and 2.593 µg/mL | [(Shazmeen et al., 2021)](https://www.zotero.org/google-docs/?N7F80E) [(Muñoz-Gómez et al., 2022)](https://www.zotero.org/google-docs/?oxdJb5) |
|  |  |  |  |  |  |  |  |
|  | Sinapic acids | *Vaccinium , Triticum aestivum* sprouts | Blueberries, kiwis, plums, cherries, apples | Anti-inflammatory action through the decrease in the expression of pro-inflammatory cytokines | α-amylase α-glucosidase | 301±2.7 µg/mL | [(Sri Balasubashini et al., 2003)](https://www.zotero.org/google-docs/?FEEZv6)  [(Yun et al., 2008)](https://www.zotero.org/google-docs/?bP12S2)  [(Jeong et al., 2012)](https://www.zotero.org/google-docs/?vsZ3GB) |
|  | Chicoric acid | *Echinacea purpurea*, *Pterocypsela laciniata,*and *Cichorium intybus* | Echinacea purpurea, Pterocypsela laciniata, Orthosiphon stamineus, and Cichorium intybus | Increase insulin secretion in a glucose-dependent manner in Langerhans islets | *α*-glucosidase | 0.28 mg/mL  280 µg/mL | [(*Chicoric Acid, a New Compound Able to Enhance Insulin Release and Glucose Uptake - ScienceDirect*, n.d.)](https://www.zotero.org/google-docs/?w5GrHF)  [(M. Yang et al., 2022)](https://www.zotero.org/google-docs/?d2s8sv) [(*Antioxidant, Antidiabetic, and Antihypertension Inhibitory Potentials of Phenolic Rich Medicinal Plants*, n.d.)](https://www.zotero.org/google-docs/?KtDr6K) |
|  | Syringic acid | Fruits like  *Artocarpus heterophyllus* L and Christmas berry, sugar apple, grapes, olive, Date palm, marigold, radish, honey | Downy oak, Christmas berry, sugar apple, grapes, olive, Date palm, marigold, radish, honey | Enhances Na/K ATPase activity, decreases hyperglycemia, polydipsia, relative organ weight, inflammatory indicators, glycated haemoglobin, oxidative stress, ɑ-amylase inhibition, ɑ-glucosidase inhibition, aldose reductase inhibition | α-amylase, α-Glucosidase, Aldose reductase | 25.25 ± 1.00 µg/mL,  7.50±1.05µg/mL,  3.00 ±o.ooµg/mL | [(Mirza et al., 2022)](https://www.zotero.org/google-docs/?hyXb86)  [(Srinivasulu et al., 2018)](https://www.zotero.org/google-docs/?EmRhxb)  [(Maradesha, Patil, Al-Mutairi, et al., 2022)](https://www.zotero.org/google-docs/?rqUUek) |
|  | Vanillic acid | *Solanum tuberosum*, *Euterpe oleracea*, orchard grass, banana, mango, *Angelica sinensis* | Olives, cereal grains, wine, beer, cider, *Solanum tuberosum*, *Euterpe oleracea*, orchard grass, banana, mango, *Angelica sinensis* | Reduction in blood sugar, insulin, and blood pressure  Activation of tissue antioxidants to fight oxidative damage, a significant increase in activities of enzyme antioxidants | α-amylase | 0.35 ± 0.06mg/mL  350 ± 60 µg/mL | [(Vinothiya & Ashokkumar, 2017)](https://www.zotero.org/google-docs/?oG6GHb)  [(Kiokias et al., 2020)](https://www.zotero.org/google-docs/?xMeFxM) [(Taqvi et al., 2021)](https://www.zotero.org/google-docs/?Xiz0LZ) [(Aleixandre et al., 2022)](https://www.zotero.org/google-docs/?Yw80fK) |
|  | Secoisolariciresinol | Seeds like Linseed( *Linum usitatissimum*,), and sesame, pumpkin seeds, rye, sunflower | Linseed, cereals, grain, sesame, pumpkin seeds, rye, sunflower | Prevent some malignancies, Anticancer, Antiestrogenic, Antiproliferative, Antiangiogenic activities, Reduction of glycated haemoglobin | α-glucosidase | 1000 µg/mL | [(Prasad, 2000)](https://www.zotero.org/google-docs/?RldDEE) [(*Glucose Uptake and α-Glycosidase Inhibition Activities of Secoisolariciresinol Diglucoside Isolated from Linum Usitatissimum*, n.d.)](https://www.zotero.org/google-docs/?lZauFa) |
|  | Daucosterol | *Fallopia cillinerve root , Dendrbium huoshanense stem ,* and  *Denrobium officinale* | *Mitragyna speciosa, Rheum turkestanicum* | antioxidant, diabetic-friendly, lipid-lowering, anti-inflammatory, immune-modulating, neuroprotective, and anticancer | α-glucosidase | 247.35 mg/L  247.35 µg/mL | [(El Omari et al., 2022)](https://www.zotero.org/google-docs/?pbffm3) |
|  | Protocatechuic acid | , Leaves of *Diospyros melanoxylon, Hibiscus sabdariffa, Allium cepa* | Green tea, Stem bark of Boswellia, Leaves of *Diospyros melanoxylon, Hibiscus sabdariffa, Allium cepa* | Antioxidant, Antiinflammatory, Antifungal, Anticancer, Increase endogenous resistance | α- amylase,  α- glucosidase | 27.03μg/ml, 24.30μg/mL | [(*A Review on Protocatechuic Acid and Its Pharmacological Potential*, n.d.)](https://www.zotero.org/google-docs/?P2Pw4m) [(Semaming et al., 2015)](https://www.zotero.org/google-docs/?BFzZDk) |
|  | Chlorogenic acid | Apples, pears, carrots, tomatoes, sweet potatoes, eggplant, coffee, tea, blueberries, and sunflower seeds | Apples, pears, carrots, tomatoes, sweet potatoes, eggplant, coffee, tea, blueberries, and sunflower seeds | It shows hypoglycemic and hypolipidemic effects, Antibacterial, Antioxidant, Antiinflammatory, Improves insulin function, and shows antidiabetic properties. | α-amylase | 1410±400μg/mL | [(Aleixandre et al., 2022)](https://www.zotero.org/google-docs/?7oCn6u) [(Yan et al., 2020)](https://www.zotero.org/google-docs/?97HClp) |
|  | Tannic acid | Fruits like *Punica grantum* , *Diospyros kaki* , *Vitis vinifera* | Grapes, green tea, persimmons | Anti-inflammatory, neuroprotective, antitumor, cardioprotective, and anti-  pathogenic effects | α-amylase | 4.87μg/mL | [(Jing et al., 2022)](https://www.zotero.org/google-docs/?8iVGsx) [(Lou et al., 2018)](https://www.zotero.org/google-docs/?pU3CFN) |
|  | Oleanolic acid | Olive oil ( *Olea europea*), Rose apple, Garlic | Olive oil, Rose apple, Garlic, *Phytolacca americana, Olea europea, Phoradendron juniperinum, Hyptis capitata, Ocimum tenuiflorum* | Antioxidant, anti inflammatory, protection against chemically induced liver toxicity, Stimulate glucose metabolism | glycogen phosphorylase | 5.4 μm  2.466 μg/mL | [(Pollier & Goossens, 2012)](https://www.zotero.org/google-docs/?46wJla) |
|  | Silibinin | Milk thistle (*Silybum marianum),* etc | Milk thistle (*Silybum marianum)* | enhances glycogen synthesis, expression of glucagon-like peptide -1 receptor in the duodenum, reduces gluconeogenesis, attenuates blood glucose, inhibition of α- glucosidase | α- glucosidase | 16.0 µM  7.719 μg/mL | [(F. Xu et al., 2018)](https://www.zotero.org/google-docs/?vjxRSD) [(*Design, Synthesis, and Biological Activity of Conformationally Restricted Analogues of Silibinin \| ACS Omega*, n.d.)](https://www.zotero.org/google-docs/?rlZy9p) |


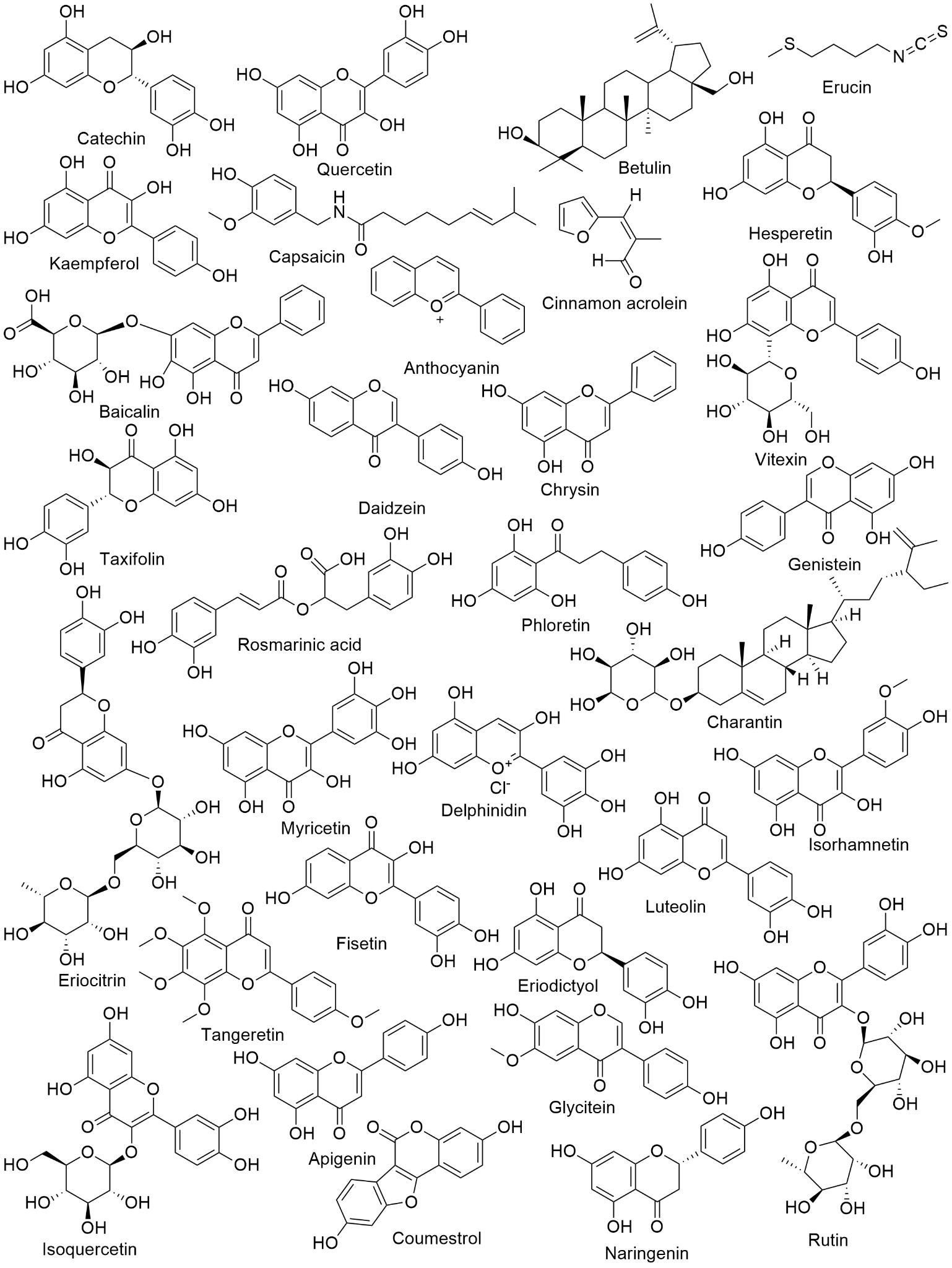


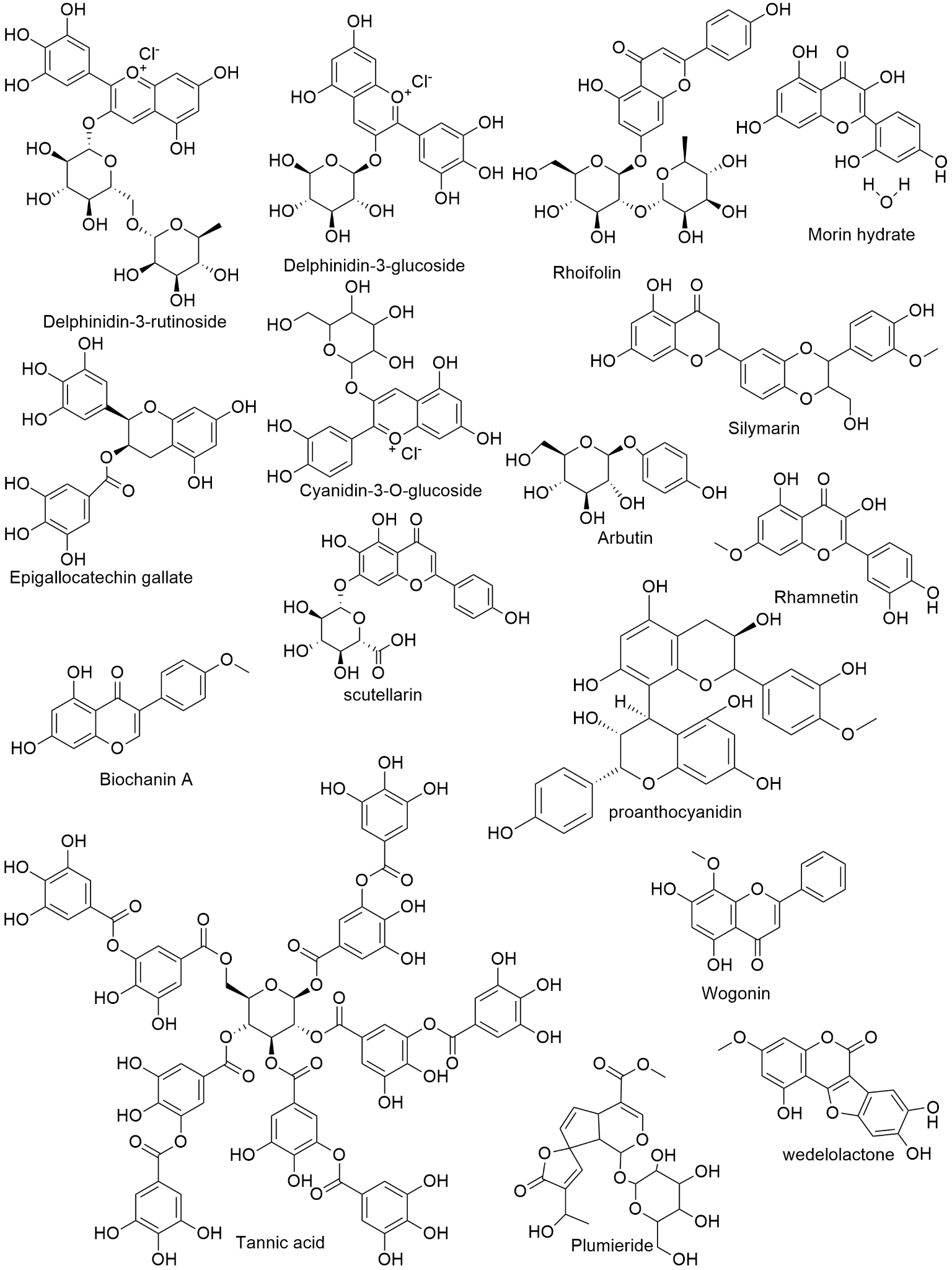


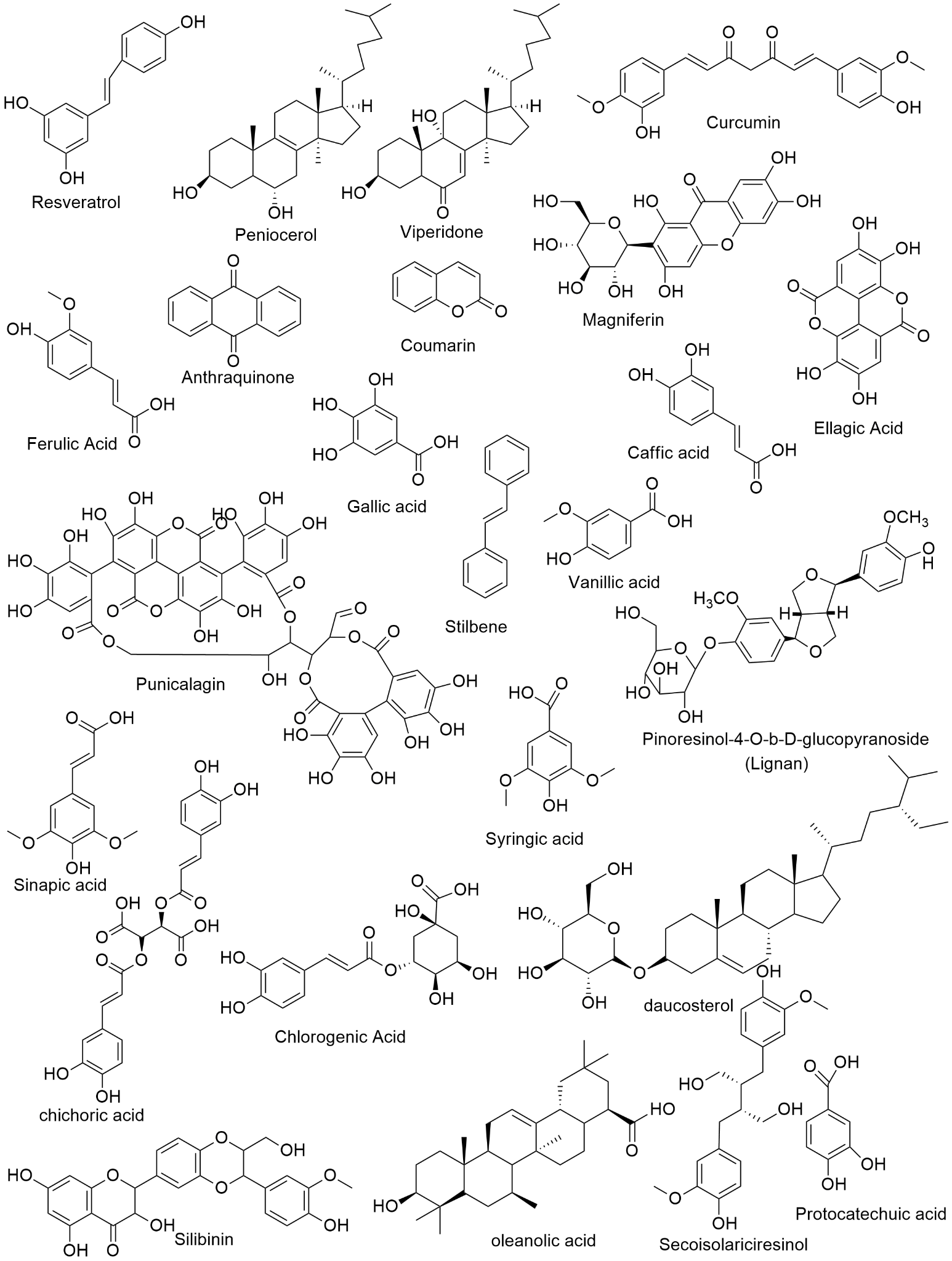

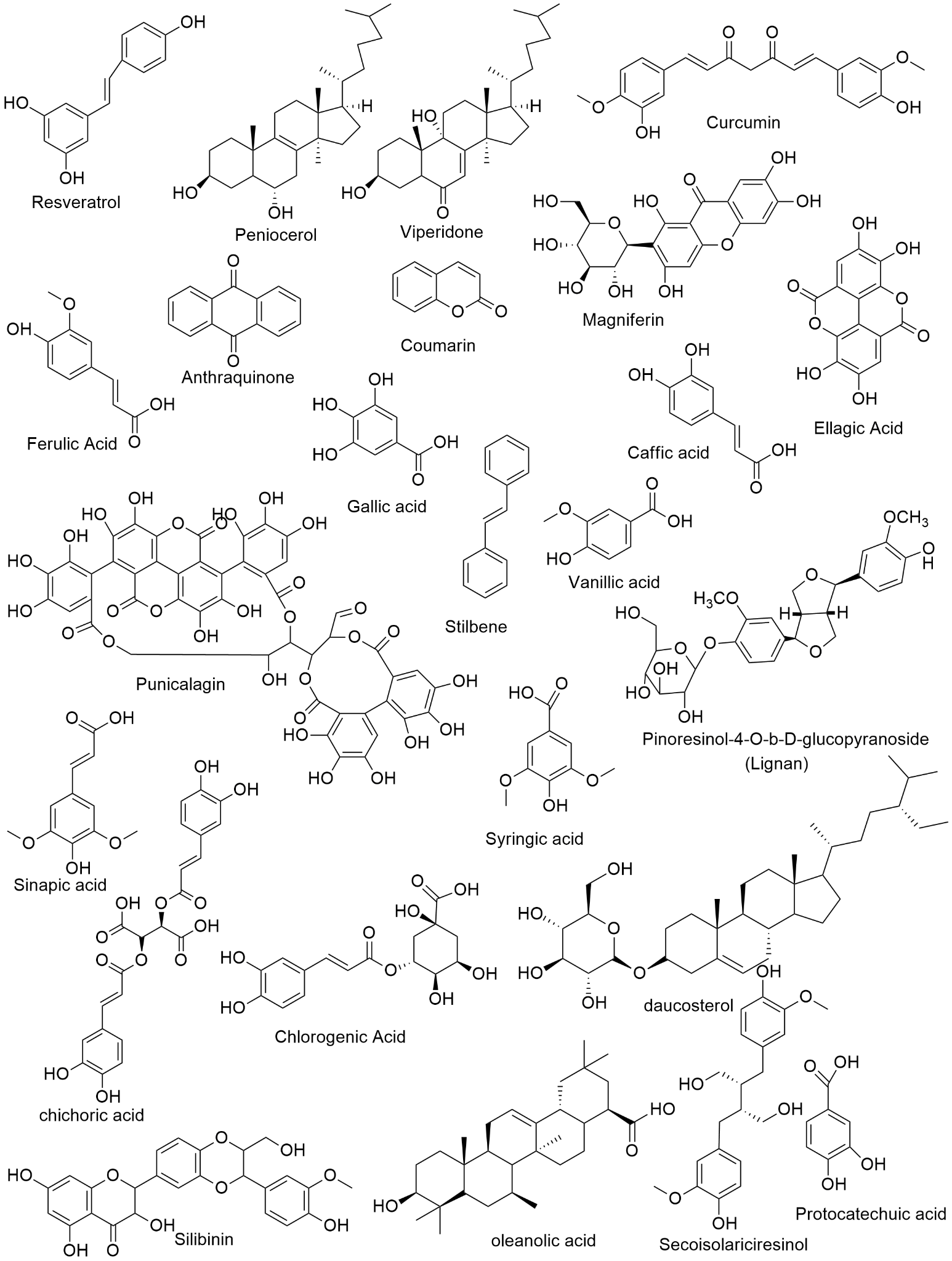


References

[*A Review on Protocatechuic Acid and Its Pharmacological Potential*. (n.d.). Retrieved April 14, 2023, from https://www.hindawi.com/journals/isrn/2014/952943/](https://www.zotero.org/google-docs/?OndkLv)

[Ahmad, N., Hasan, N., Ahmad, Z., Zishan, M., & Zohrameena, S. (2016). MOMORDICA CHARANTIA: FOR TRADITIONAL USES AND PHARMACOLOGICAL ACTIONS. *Journal of Drug Delivery and Therapeutics*, *6*(2), 40–44. https://doi.org/10.22270/jddt.v6i2.1202](https://www.zotero.org/google-docs/?OndkLv)

[Alam, S., Dhar, A., Hasan, M., Richi, F. T., Emon, N. U., Aziz, M. A., Mamun, A. A., Chowdhury, M. N. R., Hossain, M. J., Kim, J. K., Kim, B., Hasib, M. S., Zihad, S. M. N. K., Haque, M. R., Mohamed, I. N., & Rashid, M. A. (2022). Antidiabetic Potential of Commonly Available Fruit Plants in Bangladesh: Updates on Prospective Phytochemicals and Their Reported MoAs. *Molecules*, *27*(24), Article 24. https://doi.org/10.3390/molecules27248709](https://www.zotero.org/google-docs/?OndkLv)

[Alam, S., Sarker, Md. M. R., Sultana, T. N., Chowdhury, Md. N. R., Rashid, M. A., Chaity, N. I., Zhao, C., Xiao, J., Hafez, E. E., Khan, S. A., & Mohamed, I. N. (2022). Antidiabetic Phytochemicals From Medicinal Plants: Prospective Candidates for New Drug Discovery and Development. *Frontiers in Endocrinology*, *13*. https://www.frontiersin.org/articles/10.3389/fendo.2022.800714](https://www.zotero.org/google-docs/?OndkLv)

[Alegbe, E. O., Teralı, K., Olofinsan, K. A., Surgun, S., Ogbaga, C. C., & Ajiboye, T. O. (2019). Antidiabetic activity-guided isolation of gallic and protocatechuic acids from Hibiscus sabdariffa calyxes. *Journal of Food Biochemistry*, *43*(7), e12927. https://doi.org/10.1111/jfbc.12927](https://www.zotero.org/google-docs/?OndkLv)

[Aleixandre, A., Gil, J. V., Sineiro, J., & Rosell, C. M. (2022). Understanding phenolic acids inhibition of α-amylase and α-glucosidase and influence of reaction conditions. *Food Chemistry*, *372*, 131231. https://doi.org/10.1016/j.foodchem.2021.131231](https://www.zotero.org/google-docs/?OndkLv)

[Alshehri, M. M., Sharifi-Rad, J., Herrera-Bravo, J., Jara, E. L., Salazar, L. A., Kregiel, D., Uprety, Y., Akram, M., Iqbal, M., Martorell, M., Torrens-Mas, M., Pons, D. G., Daştan, S. D., Cruz-Martins, N., Ozdemir, F. A., Kumar, M., & Cho, W. C. (2021). Therapeutic Potential of Isoflavones with an Emphasis on Daidzein. *Oxidative Medicine and Cellular Longevity*, *2021*, 1–15. https://doi.org/10.1155/2021/6331630](https://www.zotero.org/google-docs/?OndkLv)

[Amor, A. J., Gómez-Guerrero, C., Ortega, E., Sala-Vila, A., & Lázaro, I. (2020). Ellagic Acid as a Tool to Limit the Diabetes Burden: Updated Evidence. *Antioxidants*, *9*(12), Article 12. https://doi.org/10.3390/antiox9121226](https://www.zotero.org/google-docs/?OndkLv)

[*Antioxidant, Antidiabetic, and Antihypertension Inhibitory Potentials of Phenolic Rich Medicinal Plants*. (n.d.). Retrieved March 5, 2023, from https://www.hindawi.com/journals/jchem/2022/9046780/](https://www.zotero.org/google-docs/?OndkLv)

[Arai, Y., Watanabe, S., Kimira, M., Shimoi, K., Mochizuki, R., & Kinae, N. (2000). Dietary Intakes of Flavonols, Flavones and Isoflavones by Japanese Women and the Inverse Correlation between Quercetin Intake and Plasma LDL Cholesterol Concentration. *The Journal of Nutrition*, *130*(9), 2243–2250. https://doi.org/10.1093/jn/130.9.2243](https://www.zotero.org/google-docs/?OndkLv)

[Aryaeian, N., Khorshidi Sedehi, S., & Arablou, T. (2017). Polyphenols and their effects on diabetes management: A review. *Medical Journal of the Islamic Republic of Iran*, *31*(1), 886–892. https://doi.org/10.14196/mjiri.31.134](https://www.zotero.org/google-docs/?OndkLv)

[Banihani, S., Swedan, S., & Alguraan, Z. (2013). Pomegranate and type 2 diabetes. *Nutrition Research*, *33*(5), 341–348. https://doi.org/10.1016/j.nutres.2013.03.003](https://www.zotero.org/google-docs/?OndkLv)

[Bhathena, S. J., & Velasquez, M. T. (2002). Beneficial role of dietary phytoestrogens in obesity and diabetes,. *The American Journal of Clinical Nutrition*, *76*(6), 1191–1201. https://doi.org/10.1093/ajcn/76.6.1191](https://www.zotero.org/google-docs/?OndkLv)

[Boeing, T., de Souza, P., Bonomini, T. J., Mariano, L. N. B., Somensi, L. B., Lucinda, R. M., Malheiros, A., da Silva, L. M., & Andrade, S. F. de. (2018). Antioxidant and anti-inflammatory effect of plumieride in dextran sulfate sodium-induced colitis in mice. *Biomedicine & Pharmacotherapy*, *99*, 697–703. https://doi.org/10.1016/j.biopha.2018.01.142](https://www.zotero.org/google-docs/?OndkLv)

[Cai, Y., Wu, L., Lin, X., Hu, X., & Wang, L. (2020). Phenolic profiles and screening of potential α-glucosidase inhibitors from Polygonum aviculare L. leaves using ultra-filtration combined with HPLC-ESI-qTOF-MS/MS and molecular docking analysis. *Industrial Crops and Products*, *154*, 112673. https://doi.org/10.1016/j.indcrop.2020.112673](https://www.zotero.org/google-docs/?OndkLv)

[Cásedas, G., Les, F., González-Burgos, E., Gómez-Serranillos, M. P., Smith, C., & López, V. (2019). Cyanidin-3-O-glucoside inhibits different enzymes involved in central nervous system pathologies and type-2 diabetes. *South African Journal of Botany*, *120*, 241–246. https://doi.org/10.1016/j.sajb.2018.07.001](https://www.zotero.org/google-docs/?OndkLv)

[*Chemistry & Biology Interface | January-February 2012, Volume 2, No.1*. (n.d.). Retrieved April 14, 2023, from https://cbijournal.com/january-february-2012.php](https://www.zotero.org/google-docs/?OndkLv)

[Cheong, S. H., Furuhashi, K., Ito, K., Nagaoka, M., Yonezawa, T., Miura, Y., & Yagasaki, K. (2014). Daidzein promotes glucose uptake through glucose transporter 4 translocation to plasma membrane in L6 myocytes and improves glucose homeostasis in Type 2 diabetic model mice. *The Journal of Nutritional Biochemistry*, *25*(2), 136–143. https://doi.org/10.1016/j.jnutbio.2013.09.012](https://www.zotero.org/google-docs/?OndkLv)

[*Chicoric acid, a new compound able to enhance insulin release and glucose uptake—ScienceDirect*. (n.d.). Retrieved April 14, 2023, from https://www.sciencedirect.com/science/article/abs/pii/S0006291X08018639](https://www.zotero.org/google-docs/?OndkLv)

[Choi, M. S., Jung, U. J., Yeo, J., Kim, M. J., & Lee, M. K. (2008). Genistein and daidzein prevent diabetes onset by elevating insulin level and altering hepatic gluconeogenic and lipogenic enzyme activities in non-obese diabetic (NOD) mice. *Diabetes/Metabolism Research and Reviews*, *24*(1), 74–81. https://doi.org/10.1002/dmrr.780](https://www.zotero.org/google-docs/?OndkLv)

[Choo, S.-J., Ryoo, I.-J., Kim, Y.-H., Xu, G.-H., Kim, W.-G., Kim, K.-H., Moon, S.-J., Son, E.-D., Bae, K., & Yoo, I.-D. (2010). Silymarin inhibits melanin synthesis in melanocyte cells. *Journal of Pharmacy and Pharmacology*, *61*(5), 663–667. https://doi.org/10.1211/jpp.61.05.0016](https://www.zotero.org/google-docs/?OndkLv)

[Chu, C., Deng, J., Man, Y., & Qu, Y. (2017). Green Tea Extracts Epigallocatechin-3-gallate for Different Treatments. *BioMed Research International*, *2017*, 1–9. https://doi.org/10.1155/2017/5615647](https://www.zotero.org/google-docs/?OndkLv)

[Ciddi, V., & Dodda, D. (2014). Therapeutic potential of resveratrol in diabetic complications: In vitro and in vivo studies. *Pharmacological Reports*, *66*(5), 799–803. https://doi.org/10.1016/j.pharep.2014.04.006](https://www.zotero.org/google-docs/?OndkLv)

[Cirmi, S., Ferlazzo, N., Lombardo, G., Maugeri, A., Calapai, G., Gangemi, S., & Navarra, M. (2016). Chemopreventive Agents and Inhibitors of Cancer Hallmarks: May Citrus Offer New Perspectives? *Nutrients*, *8*(11), 698. https://doi.org/10.3390/nu8110698](https://www.zotero.org/google-docs/?OndkLv)

[*Cyanidin-3-glucoside from Black Rice Ameliorates Diabetic Nephropathy via Reducing Blood Glucose, Suppressing Oxidative Stress and Inflammation, and Regulating Transforming Growth Factor β1/Smad Expression | Journal of Agricultural and Food Chemistry*. (n.d.). Retrieved April 12, 2023, from https://pubs.acs.org/doi/10.1021/acs.jafc.0c00680](https://www.zotero.org/google-docs/?OndkLv)

[Da Pozzo, E., Costa, B., Cavallini, C., Testai, L., Martelli, A., Calderone, V., & Martini, C. (2017). The Citrus Flavanone Naringenin Protects Myocardial Cells against Age-Associated Damage. *Oxidative Medicine and Cellular Longevity*, *2017*, 1–12. https://doi.org/10.1155/2017/9536148](https://www.zotero.org/google-docs/?OndkLv)

[Damián-Medina, K., Salinas-Moreno, Y., Milenkovic, D., Figueroa-Yáñez, L., Marino-Marmolejo, E., Higuera-Ciapara, I., Vallejo-Cardona, A., & Lugo-Cervantes, E. (2020). In silico analysis of antidiabetic potential of phenolic compounds from blue corn (Zea mays L.) and black bean (Phaseolus vulgaris L.). *Heliyon*, *6*(3), e03632. https://doi.org/10.1016/j.heliyon.2020.e03632](https://www.zotero.org/google-docs/?OndkLv)

[Demir, Y., Durmaz, L., Taslimi, P., & Gulçin, İ. (2019). Antidiabetic properties of dietary phenolic compounds: Inhibition effects on α‐amylase, aldose reductase, and α‐glycosidase. *Biotechnology and Applied Biochemistry*, *66*(5), 781–786. https://doi.org/10.1002/bab.1781](https://www.zotero.org/google-docs/?OndkLv)

[Den Hartogh, D. J., Gabriel, A., & Tsiani, E. (2020). Antidiabetic Properties of Curcumin II: Evidence from In Vivo Studies. *Nutrients*, *12*(1), Article 1. https://doi.org/10.3390/nu12010058](https://www.zotero.org/google-docs/?OndkLv)

[*Design, Synthesis, and Biological Activity of Conformationally Restricted Analogues of Silibinin | ACS Omega*. (n.d.). Retrieved April 14, 2023, from https://pubs.acs.org/doi/10.1021/acsomega.0c02936](https://www.zotero.org/google-docs/?OndkLv)

[Dewanjee, S., Chakraborty, P., Mukherjee, B., & De Feo, V. (2020). Plant-Based Antidiabetic Nanoformulations: The Emerging Paradigm for Effective Therapy. *International Journal of Molecular Sciences*, *21*(6), 2217. https://doi.org/10.3390/ijms21062217](https://www.zotero.org/google-docs/?OndkLv)

[Dixon, M., Woodrick, J., Gupta, S., Karmahapatra, S. K., Devito, S., Vasudevan, S., Dakshanamurthy, S., Adhikari, S., Yenugonda, V. M., & Roy, R. (2015). Naturally occurring polyphenol, morin hydrate, inhibits enzymatic activity of N-methylpurine DNA glycosylase, a DNA repair enzyme with various roles in human disease. *Bioorganic & Medicinal Chemistry*, *23*(5), 1102–1111. https://doi.org/10.1016/j.bmc.2014.12.067](https://www.zotero.org/google-docs/?OndkLv)

[El Omari, N., Jaouadi, I., Lahyaoui, M., Benali, T., Taha, D., Bakrim, S., El Menyiy, N., El Kamari, F., Zengin, G., Bangar, S. P., Lorenzo, J. M., Gallo, M., Montesano, D., & Bouyahya, A. (2022). Natural Sources, Pharmacological Properties, and Health Benefits of Daucosterol: Versatility of Actions. *Applied Sciences*, *12*(12), Article 12. https://doi.org/10.3390/app12125779](https://www.zotero.org/google-docs/?OndkLv)

[Eldahshan, O. (2013). Rhoifolin; A Potent Antiproliferative Effect on Cancer Cell Lines. *British Journal of Pharmaceutical Research*, *3*(1), 46–53. https://doi.org/10.9734/BJPR/2013/1864](https://www.zotero.org/google-docs/?OndkLv)

[Espíndola, K. M. M., Ferreira, R. G., Narvaez, L. E. M., Silva Rosario, A. C. R., da Silva, A. H. M., Silva, A. G. B., Vieira, A. P. O., & Monteiro, M. C. (2019). Chemical and Pharmacological Aspects of Caffeic Acid and Its Activity in Hepatocarcinoma. *Frontiers in Oncology*, *9*. https://www.frontiersin.org/articles/10.3389/fonc.2019.00541](https://www.zotero.org/google-docs/?OndkLv)

[Fan, J., Johnson, M. H., Lila, M. A., Yousef, G., & de Mejia, E. G. (2013). Berry and Citrus Phenolic Compounds Inhibit Dipeptidyl Peptidase IV: Implications in Diabetes Management. *Evidence-Based Complementary and Alternative Medicine*, *2013*, 1–13. https://doi.org/10.1155/2013/479505](https://www.zotero.org/google-docs/?OndkLv)

[Ferreira, P. S., Manthey, J. A., Nery, M. S., & Cesar, T. B. (2021). Pharmacokinetics and Biodistribution of Eriocitrin in Rats. *Journal of Agricultural and Food Chemistry*, *69*(6), 1796–1805. https://doi.org/10.1021/acs.jafc.0c04553](https://www.zotero.org/google-docs/?OndkLv)

[Fu, Y., Luo, J., Jia, Z., Zhen, W., Zhou, K., Gilbert, E., & Liu, D. (2014). Baicalein Protects against Type 2 Diabetes via Promoting Islet *β*-Cell Function in Obese Diabetic Mice. *International Journal of Endocrinology*, *2014*, e846742. https://doi.org/10.1155/2014/846742](https://www.zotero.org/google-docs/?OndkLv)

[*Glucose Uptake and α-Glycosidase Inhibition Activities of Secoisolariciresinol Diglucoside Isolated from Linum usitatissimum: An In vitro Study | Bioscience Biotechnology Research Communications*. (n.d.). Retrieved April 14, 2023, from https://bbrc.in/glucose-uptake-and-%ce%b1-glycosidase-inhibition-activities-of-secoisolariciresinol-diglucoside-isolated-from-linum-usitatissimum-an-in-vitro-study/](https://www.zotero.org/google-docs/?OndkLv)

[Habtemariam, S. (2012). The anti-obesity potential of sigmoidin A. *Pharmaceutical Biology*, *50*(12), 1519–1522. https://doi.org/10.3109/13880209.2012.688838](https://www.zotero.org/google-docs/?OndkLv)

[Habtemariam, S., & Lentini, G. (2015). The Therapeutic Potential of Rutin for Diabetes: An Update. *Mini-Reviews in Medicinal Chemistry*, *15*(7), 524–528. https://doi.org/10.2174/138955751507150424103721](https://www.zotero.org/google-docs/?OndkLv)

[Han, L., Fang, C., Zhu, R., Peng, Q., Li, D., & Wang, M. (2017). Inhibitory effect of phloretin on α-glucosidase: Kinetics, interaction mechanism and molecular docking. *International Journal of Biological Macromolecules*, *95*, 520–527. https://doi.org/10.1016/j.ijbiomac.2016.11.089](https://www.zotero.org/google-docs/?OndkLv)

[Han, L., Zhang, L., Ma, W., Li, D., Shi, R., & Wang, M. (2018). Proanthocyanidin B _2_ attenuates postprandial blood glucose and its inhibitory effect on alpha-glucosidase: Analysis by kinetics, fluorescence spectroscopy, atomic force microscopy and molecular docking. *Food & Function*, *9*(9), 4673–4682. https://doi.org/10.1039/C8FO00993G](https://www.zotero.org/google-docs/?OndkLv)

[Harnly, J. M., Doherty, R. F., Beecher, G. R., Holden, J. M., Haytowitz, D. B., Bhagwat, S., & Gebhardt, S. (2006). Flavonoid Content of U.S. Fruits, Vegetables, and Nuts. *Journal of Agricultural and Food Chemistry*, *54*(26), 9966–9977. https://doi.org/10.1021/jf061478a](https://www.zotero.org/google-docs/?OndkLv)

[He, M., Min, J.-W., Kong, W.-L., He, X.-H., Li, J.-X., & Peng, B.-W. (2016). A review on the pharmacological effects of vitexin and isovitexin. *Fitoterapia*, *115*, 74–85. https://doi.org/10.1016/j.fitote.2016.09.011](https://www.zotero.org/google-docs/?OndkLv)

[Ibitoye, O. B., Uwazie, J. N., & Ajiboye, T. O. (2018). Bioactivity-guided isolation of kaempferol as the antidiabetic principle from *Cucumis sativus* L. fruits. *Journal of Food Biochemistry*, *42*(4), e12479. https://doi.org/10.1111/jfbc.12479](https://www.zotero.org/google-docs/?OndkLv)

[Inui, A., Cheng, K.-C., Asakawa, A., Amitani, H., Amitani, M., Morinaga, A., Takimoto, Y., Kairupan, B. H. R., & Runtuwene, J. (2016). Rosmarinic acid ameliorates hyperglycemia and insulin sensitivity in diabetic rats, potentially by modulating the expression of PEPCK and GLUT4. *Drug Design, Development and Therapy*, *Volume 10*, 2193–2202. https://doi.org/10.2147/DDDT.S108539](https://www.zotero.org/google-docs/?OndkLv)

[Islam, A., Islam, M. S., Rahman, M. K., Uddin, M. N., & Akanda, M. R. (2020). The pharmacological and biological roles of eriodictyol. *Archives of Pharmacal Research*, *43*(6), 582–592. https://doi.org/10.1007/s12272-020-01243-0](https://www.zotero.org/google-docs/?OndkLv)

[Islam, F., Khadija, J. F., Islam, Md. R., Shohag, S., Mitra, S., Alghamdi, S., Babalghith, A. O., Theyab, A., Rahman, M. T., Akter, A., Al Mamun, A., Alhumaydhi, F. A., & Emran, T. B. (2022). Investigating Polyphenol Nanoformulations for Therapeutic Targets against Diabetes Mellitus. *Evidence-Based Complementary and Alternative Medicine*, *2022*, 1–16. https://doi.org/10.1155/2022/5649156](https://www.zotero.org/google-docs/?OndkLv)

[Jeon, Y.-H., & Choi, S.-W. (2019). Isolation, Identification, and Quantification of Tyrosinase and α-Glucosidase Inhibitors from UVC-Irradiated Mulberry (Morus alba L.) Leaves. *Preventive Nutrition and Food Science*, *24*(1), 84–94. https://doi.org/10.3746/pnf.2019.24.1.84](https://www.zotero.org/google-docs/?OndkLv)

[Jeong, E.-Y., Cho, K.-S., & Lee, H.-S. (2012). α-amylase and α-glucosidase inhibitors isolated from Triticum aestivum L. sprouts. *Journal of the Korean Society for Applied Biological Chemistry*, *55*(1), Article 1. https://doi.org/10.1007/s13765-012-0008-1](https://www.zotero.org/google-docs/?OndkLv)

[Jiang, W., Kan, H., Li, P., Liu, S., & Liu, Z. (2015). Screening and structural characterization of potential α-glucosidase inhibitors from Radix Astragali flavonoids extract by ultrafiltration LC-DAD-ESI-MS ^n^. *Analytical Methods*, *7*(1), 123–128. https://doi.org/10.1039/C4AY02081B](https://www.zotero.org/google-docs/?OndkLv)

[Jing, W., Xiaolan, C., Yu, C., Feng, Q., & Haifeng, Y. (2022). Pharmacological effects and mechanisms of tannic acid. *Biomedicine & Pharmacotherapy*, *154*, 113561. https://doi.org/10.1016/j.biopha.2022.113561](https://www.zotero.org/google-docs/?OndkLv)

[Kalai, F. Z., Boulaaba, M., Ferdousi, F., & Isoda, H. (2022). Effects of Isorhamnetin on Diabetes and Its Associated Complications: A Review of In Vitro and In Vivo Studies and a Post Hoc Transcriptome Analysis of Involved Molecular Pathways. *International Journal of Molecular Sciences*, *23*(2), 704. https://doi.org/10.3390/ijms23020704](https://www.zotero.org/google-docs/?OndkLv)

[Kato, M., Tani, T., Terahara, N., & Tsuda, T. (2015). The Anthocyanin Delphinidin 3-Rutinoside Stimulates Glucagon-Like Peptide-1 Secretion in Murine GLUTag Cell Line via the Ca2+/Calmodulin-Dependent Kinase II Pathway. *PLOS ONE*, *10*(5), e0126157. https://doi.org/10.1371/journal.pone.0126157](https://www.zotero.org/google-docs/?OndkLv)

[Kim, M. S., Hur, H. J., Kwon, D. Y., & Hwang, J.-T. (2012). Tangeretin stimulates glucose uptake via regulation of AMPK signaling pathways in C2C12 myotubes and improves glucose tolerance in high-fat diet-induced obese mice. *Molecular and Cellular Endocrinology*, *358*(1), 127–134. https://doi.org/10.1016/j.mce.2012.03.013](https://www.zotero.org/google-docs/?OndkLv)

[Kiokias, S., Proestos, C., & Oreopoulou, V. (2020). Phenolic Acids of Plant Origin—A Review on Their Antioxidant Activity In Vitro (O/W Emulsion Systems) Along with Their in Vivo Health Biochemical Properties. *Foods*, *9*(4), Article 4. https://doi.org/10.3390/foods9040534](https://www.zotero.org/google-docs/?OndkLv)

[Kulkarni, P., Lohidasan, S., & Mahadik, K. (2021). Isolation, characterisation and investigation of *in vitro* antidiabetic and antioxidant activity of phytoconstituents from fruit of *Momordica charantia* Linn. *Natural Product Research*, *35*(6), 1035–1037. https://doi.org/10.1080/14786419.2019.1613400](https://www.zotero.org/google-docs/?OndkLv)

[Kumar, N., & Pruthi, V. (2014). Potential applications of ferulic acid from natural sources. *Biotechnology Reports*, *4*, 86–93. https://doi.org/10.1016/j.btre.2014.09.002](https://www.zotero.org/google-docs/?OndkLv)

[Kumar, V., Sharma, K., Ahmed, B., Al-Abbasi, F. A., Anwar, F., & Verma, A. (2018). Deconvoluting the dual hypoglycemic effect of wedelolactone isolated from *Wedelia calendulacea*: Investigation *via* experimental validation and molecular docking. *RSC Advances*, *8*(32), 18180–18196. https://doi.org/10.1039/C7RA12568B](https://www.zotero.org/google-docs/?OndkLv)

[Lai, D., Huang, M., Zhao, L., Tian, Y., Li, Y., Liu, D., Wu, Y., & Deng, F. (2019). Delphinidin-induced autophagy protects pancreatic &lt;bold&gt;&beta;&lt;/bold&gt; cells against apoptosis resulting from high-glucose stress via AMPK signaling pathway. *Acta Biochimica et Biophysica Sinica*, *51*(12), 1242–1249. https://doi.org/10.1093/abbs/gmz126](https://www.zotero.org/google-docs/?OndkLv)

[Lee, Y.-J., Choi, H.-S., Seo, M.-J., Jeon, H.-J., Kim, K.-J., & Lee, B.-Y. (2015). Kaempferol suppresses lipid accumulation by inhibiting early adipogenesis in 3T3-L1 cells and zebrafish. *Food & Function*, *6*(8), 2824–2833. https://doi.org/10.1039/C5FO00481K](https://www.zotero.org/google-docs/?OndkLv)

[Leporini, M., Loizzo, M. R., Tundis, R., La Torre, C., Fazio, A., & Plastina, P. (2019). Non-Pungent n-3 Polyunsaturated Fatty Acid (PUFA)-Derived Capsaicin Analogues as Potential Functional Ingredients with Antioxidant and Carbohydrate-Hydrolysing Enzyme Inhibitory Activities. *Antioxidants*, *8*(6), 162. https://doi.org/10.3390/antiox8060162](https://www.zotero.org/google-docs/?OndkLv)

[Li, H., Cao, W., Wei, L.-F., Xia, J.-Q., Gu, Y., Gu, L.-M., Pan, C.-Y., Liu, Y.-Q., Tian, Y.-Z., & Lu, M. (2021). Arbutin alleviates diabetic symptoms by attenuating oxidative stress in a mouse model of type 1 diabetes. *International Journal of Diabetes in Developing Countries*, *41*(4), 586–592. https://doi.org/10.1007/s13410-021-00920-0](https://www.zotero.org/google-docs/?OndkLv)

[Li, K., Yao, F., Xue, Q., Fan, H., Yang, L., Li, X., Sun, L., & Liu, Y. (2018). Inhibitory effects against α-glucosidase and α-amylase of the flavonoids-rich extract from Scutellaria baicalensis shoots and interpretation of structure–activity relationship of its eight flavonoids by a refined assign-score method. *Chemistry Central Journal*, *12*(1), 82. https://doi.org/10.1186/s13065-018-0445-y](https://www.zotero.org/google-docs/?OndkLv)

[Lin, D., Du, Q., Wang, H., Gao, G., Zhou, J., Ke, L., Chen, T., Shaw, C., & Rao, P. (2017). Antidiabetic Micro-/Nanoaggregates from Ge-Gen-Qin-Lian-Tang Decoction Increase Absorption of Baicalin and Cellular Antioxidant Activity In Vitro. *BioMed Research International*, *2017*, 1–8. https://doi.org/10.1155/2017/9217912](https://www.zotero.org/google-docs/?OndkLv)

[Lou, W., Chen, Y., Ma, H., Liang, G., & Liu, B. (2018). Antioxidant and α-amylase inhibitory activities of tannic acid. *Journal of Food Science and Technology*, *55*(9), 3640–3646. https://doi.org/10.1007/s13197-018-3292-x](https://www.zotero.org/google-docs/?OndkLv)

[Lucarini, Pagnotta, Micheli, Parisio, Testai, Martelli, Calderone, Matteo, Lazzeri, Mannelli, & Ghelardini. (2019). Eruca sativa Meal against Diabetic Neuropathic Pain: An H2S-Mediated Effect of Glucoerucin. *Molecules*, *24*(16), 3006. https://doi.org/10.3390/molecules24163006](https://www.zotero.org/google-docs/?OndkLv)

[Lv, L., Zhang, J., Tian, F., Li, X., Li, D., & Yu, X. (2019). Arbutin protects HK-2 cells against high glucose-induced apoptosis and autophagy by up-regulating microRNA-27a. *Artificial Cells, Nanomedicine, and Biotechnology*, *47*(1), 2940–2947. https://doi.org/10.1080/21691401.2019.1640231](https://www.zotero.org/google-docs/?OndkLv)

[Magaña-Barajas, E., Buitimea-Cantúa, G. V., Hernández-Morales, A., Torres-Pelayo, V. del R., Vázquez-Martínez, J., & Buitimea-Cantúa, N. E. (2021). In vitro α-amylase and α-glucosidase enzyme inhibition and antioxidant activity by capsaicin and piperine from Capsicum chinense and Piper nigrum fruits. *Journal of Environmental Science and Health, Part B*, *56*(3), 282–291. https://doi.org/10.1080/03601234.2020.1869477](https://www.zotero.org/google-docs/?OndkLv)

[Maradesha, T., Patil, S. M., Al-Mutairi, K. A., Ramu, R., Madhunapantula, S. V., & Alqadi, T. (2022). Inhibitory Effect of Polyphenols from the Whole Green Jackfruit Flour against α-Glucosidase, α-Amylase, Aldose Reductase and Glycation at Multiple Stages and Their Interaction: Inhibition Kinetics and Molecular Simulations. *Molecules*, *27*(6), Article 6. https://doi.org/10.3390/molecules27061888](https://www.zotero.org/google-docs/?OndkLv)

[Maradesha, T., Patil, S. M., Phanindra, B., Achar, R. R., Silina, E., Stupin, V., & Ramu, R. (2022). Multiprotein Inhibitory Effect of Dietary Polyphenol Rutin from Whole Green Jackfruit Flour Targeting Different Stages of Diabetes Mellitus: Defining a Bio-Computational Stratagem. *Separations*, *9*(9), 262. https://doi.org/10.3390/separations9090262](https://www.zotero.org/google-docs/?OndkLv)

[Martorell, M., Castro, N., Victoriano, M., Capó, X., Tejada, S., Vitalini, S., Pezzani, R., & Sureda, A. (2021). An Update of Anthraquinone Derivatives Emodin, Diacerein, and Catenarin in Diabetes. *Evidence-Based Complementary and Alternative Medicine*, *2021*, e3313419. https://doi.org/10.1155/2021/3313419](https://www.zotero.org/google-docs/?OndkLv)

[Mehta, V., Verma, P., Sharma, N., Sharma, A., Thakur, A., & Malairaman, U. (2017). Quercetin, ascorbic acid, caffeine and ellagic acid are more efficient than rosiglitazone, metformin and glimepiride in interfering with pathways leading to the development of neurological complications associated with diabetes: A comparative in-vitro study. *Bulletin of Faculty of Pharmacy, Cairo University*, *55*(1), 115–121. https://doi.org/10.1016/j.bfopcu.2016.12.002](https://www.zotero.org/google-docs/?OndkLv)

[Meng, Y., Su, A., Yuan, S., Zhao, H., Tan, S., Hu, C., Deng, H., & Guo, Y. (2016). Evaluation of Total Flavonoids, Myricetin, and Quercetin from Hovenia dulcis Thunb. As Inhibitors of α-Amylase and α-Glucosidase. *Plant Foods for Human Nutrition*, *71*(4), 444–449. https://doi.org/10.1007/s11130-016-0581-2](https://www.zotero.org/google-docs/?OndkLv)

[Miladinovic, B., Faria, M. Â., Ribeiro, M., Sobral, M. M. C., & Ferreira, I. M. P. L. V. O. (2023). Delphinidin-3-rutinoside from Blackcurrant Berries (Ribes nigrum): In Vitro Antiproliferative Activity and Interactions with Other Phenolic Compounds. *Molecules*, *28*(3), 1286. https://doi.org/10.3390/molecules28031286](https://www.zotero.org/google-docs/?OndkLv)

[Milella, L., Milazzo, S., De Leo, M., Vera Saltos, M. B., Faraone, I., Tuccinardi, T., Lapillo, M., De Tommasi, N., & Braca, A. (2016). α-Glucosidase and α-Amylase Inhibitors from *Arcytophyllum thymifolium*. *Journal of Natural Products*, *79*(8), 2104–2112. https://doi.org/10.1021/acs.jnatprod.6b00484](https://www.zotero.org/google-docs/?OndkLv)

[Minocha, T., Birla, H., Obaid, A. A., Rai, V., Sushma, P., Shivamallu, C., Moustafa, M., Al-Shehri, M., Al-Emam, A., Tikhonova, M. A., Yadav, S. K., Poeggeler, B., Singh, D., & Singh, S. K. (2022). Flavonoids as Promising Neuroprotectants and Their Therapeutic Potential against Alzheimer’s Disease. *Oxidative Medicine and Cellular Longevity*, *2022*, 1–13. https://doi.org/10.1155/2022/6038996](https://www.zotero.org/google-docs/?OndkLv)

[Mirza, A. C., Panchal, S. S., Allam, A. A., Othman, S. I., Satia, M., & Mandhane, S. N. (2022). Syringic Acid Ameliorates Cardiac, Hepatic, Renal and Neuronal Damage Induced by Chronic Hyperglycaemia in Wistar Rats: A Behavioural, Biochemical and Histological Analysis. *Molecules*, *27*(19), Article 19. https://doi.org/10.3390/molecules27196722](https://www.zotero.org/google-docs/?OndkLv)

[Morais, F. S., Canuto, K. M., Ribeiro, P. R. V., Silva, A. B., Pessoa, O. D. L., Freitas, C. D. T., Alencar, N. M. N., Oliveira, A. C., & Ramos, M. V. (2020). Chemical profiling of secondary metabolites from Himatanthus drasticus (Mart.) Plumel latex with inhibitory action against the enzymes α-amylase and α-glucosidase: In vitro and in silico assays. *Journal of Ethnopharmacology*, *253*, 112644. https://doi.org/10.1016/j.jep.2020.112644](https://www.zotero.org/google-docs/?OndkLv)

[Mrabti, H., Jaradat, N., Fichtali, I., Ouedrhiri, W., Jodeh, S., Ayesh, S., Cherrah, Y., & Faouzi, M. (2018). Separation, Identification, and Antidiabetic Activity of Catechin Isolated from Arbutus unedo L. Plant Roots. *Plants*, *7*(2), 31. https://doi.org/10.3390/plants7020031](https://www.zotero.org/google-docs/?OndkLv)

[Muñoz-Gómez, R. J., Rivero-Cruz, I., Ovalle-Magallanes, B., Linares, E., Bye, R., Tovar, A. R., Noriega, L. G., Tovar-Palacio, C., & Mata, R. (2022). Antidiabetic Sterols from Peniocereus greggii Roots. *ACS Omega*, *7*(15), 13144–13154. https://doi.org/10.1021/acsomega.2c00595](https://www.zotero.org/google-docs/?OndkLv)

[Ni, M., Hu, X., Gong, D., & Zhang, G. (2020). Inhibitory mechanism of vitexin on α-glucosidase and its synergy with acarbose. *Food Hydrocolloids*, *105*, 105824. https://doi.org/10.1016/j.foodhyd.2020.105824](https://www.zotero.org/google-docs/?OndkLv)

[Nie, Y., & Stürzenbaum, S. R. (2019). Proanthocyanidins of Natural Origin: Molecular Mechanisms and Implications for Lipid Disorder and Aging-Associated Diseases. *Advances in Nutrition*, *10*(3), 464–478. https://doi.org/10.1093/advances/nmy118](https://www.zotero.org/google-docs/?OndkLv)

[[No title found]. (n.d.). *International Journal Of Pharmaceutical Sciences And Research*, *2*(3).](https://www.zotero.org/google-docs/?OndkLv)

[Noor, H., Cao, P., & Raleigh, D. P. (2012). Morin hydrate inhibits amyloid formation by islet amyloid polypeptide and disaggregates amyloid fibers. *Protein Science*, *21*(3), 373–382. https://doi.org/10.1002/pro.2023](https://www.zotero.org/google-docs/?OndkLv)

[Olennikov, D. N., & Chirikova, N. K. (2018). Rhamnetin Glycosides from the Genus Spiraea. *Chemistry of Natural Compounds*, *54*(1), 41–45. https://doi.org/10.1007/s10600-018-2255-9](https://www.zotero.org/google-docs/?OndkLv)

[Oliveira, H., Fernandes, A., F. Brás, N., Mateus, N., de Freitas, V., & Fernandes, I. (2020). Anthocyanins as Antidiabetic Agents—In Vitro and In Silico Approaches of Preventive and Therapeutic Effects. *Molecules*, *25*(17), 3813. https://doi.org/10.3390/molecules25173813](https://www.zotero.org/google-docs/?OndkLv)

[Oza, M. J., & Kulkarni, Y. A. (2018). Biochanin A improves insulin sensitivity and controls hyperglycemia in type 2 diabetes. *Biomedicine & Pharmacotherapy*, *107*, 1119–1127. https://doi.org/10.1016/j.biopha.2018.08.073](https://www.zotero.org/google-docs/?OndkLv)

[Pari, L., Rajarajeswari, N., Saravanan, S., & Rathinam, A. (2014). Antihyperlipidemic effect of coumarin in experimental type 2 diabetic rats. *Biomedicine & Preventive Nutrition*, *4*(2), 171–176. https://doi.org/10.1016/j.bionut.2014.02.003](https://www.zotero.org/google-docs/?OndkLv)

[Park, M.-H., Ju, J.-W., Park, M., & Han, J. (2013). Daidzein inhibits carbohydrate digestive enzymes in vitro and alleviates postprandial hyperglycemia in diabetic mice. *European Journal of Pharmacology*, *712*(1–3), 48–52. https://doi.org/10.1016/j.ejphar.2013.04.047](https://www.zotero.org/google-docs/?OndkLv)

[Paun, G., Neagu, E., Albu, C., Savin, S., & Radu, G. L. (2020). *In Vitro* Evaluation of Antidiabetic and Anti-Inflammatory Activities of Polyphenolic-Rich Extracts from *Anchusa officinalis* and *Melilotus officinalis*. *ACS Omega*, *5*(22), 13014–13022. https://doi.org/10.1021/acsomega.0c00929](https://www.zotero.org/google-docs/?OndkLv)

[Pleşca-Manea, L., Pârvu, A. E., Pârvu, M., Taaˇmaş, M., Buia, R., & Puia, M. (2002). Effects of *Melilotus officinalis* on acute inflammation: *MELILOTUS OFFICINALIS* ON INFLAMMATION. *Phytotherapy Research*, *16*(4), 316–319. https://doi.org/10.1002/ptr.875](https://www.zotero.org/google-docs/?OndkLv)

[Pollier, J., & Goossens, A. (2012). Oleanolic acid. *Phytochemistry*, *77*, 10–15. https://doi.org/10.1016/j.phytochem.2011.12.022](https://www.zotero.org/google-docs/?OndkLv)

[Prasad, K. (2000). Antioxidant activity of secoisolariciresinol diglucoside-derived metabolites, secoisolariciresinol, Enterodiol, and enterolactone. *International Journal of Angiology*, *9*(04), 220–225. https://doi.org/10.1007/BF01623898](https://www.zotero.org/google-docs/?OndkLv)

[Priscilla, D. H., Roy, D., Suresh, A., Kumar, V., & Thirumurugan, K. (2014). Naringenin inhibits α-glucosidase activity: A promising strategy for the regulation of postprandial hyperglycemia in high fat diet fed streptozotocin induced diabetic rats. *Chemico-Biological Interactions*, *210*, 77–85. https://doi.org/10.1016/j.cbi.2013.12.014](https://www.zotero.org/google-docs/?OndkLv)

[Promyos, N., Temviriyanukul, P., & Suttisansanee, U. (2020). Investigation of Anthocyanidins and Anthocyanins for Targeting α-Glucosidase in Diabetes Mellitus. *Preventive Nutrition and Food Science*, *25*(3), 263–271. https://doi.org/10.3746/pnf.2020.25.3.263](https://www.zotero.org/google-docs/?OndkLv)

[Rahman, H. (2014). Antioxidant, Cytotoxic and Hypolipidemic Activities of Plumeria alba L. and Plumeria rubra L. *American Journal of Life Sciences*, *2*(6), 11. https://doi.org/10.11648/j.ajls.s.2014020601.13](https://www.zotero.org/google-docs/?OndkLv)

[Ramírez-Espinosa, J., Saldaña-Ríos, J., García-Jiménez, S., Villalobos-Molina, R., Ávila-Villarreal, G., Rodríguez-Ocampo, A., Bernal-Fernández, G., & Estrada-Soto, S. (2017). Chrysin Induces Antidiabetic, Antidyslipidemic and Anti-Inflammatory Effects in Athymic Nude Diabetic Mice. *Molecules*, *23*(1), 67. https://doi.org/10.3390/molecules23010067](https://www.zotero.org/google-docs/?OndkLv)

[Revathy, J. (2017). The Role of Hesperetin in the Management of Diabetes Mellitus and Its Complications. *Journal of Cancer Treatment and Research*, *5*(1), 1. https://doi.org/10.11648/j.jctr.20170501.11](https://www.zotero.org/google-docs/?OndkLv)

[Rimoldi, G., Christoffel, J., Seidlova-Wuttke, D., Jarry, H., & Wuttke, W. (2007). Effects of Chronic Genistein Treatment in Mammary Gland, Uterus, and Vagina. *Environmental Health Perspectives*, *115*(Suppl 1), 62–68. https://doi.org/10.1289/ehp.9367](https://www.zotero.org/google-docs/?OndkLv)

[Różańska, D., & Regulska-Ilow, B. (2018). The significance of anthocyanins in the prevention and treatment of type 2 diabetes. *Advances in Clinical and Experimental Medicine*, *27*(1), 135–142. https://doi.org/10.17219/acem/64983](https://www.zotero.org/google-docs/?OndkLv)

[Sahib, A. (2016). Antidiabetic and Antioxidant Effect of Cinnamon in poorly Controlled Type-2 Diabetic Iraqi Patients: A Randomized, Placebo-Controlled Clinical Trial. *Journal of Intercultural Ethnopharmacology*, *5*(2), 108. https://doi.org/10.5455/jice.20160217044511](https://www.zotero.org/google-docs/?OndkLv)

[Salazar, J., Martinez-Vazquez, M., Cespedes-Acuña, C. L., Apan, M., Nieto, A., Rodríguez-Silverio, J., & Flores-Murrieta, F. (2011). Anti-Inflammatory and Cytotoxic Activities of Chichipegenin, Peniocerol, and Macdougallin Isolated from Myrtillocactus geometrizans (Mart. Ex Pfeiff.) Con. *Zeitschrift Für Naturforschung. C, Journal of Biosciences*, *66*, 24–30. https://doi.org/10.5560/ZNC.2011.66c0024](https://www.zotero.org/google-docs/?OndkLv)

[Sangeetha, R. (2019). Luteolin in the Management of Type 2 Diabetes Mellitus. *Current Research in Nutrition and Food Science Journal*, *7*(2), 393–398. https://doi.org/10.12944/CRNFSJ.7.2.09](https://www.zotero.org/google-docs/?OndkLv)

[Sekar, V., Chakraborty, S., Mani, S., Sali, V. K., & Vasanthi, H. R. (2019). Mangiferin from Mangifera indica fruits reduces post-prandial glucose level by inhibiting α-glucosidase and α-amylase activity. *South African Journal of Botany*, *120*, 129–134. https://doi.org/10.1016/j.sajb.2018.02.001](https://www.zotero.org/google-docs/?OndkLv)

[Semaming, Y., Pannengpetch, P., Chattipakorn, S. C., & Chattipakorn, N. (2015). Pharmacological Properties of Protocatechuic Acid and Its Potential Roles as Complementary Medicine. *Evidence-Based Complementary and Alternative Medicine*, *2015*, e593902. https://doi.org/10.1155/2015/593902](https://www.zotero.org/google-docs/?OndkLv)

[Sharifi-Rad, J., Quispe, C., Imran, M., Rauf, A., Nadeem, M., Gondal, T. A., Ahmad, B., Atif, M., Mubarak, M. S., Sytar, O., Zhilina, O. M., Garsiya, E. R., Smeriglio, A., Trombetta, D., Pons, D. G., Martorell, M., Cardoso, S. M., Razis, A. F. A., Sunusi, U., … Calina, D. (2021). Genistein: An Integrative Overview of Its Mode of Action, Pharmacological Properties, and Health Benefits. *Oxidative Medicine and Cellular Longevity*, *2021*, e3268136. https://doi.org/10.1155/2021/3268136](https://www.zotero.org/google-docs/?OndkLv)

[Shazmeen, Haq, I.-U., Rajoka, M. S. R., Asim Shabbir, M., Umair, M., Llah, I., Manzoor, M. F., Nemat, A., Abid, M., Khan, M. R., & Aadil, R. M. (2021). Role of stilbenes against insulin resistance: A review. *Food Science & Nutrition*, *9*(11), 6389–6405. https://doi.org/10.1002/fsn3.2553](https://www.zotero.org/google-docs/?OndkLv)

[Shen, Y., Xu, Z., & Sheng, Z. (2017). Ability of resveratrol to inhibit advanced glycation end product formation and carbohydrate-hydrolyzing enzyme activity, and to conjugate methylglyoxal. *Food Chemistry*, *216*, 153–160. https://doi.org/10.1016/j.foodchem.2016.08.034](https://www.zotero.org/google-docs/?OndkLv)

[Silveira, A. C., Dias, J. P., Santos, V. M., Oliveira, P. F., Alves, M. G., Rato, L., & Silva, B. M. (2019). The Action of Polyphenols in Diabetes Mellitus and Alzheimer’s Disease: A Common Agent for Overlapping Pathologies. *Current Neuropharmacology*, *17*(7), 590–613. https://doi.org/10.2174/1570159X16666180803162059](https://www.zotero.org/google-docs/?OndkLv)

[Singh, A.-K., Patel, P. K., Choudhary, K., Joshi, J., Yadav, D., & Jin, J.-O. (2020). Quercetin and Coumarin Inhibit Dipeptidyl Peptidase-IV and Exhibits Antioxidant Properties: In Silico, In Vitro, Ex Vivo. *Biomolecules*, *10*(2), 207. https://doi.org/10.3390/biom10020207](https://www.zotero.org/google-docs/?OndkLv)

[Song, X., Tan, L., Wang, M., Ren, C., Guo, C., Yang, B., Ren, Y., Cao, Z., Li, Y., & Pei, J. (2021). Myricetin: A review of the most recent research. *Biomedicine & Pharmacotherapy*, *134*, 111017. https://doi.org/10.1016/j.biopha.2020.111017](https://www.zotero.org/google-docs/?OndkLv)

[Soobrattee, M. A., Neergheen, V. S., Luximon-Ramma, A., Aruoma, O. I., & Bahorun, T. (2005). Phenolics as potential antioxidant therapeutic agents: Mechanism and actions. *Mutation Research/Fundamental and Molecular Mechanisms of Mutagenesis*, *579*(1), 200–213. https://doi.org/10.1016/j.mrfmmm.2005.03.023](https://www.zotero.org/google-docs/?OndkLv)

[Sri Balasubashini, M., Rukkumani, R., & Menon, V. P. (2003). Protective effects of ferulic acidon hyperlipidemic diabetic rats. *Acta Diabetologica*, *40*(3), 118–122. https://doi.org/10.1007/s00592-003-0099-6](https://www.zotero.org/google-docs/?OndkLv)

[Srinivasulu, C., Ramgopal, M., Ramanjaneyulu, G., Anuradha, C. M., & Suresh Kumar, C. (2018). Syringic acid (SA) ‒ A Review of Its Occurrence, Biosynthesis, Pharmacological and Industrial Importance. *Biomedicine & Pharmacotherapy*, *108*, 547–557. https://doi.org/10.1016/j.biopha.2018.09.069](https://www.zotero.org/google-docs/?OndkLv)

[Stolf, A. M., Campos Cardoso, C., Morais, H. de, Alves de Souza, C. E., Lomba, L. A., Brandt, A. P., Agnes, J. P., Collere, F. C., Galindo, C. M., Corso, C. R., Spercoski, K. M., Locatelli Dittrich, R., Zampronio, A. R., Cadena, S. M. S. C., & Acco, A. (2018). Effects of silymarin on angiogenesis and oxidative stress in streptozotocin-induced diabetes in mice. *Biomedicine & Pharmacotherapy*, *108*, 232–243. https://doi.org/10.1016/j.biopha.2018.09.042](https://www.zotero.org/google-docs/?OndkLv)

[Su, H., Ruan, Y.-T., Li, Y., Chen, J.-G., Yin, Z.-P., & Zhang, Q.-F. (2020). In vitro and in vivo inhibitory activity of taxifolin on three digestive enzymes. *International Journal of Biological Macromolecules*, *150*, 31–37. https://doi.org/10.1016/j.ijbiomac.2020.02.027](https://www.zotero.org/google-docs/?OndkLv)

[Taha, M., Shah, S. A. A., Afifi, M., Imran, S., Sultan, S., Rahim, F., & Khan, K. M. (2018). Synthesis, α-glucosidase inhibition and molecular docking study of coumarin based derivatives. *Bioorganic Chemistry*, *77*, 586–592. https://doi.org/10.1016/j.bioorg.2018.01.033](https://www.zotero.org/google-docs/?OndkLv)

[Tang, J.-J., Li, J.-G., Qi, W., Qiu, W.-W., Li, P.-S., Li, B.-L., & Song, B.-L. (2011). Inhibition of SREBP by a Small Molecule, Betulin, Improves Hyperlipidemia and Insulin Resistance and Reduces Atherosclerotic Plaques. *Cell Metabolism*, *13*(1), 44–56. https://doi.org/10.1016/j.cmet.2010.12.004](https://www.zotero.org/google-docs/?OndkLv)

[Taqvi, S., Ahmed Bhat, E., Sajjad, N., Sabir, J. S. M., Qureshi, A., Rather, I. A., & Rehman, S. (2021). Protective effect of vanillic acid in hydrogen peroxide-induced oxidative stress in D.Mel-2 cell line. *Saudi Journal of Biological Sciences*, *28*(3), 1795–1800. https://doi.org/10.1016/j.sjbs.2020.12.023](https://www.zotero.org/google-docs/?OndkLv)

[Taslimi, P., Caglayan, C., & Gulcin, İ. (2017). The impact of some natural phenolic compounds on carbonic anhydrase, acetylcholinesterase, butyrylcholinesterase, and α-glycosidase enzymes: An antidiabetic, anticholinergic, and antiepileptic study. *Journal of Biochemical and Molecular Toxicology*, *31*(12), e21995. https://doi.org/10.1002/jbt.21995](https://www.zotero.org/google-docs/?OndkLv)

[*The Potential Role of apigenin in Diabetes Mellitus | Auctores*. (n.d.). Retrieved April 11, 2023, from https://www.auctoresonline.org/article/the-potential-role-of-apigenin-in-diabetes-mellitus](https://www.zotero.org/google-docs/?OndkLv)

[Todaro, A., Cimino, F., Rapisarda, P., Catalano, A., Barbagallo, R., & Spagna, G. (2009). Recovery of anthocyanins from eggplant peel. *Food Chemistry*, *114*(2), 434–439. https://doi.org/10.1016/j.foodchem.2008.09.102](https://www.zotero.org/google-docs/?OndkLv)

[Tuli, H. S., Rath, P., Chauhan, A., Ramniwas, S., Vashishth, K., Varol, M., Jaswal, V. S., Haque, S., & Sak, K. (2022). Phloretin, as a Potent Anticancer Compound: From Chemistry to Cellular Interactions. *Molecules*, *27*(24), 8819. https://doi.org/10.3390/molecules27248819](https://www.zotero.org/google-docs/?OndkLv)

[Uğur, H., Çatak, J., Özgür, B., Efe, E., Görünmek, M., Belli̇, İ., & Yaman, M. (2022). Effects of different polyphenol-rich herbal teas on reducing predicted glycemic index. *Food Science and Technology*, *42*, e03022. https://doi.org/10.1590/fst.03022](https://www.zotero.org/google-docs/?OndkLv)

[Umeno, A., Horie, M., Murotomi, K., Nakajima, Y., & Yoshida, Y. (2016). Antioxidative and Antidiabetic Effects of Natural Polyphenols and Isoflavones. *Molecules*, *21*(6), 708. https://doi.org/10.3390/molecules21060708](https://www.zotero.org/google-docs/?OndkLv)

[Venu Gopal, J. (2013). Morin Hydrate: Botanical origin, pharmacological activity and its applications: A mini-review. *Pharmacognosy Journal*, *5*(3), 123–126. https://doi.org/10.1016/j.phcgj.2013.04.006](https://www.zotero.org/google-docs/?OndkLv)

[Vinothiya, K., & Ashokkumar, N. (2017). Modulatory effect of vanillic acid on antioxidant status in high fat diet-induced changes in diabetic hypertensive rats. *Biomedicine & Pharmacotherapy*, *87*, 640–652. https://doi.org/10.1016/j.biopha.2016.12.134](https://www.zotero.org/google-docs/?OndkLv)

[Voroneanu, L., Nistor, I., Dumea, R., Apetrii, M., & Covic, A. (2016). Silymarin in Type 2 Diabetes Mellitus: A Systematic Review and Meta-Analysis of Randomized Controlled Trials. *Journal of Diabetes Research*, *2016*, 1–10. https://doi.org/10.1155/2016/5147468](https://www.zotero.org/google-docs/?OndkLv)

[Wu, Q., Zhang, Y., Tang, H., Chen, Y., Xie, B., Wang, C., & Sun, Z. (2017). Separation and Identification of Anthocyanins Extracted from Blueberry Wine Lees and Pigment Binding Properties toward β-Glucosidase. *Journal of Agricultural and Food Chemistry*, *65*(1), 216–223. https://doi.org/10.1021/acs.jafc.6b04244](https://www.zotero.org/google-docs/?OndkLv)

[Xu, B., Li, Z., Zeng, T., Zhan, J., Wang, S., Ho, C.-T., & Li, S. (2022). Bioactives of Momordica charantia as Potential Anti-Diabetic/Hypoglycemic Agents. *Molecules*, *27*(7), 2175. https://doi.org/10.3390/molecules27072175](https://www.zotero.org/google-docs/?OndkLv)

[Xu, F., Yang, J., Negishi, H., Sun, Y., Li, D., Zhang, X., Hayashi, T., Gao, M., Ikeda, K., & Ikejima, T. (2018). Silibinin decreases hepatic glucose production through the activation of gut–brain–liver axis in diabetic rats. *Food & Function*, *9*(9), 4926–4935. https://doi.org/10.1039/C8FO00565F](https://www.zotero.org/google-docs/?OndkLv)

[Xu, Y., Tang, G., Zhang, C., Wang, N., & Feng, Y. (2021). Gallic Acid and Diabetes Mellitus: Its Association with Oxidative Stress. *Molecules*, *26*(23), Article 23. https://doi.org/10.3390/molecules26237115](https://www.zotero.org/google-docs/?OndkLv)

[Yan, Y., Zhou, X., Guo, K., Zhou, F., & Yang, H. (2020). Use of Chlorogenic Acid against Diabetes Mellitus and Its Complications. *Journal of Immunology Research*, *2020*, e9680508. https://doi.org/10.1155/2020/9680508](https://www.zotero.org/google-docs/?OndkLv)

[Yang, J.-P., He, H., & Lu, Y.-H. (2014). Four Flavonoid Compounds from *Phyllostachys edulis* Leaf Extract Retard the Digestion of Starch and Its Working Mechanisms. *Journal of Agricultural and Food Chemistry*, *62*(31), 7760–7770. https://doi.org/10.1021/jf501931m](https://www.zotero.org/google-docs/?OndkLv)

[Yang, J.-R., Luo, J.-G., & Kong, L.-Y. (2015). Determination of α-glucosidase inhibitors from ScutScutellaria baicalensis using liquid chromatography with quadrupole time of flight tandem mass spectrometry coupled with centrifugal ultrafiltration. *Chinese Journal of Natural Medicines*, *13*(3), 208–214. https://doi.org/10.1016/S1875-5364(15)30006-6](https://www.zotero.org/google-docs/?OndkLv)

[Yang, M., Wu, C., Zhang, T., Shi, L., Li, J., Liang, H., Lv, X., Jing, F., Qin, L., Zhao, T., Wang, C., Liu, G., Feng, S., & Li, F. (2022). Chicoric Acid: Natural Occurrence, Chemical Synthesis, Biosynthesis, and Their Bioactive Effects. *Frontiers in Chemistry*, *10*. https://www.frontiersin.org/articles/10.3389/fchem.2022.888673](https://www.zotero.org/google-docs/?OndkLv)

[Yokozawa, T., Cho, E. J., Park, C. H., & Kim, J. H. (2012). Protective Effect of Proanthocyanidin against Diabetic Oxidative Stress. *Evidence-Based Complementary and Alternative Medicine*, *2012*, 1–11. https://doi.org/10.1155/2012/623879](https://www.zotero.org/google-docs/?OndkLv)

[You, Q., Chen, F., Wang, X., Jiang, Y., & Lin, S. (2012). Anti-diabetic activities of phenolic compounds in muscadine against alpha-glucosidase and pancreatic lipase. *LWT - Food Science and Technology*, *46*(1), 164–168. https://doi.org/10.1016/j.lwt.2011.10.011](https://www.zotero.org/google-docs/?OndkLv)

[Yousefi, F., Mahjoub, S., Pouramir, M., & Khadir, F. (2013). Hypoglycemic activity of Pyrus biossieriana Buhse leaf extract and arbutin: Inhibitory effects on alpha amylase and alpha glucosidase. *Caspian Journal of Internal Medicine*, *4*(4), 763–767. https://www.ncbi.nlm.nih.gov/pmc/articles/PMC3841776/](https://www.zotero.org/google-docs/?OndkLv)

[Yuk, H. J., Lee, J. H., Curtis-Long, M. J., Lee, J. W., Kim, Y. S., Ryu, H. W., Park, C. G., Jeong, T.-S., & Park, K. H. (2011). The most abundant polyphenol of soy leaves, coumestrol, displays potent α-glucosidase inhibitory activity. *Food Chemistry*, *126*(3), 1057–1063. https://doi.org/10.1016/j.foodchem.2010.11.125](https://www.zotero.org/google-docs/?OndkLv)

[Yun, K.-J., Koh, D.-J., Kim, S.-H., Park, S. J., Ryu, J. H., Kim, D.-G., Lee, J.-Y., & Lee, K.-T. (2008). Anti-Inflammatory Effects of Sinapic Acid through the Suppression of Inducible Nitric Oxide Synthase, Cyclooxygase-2, and Proinflammatory Cytokines Expressions via Nuclear Factor-κB Inactivation. *Journal of Agricultural and Food Chemistry*, *56*(21), 10265–10272. https://doi.org/10.1021/jf802095g](https://www.zotero.org/google-docs/?OndkLv)

[Zarren, G., Shafiq, N., Arshad, U., Rafiq, N., Parveen, S., & Ahmad, Z. (2021). Copper-catalyzed one-pot relay synthesis of anthraquinone based pyrimidine derivative as a probe for antioxidant and antidiabetic activity. *Journal of Molecular Structure*, *1227*, 129668. https://doi.org/10.1016/j.molstruc.2020.129668](https://www.zotero.org/google-docs/?OndkLv)

[Zeng, L., Zhang, G., Lin, S., & Gong, D. (2016). Inhibitory Mechanism of Apigenin on α-Glucosidase and Synergy Analysis of Flavonoids. *Journal of Agricultural and Food Chemistry*, *64*(37), 6939–6949. https://doi.org/10.1021/acs.jafc.6b02314](https://www.zotero.org/google-docs/?OndkLv)

[Zhang, B., Sang, Y., Sun, W., Yu, H., Ma, B., Xiu, Z., & Dong, Y. (2017). Combination of flavonoids from Oroxylum indicum seed extracts and acarbose improves the inhibition of postprandial blood glucose: In vivo and in vitro study. *Biomedicine & Pharmacotherapy*, *91*, 890–898. https://doi.org/10.1016/j.biopha.2017.04.080](https://www.zotero.org/google-docs/?OndkLv)

[Zhang, B., Xing, Y., Wen, C., Yu, X., Sun, W., Xiu, Z., & Dong, Y. (2017). Pentacyclic triterpenes as α-glucosidase and α-amylase inhibitors: Structure-activity relationships and the synergism with acarbose. *Bioorganic & Medicinal Chemistry Letters*, *27*(22), 5065–5070. https://doi.org/10.1016/j.bmcl.2017.09.027](https://www.zotero.org/google-docs/?OndkLv)

[Zhang, Y., Gu, M., Cai, W., Yu, L., Feng, L., Zhang, L., Zang, Q., Wang, Y., Wang, D., Chen, H., Tong, Q., Ji, G., & Huang, C. (2016). Dietary component isorhamnetin is a PPARγ antagonist and ameliorates metabolic disorders induced by diet or leptin deficiency. *Scientific Reports*, *6*(1), 19288. https://doi.org/10.1038/srep19288](https://www.zotero.org/google-docs/?OndkLv)

[Zhao, C., Zhao, C., & Zhao, H. (2020). Defective insulin receptor signaling in patients with gestational diabetes is related to dysregulated miR-140 which can be improved by naringenin. *The International Journal of Biochemistry & Cell Biology*, *128*, 105824. https://doi.org/10.1016/j.biocel.2020.105824](https://www.zotero.org/google-docs/?OndkLv)

[Zheng, Y., Tian, J., Yang, W., Chen, S., Liu, D., Fang, H., Zhang, H., & Ye, X. (2020). Inhibition mechanism of ferulic acid against α-amylase and α-glucosidase. *Food Chemistry*, *317*, 126346. https://doi.org/10.1016/j.foodchem.2020.126346](https://www.zotero.org/google-docs/?OndkLv)
